# The complete and fully-phased diploid genome of a male Han Chinese

**DOI:** 10.1038/s41422-023-00849-5

**Published:** 2023-07-14

**Authors:** Chentao Yang, Yang Zhou, Yanni Song, Dongya Wu, Yan Zeng, Lei Nie, Panhong Liu, Shilong Zhang, Guangji Chen, Jinjin Xu, Hongling Zhou, Long Zhou, Xiaobo Qian, Chenlu Liu, Shangjin Tan, Chengran Zhou, Wei Dai, Mengyang Xu, Yanwei Qi, Xiaobo Wang, Lidong Guo, Guangyi Fan, Aijun Wang, Yuan Deng, Yong Zhang, Jiazheng Jin, Yunqiu He, Chunxue Guo, Guoji Guo, Qing Zhou, Xun Xu, Huanming Yang, Jian Wang, Shuhua Xu, Yafei Mao, Xin Jin, Jue Ruan, Guojie Zhang

**Affiliations:** 1 Center for Genomic Research, International Institutes of Medicine, The Fourth Affiliated Hospital, Zhejiang University School of Medicine, Yiwu, Zhejiang China; 2 Center for Evolutionary & Organismal Biology, & Women’s Hospital, Zhejiang University School of Medicine, Hangzhou, Zhejiang China; 3 BGI-Shenzhen, Shenzhen, Guangdong China; 4 BGI Research-Wuhan, BGI, Wuhan, Hubei China; 5 Shenzhen Branch, Guangdong Laboratory for Lingnan Modern Agriculture, Genome Analysis Laboratory of the Ministry of Agriculture and Rural Affairs, Agricultural Genomics Institute at Shenzhen, Chinese Academy of Agricultural Sciences, Shenzhen, Guangdong, China; 6 Liangzhu Laboratory, Zhejiang University Medical Center, Hangzhou, Zhejiang China; 7 Institute of Crop Science & Institute of Bioinformatics, Zhejiang University, Hangzhou, Zhejiang China; 8 Bio-X Institutes, Key Laboratory for the Genetics of Developmental and Neuropsychiatric Disorders, Ministry of Education, Shanghai Jiao Tong University, Shanghai, China; 9 College of Life Sciences, University of Chinese Academy of Sciences, Beijing, China; 10 Innovation Center of Yangtze River Delta, Zhejiang University, Hangzhou, Zhejiang China; 11 Life Sciences Institute, Zhejiang University, Hangzhou, Zhejiang China; 12 BGI-Qingdao, BGI-Shenzhen, Qingdao, Shandong China; 13 BGI-Hangzhou, Hangzhou, Zhejiang China; 14 School of Medicine, Zhejiang University, Hangzhou, Zhejiang China; 15 State Key Laboratory of Genetic Engineering, Center for Evolutionary Biology, Collaborative Innovation Center for Genetics and Development, School of Life Sciences, Fudan University, Shanghai, China; 16 Human Phenome Institute, Zhangjiang Fudan International Innovation Center, and Ministry of Education Key Laboratory of Contemporary Anthropology, Fudan University, Shanghai, China; 17 Jiangsu Key Laboratory of Phylogenomics & Comparative Genomics, International Joint Center of Genomics of Jiangsu Province School of Life Sciences, Jiangsu Normal University, Xuzhou, Jiangsu China; 18 Department of Liver Surgery and Transplantation Liver Cancer Institute, Zhongshan Hospital, Fudan University, Shanghai, China; 19 Center for Excellence in Animal Evolution and Genetics, Chinese Academy of Sciences, Kunming, Yunnan China; 20 State Key Laboratory of Genetic Resources and Evolution, Kunming Institute of Zoology, Chinese Academy of Sciences, Kunming, Yunnan China

**Keywords:** Genomic analysis, Population genetics

## Abstract

Since the release of the complete human genome, the priority of human genomic study has now been shifting towards closing gaps in ethnic diversity. Here, we present a fully phased and well-annotated diploid human genome from a Han Chinese male individual (CN1), in which the assemblies of both haploids achieve the telomere-to-telomere (T2T) level. Comparison of this diploid genome with the CHM13 haploid T2T genome revealed significant variations in the centromere. Outside the centromere, we discovered 11,413 structural variations, including numerous novel ones. We also detected thousands of CN1 alleles that have accumulated high substitution rates and a few that have been under positive selection in the East Asian population. Further, we found that CN1 outperforms CHM13 as a reference genome in mapping and variant calling for the East Asian population owing to the distinct structural variants of the two references. Comparison of SNP calling for a large cohort of 8869 Chinese genomes using CN1 and CHM13 as reference respectively showed that the reference bias profoundly impacts rare SNP calling, with nearly 2 million rare SNPs miss-called with different reference genomes. Finally, applying the CN1 as a reference, we discovered 5.80 Mb and 4.21 Mb putative introgression sequences from Neanderthal and Denisovan, respectively, including many East Asian specific ones undetected using CHM13 as the reference. Our analyses reveal the advances of using CN1 as a reference for population genomic studies and paleo-genomic studies. This complete genome will serve as an alternative reference for future genomic studies on the East Asian population.

## Introduction

The current human genomic studies are predominately on populations of European ancestry, with over 85% in individuals of European descendent.^1,2^ The lack of ethnic diversity in genomic studies has hampered a complete understanding of the genomic components of global human populations and limited the translation of genetic research to clinical medicine in underrepresented populations.^3,4^ The bias in genomic studies is also reflected in wide use of the current reference genome GRCh38 assembly, which was obtained from a composite DNA originating from a few different donor individuals mainly from Africa or Europe, for almost all genomic studies. This reference genome system has been the foundation of all genomic databases, provides a universal scaffold for gene annotation, variant calling and functional analyses, and is an essential resource for biomedical research. However, the GRCh38 does not represent all genomic components in all populations, leading to the reference bias wherein reads from non-reference alleles would be missed during the alignment to the reference genome.^5–7^ The reference bias would also lead to a failure in identifying pathogenic mutations in minor or pathogenic reference alleles^8–10^ and wrong conclusions in allele sharing, heterozygosity estimates and inference of archaic ancestry.^11^ Therefore, there is a need to assess to what extent the current reference genome GRCh38 and the European descendent CHM13, the recently released telomere-to-telomere (T2T) genome, differs from a genome of another ethnic group and the genomic features contribute to the reference bias.

The Asian population, including diverse ethnic groups adapted to high altitude, forest, desert, grassland, and coastal environments, accounts for > 60% of the world population.^12–14^ Although several genomic programs, such as the Pan-Asian SNP Consortium, GenomeAsia, Han100K, Chinese Pangenome Consortium, 3.5KJPNv2, Korea1K, and IndiGen, have started focusing on Asian populations, genomic studies on them are marginal considering their population size and genetic diversity.^15–21^ Research on the Asian population has relied on the mixed reference genome with relatively few Asian genetic background; therefore, the inherent reference bias might affect the accuracy of variant calling in these efforts. Consequently, it is crucial to produce a complete reference genome from Asian descendent and investigate the structural differences between the Asian genomes and the current reference (GRCh38). Besides, recent paleo-genomic studies have shown that the Aboriginal Australasians and Native Americans share a closer genetic relationship with the East Asians than present-day Europeans.^22,23^ Thus, a reference genome from an Asian descendent will also be valuable for the studies of Aboriginal Australasians and Native Americans, another two populations underrepresented in genomic research, especially on Asian-specific features inherited by these populations.

The human genome project has led to continuous improvement in the assembly of reference genomes over the last two decades. Recently, the T2T consortium produced the first complete gapless T2T human reference genome from the CHM13hTERT (CHM13) human cell line, filling the gaps in the previous linear reference genome using state-of-the-art sequencing technologies.^24^ This CHM13 cell line was originally isolated from a hydatidiform mole with two nearly identical haploid complements. Therefore, this genome differs from a biologically normal personal genome with two sets of chromosomes inherited from the parents. The advances in sequencing technologies and assembly algorithms have helped produce high-quality diploid human genomes^25–29^; however, a complete diploid human genome with both haploid genomes assembled at T2T level has not been achieved. Here, we present the complete de novo diploid genome of an East Asian (EAS) male with 44 autosomes and XY chromosomes, including two sets of chromosomes fully phased and assembled at the T2T level. This genome allows us to investigate the differences in the end-to-end components between CN1 and CHM13 genomes and assess the impact of the reference bias on genetic diversity studies in Asian and other closely related populations.

## Results

### T2T diploid human genome assembly

A healthy male Chinese individual CN1 from Hubei, China with self-reported Southern Chinese ancestry was selected to produce the genome (see Materials and Methods). We generated PacBio HiFi (69×), Nanopore Ultra-long Oxford nanopore technology (ONT) reads (79×), Hi-C (116×) and short-read data from MGISEQ (97×) and Illumina (108×) platform for this individual (Supplementary information, Table S1). The parental samples were also sequenced using the PacBio HiFi and MGISEQ technologies (Supplementary information, Table S1). Since no assembler can directly produce T2T assembly, here, we first employed the hifiasm^27^ and verkko^28^ assemblers to generate the haplotype assemblies using the trio mode. For each haplotype, we selected the more continuous contigs from the two assemblies to represent. This results in a maternal assembly with 30 gaps and paternal assembly with 39 gaps. Subsequently, gap closing was performed using TGS-Gapcloser^30^ with the hifiasm assembly, the Flye^31^ assembly constructed with the trio-binned ONT reads, and the trio-binned ONT reads (Supplementary information, Table S2). After this stage, the maternal and paternal genomes had only 9 and 7 gaps, respectively, mainly located at the centromere. The gaps in each haploid genome were further closed out by mapping the ONT reads against the assembly and manually filled with the best aligned reads (Supplementary information, Fig. S1). We also performed local assembly to fill a gap in the short distal arm of chr15 (~2.17 Mb) that was missed in the initial assembly (Supplementary Notes). Meanwhile, for the gaps in the heterochromatic regions on the Y chromosome, we extracted the ONT reads that could span multiple Y-linked scaffolds, manually arranged these scaffolds based on the number of ONT reads supporting the scaffold orientation, and finally filled the remaining gaps with the ONT reads.

We further performed five rounds of assembly polishing using the binned ONT reads and all HiFi and MGISEQ reads (Supplementary Notes and Supplementary information, Table S3). During this step, we mapped all the MGISEQ short reads and the HiFi reads onto each haploid genome and called the small variants with Deepvariant^32^ from these assemblies. We also mapped the binned ONT reads to their respective haploid genome and carried out small variant calling with PEPPER-Margin-DeepVariant.^33^ We applied the homozygous variants from both callers during polishing to correct the sequencing errors in the assembly. We performed manual curation for the assemblies on regions where structural variants (SVs; ≥ 50 bp) could be detected with binned HiFi and ONT reads by visualizing the alignments. If the SVs were supported by both HiFi and ONT reads, we would correct the local assemblies coordinately; the coordinates of these SVs were also manually curated based on the alignments. Besides, we manually examined and curated regions with abnormal coverage and soft-clipped read mapping signals on the non-centromeric regions using the binned HiFi and ONT reads (Supplementary information, Fig. S2). To make it easier for further effort for improvement of the assembly, we added issue label on regions with abnormal sequencing depth, which include 361 (10.52 Mb) in CN1.mat and 674 (14.16 Mb) in CN1.pat (https://github.com/T2T-CN1/CN1-issues).

**Fig. 1:**
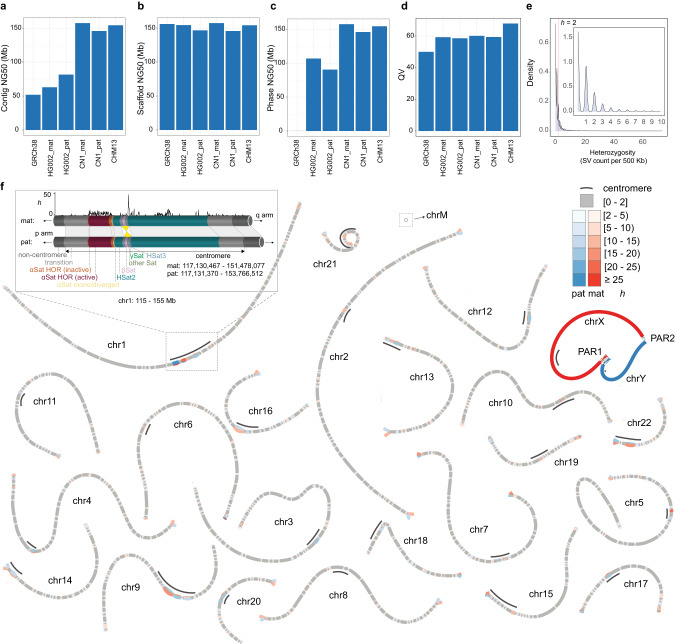
Haplotype-resolved assembly of CN1 diploid genome. **a**–**d** The contig NG50, scaffold NG50, phase NG50, and QV of GRCh38, HG002, CN1, and CHM13. **e** Whole-genome distribution of the heterozygosity rate (*h*). The heterozygosity rate is calculated as the SV count in each 500 kb window. **f** Visualization of the heterozygous regions between two haplotypes using bubbles. Here, *h* = 2 was set as the threshold for displaying bubbles. The homozygous regions are shown as single paths (grey), and the heterozygous regions at each heterozygosity rate are marked as bubbles. Regions with different *h* are shown in different shades. Centromeres (black lines) carry much higher *h* than other regions. The insert plot shows the structural variations in the centromere of chr1. αSat and HSat2 are the most divergent (window = 50 kb, step = 10 kb). An inversion, covering βSat and γSat, is shown between the paternal and maternal genomes (light yellow).

The final diploid T2T Han reference genome (CN1v0.8.1) has NG50 of 157.4 Mb and 145.8 Mb for maternal and paternal genomes, respectively (Fig. 1a–c) and a good consensus base call (quality value (QV) of 60.10 for CN1.mat and 59.36 for CN1.pat) (Fig. 1d; Supplementary information, Table S4). Although the QV of the CN1 genome is relatively lower than that of the uniformly homozygous CHM13v2.0 genome,^24^ it is close to that of the haploid genome of the HG002 cell line^26^ (Supplementary information, Fig. S3 and Table S4) and QV60 (one error per megabase) threshold proposed by the Vertebrate Genome Project.^26,34^ Further, by comparing the linear genomes of two complete haplotypes, we detected ~2.6 million single nucleotide variants (SNVs), ~311,000 small insertions or deletions (indels) (< 50 bp), and 16,519 SVs (≥ 50 bp). The heterozygosity rate between the two CN1 haplotype genomes calculated using SV count (Fig. 1e, Materials and Methods) revealed the most diverse regions in the centromeres, especially in alpha satellite (αSat) and human satellites (HSat) (Fig. 1f). The heterozygosity rate in the centromeric regions is at least five times higher than that in the non-centromeric regions (on average 7.79 vs 1.44 SVs per 500 kb, Supplementary information, Fig. S4 and Supplementary Notes). We further merged the diploid genome and generated a haploid T2T reference genome CN1 representing the Han group by selecting the better assembled version for each chromosome to represent (Fig. 1; Supplementary information, Table S5). Of the 24 chromosomes, twelve from the CN1 genome showed a QV higher than the HG002 genome (Supplementary information, Table S4). All 97 HG002.mat and 99 HG002.pat gaps have been filled in CN1, with most of the sequences residing in the centromeric regions^26^ (Supplementary information, Fig. S3).

Generally, the lack of annotation hinders the application of a new reference genome. Therefore, we fully annotated the protein-coding genes, non-coding genes, regulatory elements, and repetitive elements and located 98.28% of single nucleotide polymorphisms (SNPs) in the dbSNPs database (v156) for this new reference genome (Supplementary information, Fig. S5, Tables S6, S7 and Supplementary Notes). Of the 19,846 protein-coding genes annotated for GRCh38.p14 in RefSeq (v110), only 44 appeared missing in CN1, including 23 also missing in CHM13 and 21 aligning poorly on the new genome (Supplementary information, Table S8). These poorly aligned protein-coding genes encode the ubiquitin processing protease enzymes, immunoglobulins, and T-cell receptors; they are the known, highly diverse genes with copy number variations (CNVs) between individuals.^35,36^ We identified 10 more protein-coding genes not annotated in RefSeq v110 and CHM13. The RNA-seq (or scRNA-seq) data of Chinese human tissues provide evidence supporting annotation of these genes (Supplementary information, Table S9). Most of these newly annotated genes distributed in the centromere regions might have been missed due to the sequencing gaps in GRCh38 or individual variations. Further, based on the LiftOver approach, we mapped 99.12% (1,057,906 of 1,067,338) of previously annotated regulatory elements by Encyclopedia of DNA Elements (ENCODE)^37^ on CN1. We also identified 145,472 new regulatory elements in CN1, including 84,423 promoter-like regions and 61,049 enhancer-like regions, based on the ChIP-seq data of 6 different histone methylation marks from 21 Chinese human tissues (Supplementary information, Table S10). We finally developed a genome browser for this reference genome incorporating all the annotation information (https://genome.zju.edu.cn/) for public access. This genome browser allows users to upload additional genetic evidence, improving the quality and accuracy of the annotation for the Chinese reference genome.

### Variation of peri/centromere regions among reference genomes

**Fig. 2:**
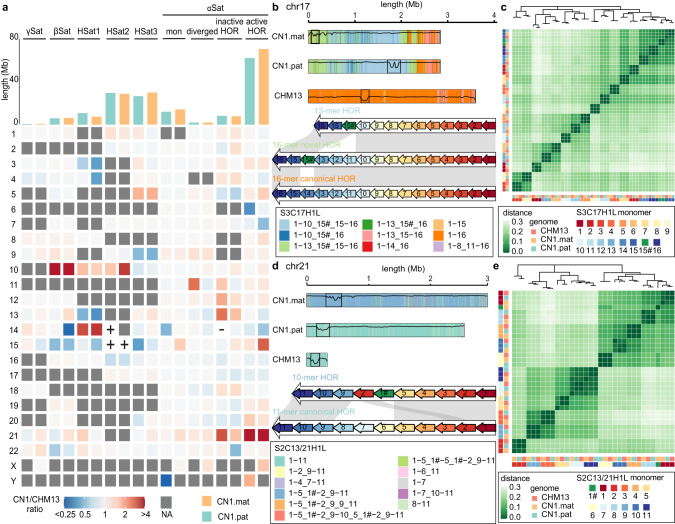
Variations in the peri/centromeric regions among the CN1.mat, CN1.pat and CHM13. **a** Heatmap shows the length differences of each component of the pericentromeric region of the chromosomes between the two haploid genomes of CN1 and CHM13. “+” indicates presence in CN1 haploid genome but absence in CHM13; “−” indicates absence in CN1 haploid but presence in CHM13. The top bar plot shows each satellite’s length in CN1. NA means that the satellite is absent in both CN1 and CHM13. **b** Composition of the active HOR in the three haploids of chr17. The canonical 16-mer S3C17H1L.1-16 is the dominant HOR in CHM13, while two novel HORs, S3C17H1L.1-13_15#_15-16 and S3C17H1L.1-10_15#_15-16, are the dominant forms in CN1. Each color box represents one HOR SV. The DNA methylation level (ranging from 0 to 1) is plotted with the line along each active HOR, and the identified CDRs are delineated by boxes. **c** Clustering the monomer consensus sequences of the active HOR in the three haploids of chr17 according to the monomer consensus sequence alignment. The novel monomer S3C17H1L.15# clusters with the canonical S3C17H1L.1.15, with substantial sequence divergence. Shade indicates the p-distance between every two monomer consensus sequences. **d** Composition of the active HOR in the three haploids of chr21. The canonical 16-mer S2C13/21H1L.1-16 dominates CHM13 and CN1 paternal chr21, while the novel 10-mer HOR S2C13/21H1L.1-5_1#-2_9-11 is dominant in the CN1 maternal chr21. The DNA methylation level (ranging from 0 to 1) is plotted along each active HOR, and the identified CDRs are delineated by boxes. Color boxes represent HOR SVs. **e** Clustering of monomer consensus sequences of the active HOR in the three haploids of chr21 according to the monomer consensus sequence alignment. Shade indicates the p-distance between every two monomer consensus sequences.

The centromere and the pericentromeric regions are characterized by abundance of long and highly identical tandem repeats called satellite DNA.^38^ The detailed structure of the peri/centromeric regions was revealed only recently in the CHM13 reference genome.^39^ The diploid CN1 genome obtained in this study allowed us to explore the structural variations in the centromere, which have not been explored. The coverage evaluation was made by two software to avoid the issue regions involved in downstream analysis (Supplementary information, Figs. S6, S7). The analyses showed that the peri/centromeric regions of the individual genome occupy 12.7% and 12.5% of the maternal and paternal genomes, respectively (Supplementary information, Table S11), compared to 13.5% in CHM13. The repeat annotation for the centromere revealed a conserved repeat composition in CN1 and CHM13, both of which mainly consisted of αSat, βSat, γSat, and HSat1-3 (e.g., chr1 is shown in Fig. 1f). Generally, these individual αSat monomers organize into highly homogeneous units called higher-order repeats (HORs), which include active HOR, inactive HOR, divergent HOR, and other monomeric αSat.^40^ Further comparison of satellites showed an overall conserved pattern for the satellite composition in each chromosome; however, the length of αSat varied among the chromosomes (Fig. 2a). The most striking difference was observed on chr21, where the αSat in CN1 was about seven-fold longer than that in CHM13, probably due to the expansion of HOR in CN1 chr21. Other satellites also showed length differences in each chromosome. The largest size difference was detected for HSat3 in chr9, which is 27.96 Mb long in CHM13 but only 11.89 Mb and 12.74 Mb long in CN1 maternal and paternal chromosomes, respectively.

We further investigated the differences in the arrangement of the satellite arrays in the pericentromeres among the CN1 maternal and paternal genomes and CHM13 genome. Overall, the arrangement of the major satellite types in the three genomes appeared highly conserved, with some rearrangements detected in specific chromosomes (Fig. 2a). For instance, the CN1 paternal chr14 has no inactive HORs but contains a 2.1 kb long HSat2, which is missing in the maternal chr14 and CHM13 (Supplementary information, Fig. S8). Both maternal and paternal chr15 of CN1 has ~2 kb long HSat2, which is absent in CHM13 (Supplementary information, Fig. S9). Besides, the conserved monomer order was detected in active HORs with chr17 and chr21 as exceptions. The active HOR of CHM13 chr17 has a dominant 16-mer canonical HOR and 12-, 13-, and 15-mer variants.^39^ However, in both alleles of CN1 chromosome 17, the major component is a 13-mer HOR, a variant of a novel 16-mer HOR (Fig. 2b). In contrast to the canonical 16-mer HOR that prevails in CHM13, the novel one in CN1 is introduced by a new monomer, which was identified using a phylogeny-based method (Fig. 2b, c). We confirmed the presence of this new monomer in both CN1 maternal and paternal genomes with the raw sequencing reads and named it S3C17H1L.15# (Supplementary information, Fig. S10). Moreover, we found that the dominant 13-mer variant is identical to the novel 16-mer HOR, except that this 13-mer variant lacks the three successive monomers (Fig. 2b). Meanwhile, in both CHM13 and CN1 paternal chr21, the active HOR arrays are mainly represented by different copy numbers of the same canonical 11-mer HOR S2C13/21H1L.1-11. On the other hand, in the CN1 maternal chr21, the main component of the active HOR array is a 10-mer novel HOR with 11-mer canonical HORs at both ends of its active HOR array (Fig. 2d, e). This 10-mer novel HOR is a variant of an 11-mer HOR with its middle three monomers replaced by S2C13/21H1L.2 and a novel monomer (Fig. 2d, e; Supplementary information, Fig. S11). These findings revealed the variations of centromere components among personal genomes that have hidden in most previous studies.

We next investigate the methylation pattern difference over the centromere regions among the alleles. Similar to the findings in CHM13,^39,41^ we observed a drop in the DNA methylation level in the subregion of the active HORs compared to their flanking regions in all chromosomes (Supplementary information, Figs. S12–S16, Table S12), termed as centromeric dip region (CDR). Generally, CDRs are co-localized with CENP association sites and therefore are associated with kinetochore binding.^41–43^ The HOR composition of CDR appeared to be well consistent among the two alleles of CN1 and CHM13 in most chromosomes, except chr17 and chr21 (Fig. 2b, d; Supplementary information, Fig. S17, Tables S13, S14), probably due to the differences in the major components in the active HOR.

### Structural variations in CN1 genome compared with CHM13

**Fig. 3:**
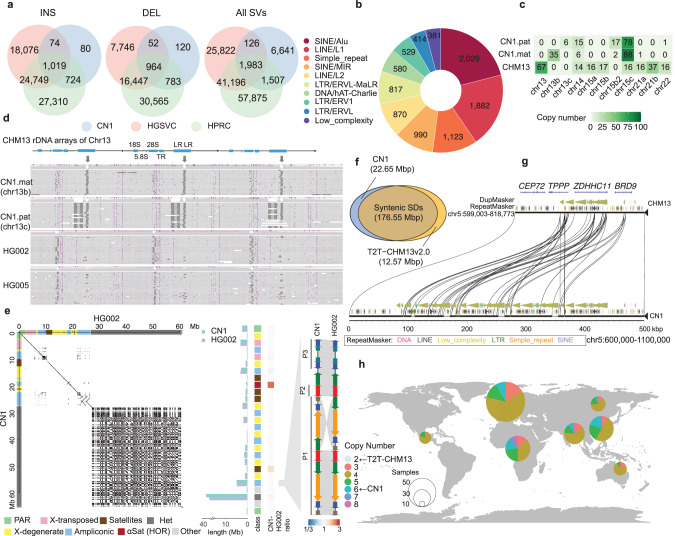
SVs between CN1 and CHM13. **a** Comparison of SVs between CN1 and CHM13 based on HGSVC and HPRC databases. **b** Repeat annotation of novel SVs, top 10 for plot. **c** Copy number of rDNA models across three haploid genomes, CN1.mat, CN1.pat, and CHM13. **d** Illustration of chr13 rDNA model in CN1, HG002, and HG005. CHM13 chr13 rDNA model is shown on the top, and the ONT read alignments in the different haploids/individuals are shown below. Compared to the CHM13 reference, the CN1.mat chr13 rDNA model has one 4.4 kb deletion in LR, and the CN1.pat chr13 rDNA model has an additional 1 kb deletion in LR. In HG002 and HG005, only a few copies of rDNA array contain the 1.1 kb deletion in LR. Each row represents a read alignment, with insertions shown as purple triangles and deletions shown as dark lines. **e** Comparison of CN1-Y and HG002-Y. The dot plot on the left shows the overall synteny between the two Y chromosomes, with a large inversion in the last ampliconic region. The middle barplot shows the size comparison for the different subregions on the two Y chromosomes. The major size differences are found in centromere, DYZ19, and heterochromatin. The synteny plot on the right shows the largest inversion on Y in one arm of palindrome P1 in the last ampliconic region. P1, P2 and P3 indicate palindromes 1, 2 and 3, respectively. **f** Venn diagram shows the syntenic and non-syntenic SDs (except chrY) of CN1 (blue) and CHM13 (orange). **g** Syntenic comparison of *ZDHHC11* and its flanking region between CN1 and CHM13 genomes. The copy number of *ZDHHC11* is expanded in CN1. **h** Global map shows the distribution of *ZDHHC11* copy number across 317 human samples from the Simons Genome Diversity Project (SGDP). Color indicates the *ZDHHC11* copy number and the size of the circles indicates the individual number examined in each super-population. There are two and six copies of *ZDHHC11* in CN1 and CHM13, respectively.

Apart from the differences in the centromeric and heterochromatic regions, a comparison between the CN1 and CHM13 whole genomes showed at least 11,281 and 11,012 SVs in the maternal and paternal genomes, spanning about 24.60 Mb and 26.14 Mb, respectively (Supplementary information, Tables S15, S16). Over 99% of these variants are indels. Among these, an inversion spanning 4.27 Mb in chr8 was identified as the longest SV, a common polymorphism in the human population,^44,45^ and a 1.61 Mb long inversion in the chrY palindrome P1 as the second longest SV. We found that about half of these SVs (6641) are novel and absent in Human Genome Structural Variation Consortium (HGSVC)^46^ and Human Pangenome Reference Consortium (HPRC),^47^ the two major long read-based SV databases for global populations (Fig. 3a; Supplementary information, Table S17). Subsequent repeat annotation found a substantial number of short interspersed nuclear elements (SINEs), long interspersed nuclear elements (LINEs), and long terminal repeats (LTRs) among these SVs (Fig. 3b). We observed that SINE/Alu accounted for the most common type of SV, indicating that Alu is one of the most variable repeat elements in the human genome.^48,49^ Of note, most novel SVs (6441) are CNV, duplication, and inversion since HGSVC and HPRC only cover insertion and deletion. This suggests that the T2T genome comparison is an effective way to capture all forms of SVs.

Previous studies based on incomplete genome assembly and raw sequencing reads have identified up to 6.4–20.0 Mb novel sequences in individual genomes that are absent in the reference genome.^50–53^ The complete CN1 genome further allowed us to assess novel sequences absent in CHM13 for the first time at the T2T level. We identified and characterized 429 kb of novel sequences in CN1 with an N50 of 1080 bp and an average length of 606 bp (Supplementary information, Table S18). The novel sequences in each chromosome are strongly correlated with the chromosome length (*R*^2^ = 0.7453*, P* = 1.14e–07; Supplementary information, Fig. S18). We found that 227 genes overlapped with these novel sequences and the coding regions of 3 genes (*ZNF512B*, *FAM118A*, and *SLC25A24*) overlapped with the novel sequences (Supplementary information, Table S19). Besides, 122 regular elements were identified in these novel sequences (Supplementary information, Table S20). The longest novel sequence identified in this study was a 4371 bp long insertion located at the end of chr13 (CN1 chr13:23,600,832-23,605,203). By mapping novel sequences to HPRC assemblies, the EAS and African (AFR) samples shared more novel sequences than other populations (Supplementary information, Fig. S19). We found that 43.5% of novel sequences (186,580 bp) were shared in all EAS samples, but none was unique to EAS, and 35.5% of novel sequences existed in most all examined primates (macaque, marmoset, gorilla, bonobo, chimpanzee, hamadryas baboon, pileated gibbon).

Generally, due to the tandem duplications of short DNA sequences for hundreds of copies, rDNA arrays on genomes are hard to assemble. The rDNA sequences were filled by rDNA units with estimated copies in current assemblies (Supplementary Notes). To investigate rDNA variation patterns between individuals, we aligned ONT reads to CHM13 rDNA arrays and compared their differences in unit composition (Supplementary Notes). An earlier study estimated that the CHM13 diploid genome contains 400 copies of rDNA using digital droplet PCR (ddPCR).^24^ In contrast, the present study estimated that the CN1 diploid genome contains only 250 copies (Supplementary information, Table S21). Further, based on the coverage of the binned ONT reads, we estimated about 132 and 117 copies of rDNA arrays in the maternal and paternal genomes, respectively (Fig. 3c), indicating a high variation in the rDNA arrays among the individual haploid genomes.^54^ We found four (chr14, chr15c, chr21a, chr22) out of the eight DNA models of CHM13 rDNA arrays in CN1. The detailed analysis detected a 1.1 kb deletion in the long repeat (LR) region in CN1.mat chr13 and an extra 4.4 kb deletion in another LR region in CN1.pat chr13 compared with CHM13 (Fig. 3d). The 1.1 kb deletion, which can also be detected in HG002 or HG005 with few copies, was the predominant model in CN1.mat chr13. However, we could not see the paternal 4.4 kb deletion in other individual genomes. These results suggest that the LR regions are highly diverse among haplotypes. Meanwhile, the remaining models are quite conserved between the two genomes, with differences in copy number among the chromosomes (Fig. 3c). We only detected two models (chr15b2, c) in the chr15 of CN1 and three models (chr15a, b, c) with similar copy numbers (15:16:16) in chr15 of CHM13. In addition, chr15b2 is a novel model, with a 3.5 kb deletion in the first LR compared to chr15b (Supplementary information, Fig. S20). We detected chr21a only in CN1.mat and both CN1 haploids have lost chr21b.

### Structural variations in Y chromosomes

Assembling the Y chromosome is challenging due to its highly repetitive nature, especially in the centromere and the heterochromatic region. The sequences of these regions remained unknown for long, even in the GRCh38 genome, and were revealed only recently.^55,56^ Our CN1 genome assembly presents a T2T gapless Y chromosome from a male Han Chinese, which enables us to compare the structural variations in Y chromosome among individuals. Overall, the CN1 Y chromosome (CN1-Y) and HG002 Y chromosome (HG002-Y) showed a good synteny (Fig. 3e). We detected 99 insertions, 99 deletions, and 1 inversion between the two Y chromosomes outside the centromere and the heterochromatic region. One large inversion, spanning almost 1.6 Mb at the arm of the palindrome P1, was found between HG002-Y and CN1-Y^56^ (Fig. 3e). A recent study showed that this SV was found only in the Y chromosome haplogroup J where HG002-Y belongs and possibly evolved in the ancient Near East.^57^ Among the 27 subregions in the Y chromosome, four (centromere, DYZ19, heterochromatic region, and one ampliconic region) are highly variable (Supplementary information, Table S22). Compared to HG002, CN1-Y has longer centromere (+103.32%, +327,860 bp), DYZ19 (+32.31%, +85,906 bp) and the heterochromatic region (+8.38%, +2,904,207 bp), but shorter second ampliconic region (counting from PAR1), which harbors the *TSPY* array (−7.06%, −222,644 bp). DYZ19 amplification, due to the duplication of a 40–120 kb region (red block, Supplementary information, Fig. S21), is the main reason for the centromere expansion in CN1-Y. Notably, DYZ19 showed the lowest identity among the subregions (96% compared to 99% of the other subregions, Supplementary information, Table S22). The DYZ2 expansion (consisting of HSat1B) caused a size difference in the heterochromatic region in CN1 (Supplementary information, Table S23, Fig. S22). Besides, CN1 has fewer *TSPY* copies (35) compared to HG002 (46), while the two Y chromosomes have identical copy numbers of all other protein-coding genes (Supplementary information, Table S24).

### Segmental duplications in the CN1 genome

Further, to investigate the differences in the segmental duplications (SDs) between CHM13 and CN1, we utilized the same methodology used by Vollger et al.^58^ We identified a total of 237 Mb nonredundant SDs in CN1, of which 44.45 Mb were located on chrY (Supplementary information, Table S25). About 22.65 Mb of these SDs could not be syntenically LiftOvered (except chrY) onto CHM13 (Fig. 3f), with 18.28 Mb of these SDs located at the centromeric regions (Supplementary information, Tables S26–S28). These unLiftOvered SDs span several disease-associated genes, such as *DEFB4A* and *POTEB*.

The complete genome of the Han Chinese individual allowed us to assess which reference genome is more suitable for estimating CNVs in Chinese populations. We first identified 2398 protein-coding genes with CNVs in 301 Chinese samples of the 1000 Genomes Project (1KGP). We further compared the CNVs of these genes at the population level to determine which reference (CHM13 or CN1) is more likely to represent the major allele in the population. We found 1365 and 1033 genes with similar copy numbers in the population using CN1 and CHM13 as reference, respectively, indicating that CN1 is a better reference (two-sided *t*-test, *P* = 1.668e–09; Supplementary information, Table S29). Here, the 1365 genes included many related to various diseases (e.g., *ZDHHC11*, *DBET*, and *AMY1A*; Supplementary information, Fig. S23). For instance, the copy number of *ZDHHC11*, an immune-related gene that regulates the host’s defense response to viruses, such as SARS-CoV-2,^59,60^ was higher in CN1 (~6 copies) than in CHM13 (~2 copies), which was confirmed based on syntenic comparison and short-read genotyping (Fig. 3g). However, it was unclear whether the copy number of *ZDHHC11* represents the ‘true’ copy number in different ethnic groups. Detailed analysis of the *ZDHHC11* copy number in 317 human samples from the Simons Genome Diversity Project (SGDP)^61^ showed that the copy number in CN1 is a more accurate representation for this gene in most populations, particularly the East Asian, South Asian, and African populations (Fig. 3h; Supplementary information, Fig. S24).

### CN1 accelerated regions

We further examined the accelerated sequences in CN1 compared with CHM13 and HG01891 (an individual of African ancestry) from HPRC at a 20 bp resolution (Materials and Methods). A total of 8120 regions spanning 251 kb were identified as accelerated regions in the CN1 allele that accumulated with significantly higher substitution rate than the neutral rate (estimated from four-fold degenerate sites) (Supplementary information, Table S30), with an average length of 31 bp. In these CN1 accelerated regions, ~44,173 SNVs contributed to the difference between CN1 and the other two genomes (i.e., CHM13 and HG01891). As expected, the SNV density in the CN1 accelerated regions (168.03/kb) was significantly higher than that in the other CN1-CHM13 syntenic regions (0.048/kb) (Wilcoxon rank-sum test, *P* < 2.2e–16). Interestingly, we found that the SNP density in these CN1 accelerated regions within the EAS population (0.0558 SNP/bp) is lower compared to those within European (EUR) (0.05697 SNP/bp) and AFR (0.06469 SNP/bp) populations (EAS vs EUR, *P* = 1.841e–05; EAS vs AFR, *P* < 2.2e–16; one-sided paired Wilcoxon rank-sum test). This indicated that some of the CN1 accelerated regions if not all might have been under natural selection within EAS population. We found that these CN1 accelerated regions overlap with 2396 protein-coding genes, including 39 with differences in amino acids between CN1 and CHM13 (Supplementary information, Table S31). Among the 39 genes harbored in CN1 accelerated regions, fifteen are immunity-related genes, including five human leukocyte antigen genes (*HLA-A*, *-B*, and *-C*, *-DQA1* and *-DRB4-2*). HLAs encode the cell surface proteins that are part of the major histocompatibility complex (MHC) and are involved in immune response and suppression.^62^ Other genes, including *USP17L26*, *USP17L27* and *GAGE2A_2*, are involved in cellular response to viral infection.^63^ Of note, two genes *GOLGA6L4* and *LOC102724117-2* have been reported to be associated with waist circumference.^64^ The accelerated pattern in CN1 allele indicates potential selection signals for the population carrying these alleles. How the amino acid differences obtained in these accelerated regions affect gene functions would be of interest for further study.

**Fig. 4:**
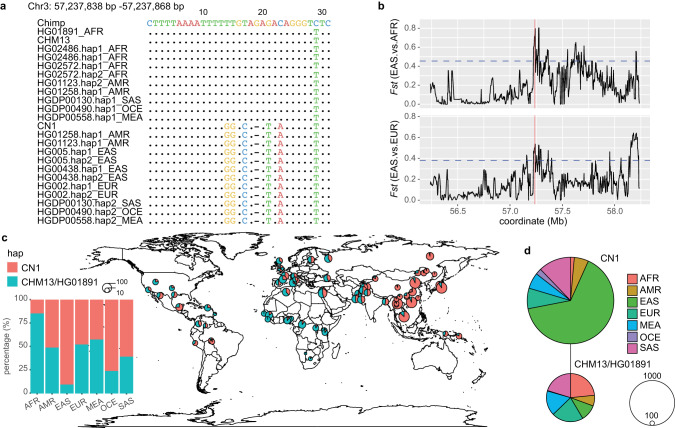
A CN1 accelerated region is under positive selection in Asian Population. **a** Alignment of chr3:57,237,838–57,237,868 sequences of CN1, chimpanzee and the haplotypes from different super-populations. Dots indicate identical base alignment. **b** *Fst* (EAS vs EUR and EAS vs AFR) scores around CN1 chr3:57,237,838–57,237,868 (red line) and its flanking region (window = 10 kb, step size = 2 kb). Blue dashed lines indicate the *Fst* cutoff with *P* < 0.05. **c** Global map shows the distribution of CN1 and CHM13/HG01891 haplotype frequency in 75 populations. The color and size of the circles represent the haplotype and the number of haplotypes, respectively. African, American, East Asian, European, Middle Eastern, Oceanian, and South Asian are denoted as AFR, AMR, EAS, EUR, MEA, OCE, and SAS, respectively. **d** Haplotype network of CN1 chr3:57,237,838–57,237,868. Most CN1-type haplotypes are found in EAS, while most CHM13/HG01891-type haplotypes are found in AFR. The color and size of the circles represent the super-population and the number of haplotypes, respectively.

We then examined whether these regions are under different selection constraints among the super-populations (AFR, EUR, and EAS). *Fst* analysis showed that 135 and 97 regions were under a considerable degree of differentiation between the EAS vs AFR and EAS vs EUR super-population pairs; 36 regions were common among the two datasets (Supplementary information, Table S30). Among these 36 regions, CN1 chr3:57,237,838–57,237,868, a 30 bp region containing five SNVs and one indel between CN1 and CHM13, showed the most significant accelerated signal (Fig. 4a, b). The super-population data from 1KGP and Human Genome Diversity Project (HGDP) revealed only two haplotypes in the global populations (i.e., the CN1 type and CHM13/HG01891 type) (Fig. 4c, d). In EAS, 91.48% of haplotypes were CN1 type, while in EUR and AFR, only 39.36% and 9.71% were CN1 type (Fig. 4c). Meanwhile, the orthologous sequence in the chimpanzee appeared more similar to the CHM13/HG01891 haplotype than the CN1 allele (Fig. 4a). All these observations suggest that this haplotype has experienced a specific accelerated process under a strong positive selection and has been maintained in the EAS super-population as the dominant one.

### Reference bias has a profound impact on rare SNP calling

**Fig. 5:**
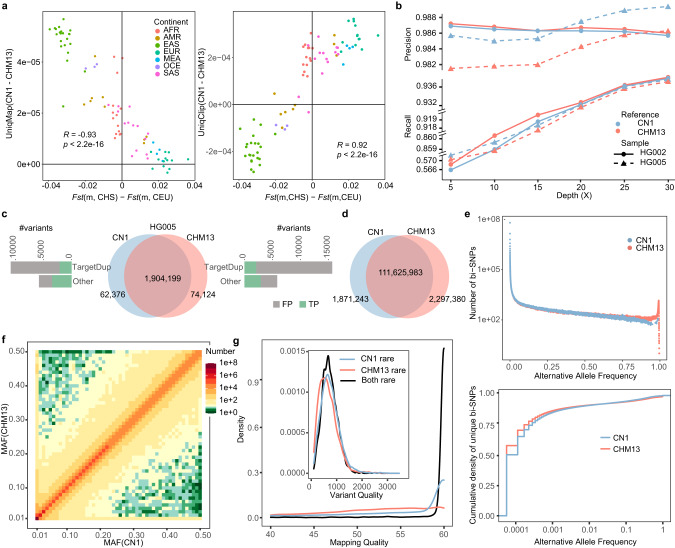
Reference bias using CN1 and CHM13 genomes for population genomic analyses. **a** The mapping statistics (left, unique mapping rate; right, unique clipping read rate) and their Pearson’s correlation with the difference in genetic distance (*Fst*) between the targeted population and the Southern Chinese (CHS) and Northern and Western European (CEU). Both graphs are plotted using *n* = 80 populations. **b** The performance of SNP calling in two benchmark samples from GIAB, a European individual (HG002) and an East Asian individual (HG005), using CN1 or CHM13 as a reference. Recall rates are displayed on a truncated *y-*axis. **c** Venn diagram shows the comparison of heterozygous SNVs called on CN1 and CHM13 genomes using ~30× HG005 sequencing data. Reference-dependent unique SNVs were compared with the GIAB benchmarked truth set, and classified as the true positives (TPs) and the false positives (FPs). TargetDup SNVs, caused by CNVs between the two references, are the major source of reference-dependent SNVs and introduce more FPs and comparable TPs in CHM13 than in CN1. **d** Venn diagram shows the comparison of bi-SNPs called from the 8869 Chinese cohort on CN1 and CHM13. **e** The alternative allele frequency distribution of bi-SNPs from 8869 Chinese genomes called on both CN1 and CHM13 genomes (upper). The alternative allele frequency distribution of the unique SNPs called on either CN1 or CHM13 genome (lower). **f** Heatmap shows the MAF of SNPs called on CN1 and CHM13. Most SNPs exhibit similar MAF in both references, while a few show distinct MAF between the two references (rare with CN1 but common with CHM13, and vice versa). More CN1 rare SNPs (upper left) are found than CHM13 rare SNPs (lower right). **g** Density distribution of mapping quality and variant quality of “Both rare” (rare SNPs called with both CHM13 and CN1), “CN1 rare” (rare with CN1 but common with CHM13), and “CHM13 rare” (rare with CHM13 but common with CN1) SNPs.

Each ethnic group has experienced its own admixture and adaptation history, accumulating many genomic variations that are different from other ethnic groups.^65^ Therefore, using the European descendent CHM13 or the mixed GRCh38 genome as the reference might have overlooked/mis-identified variations that are present in other ethnic groups. The CN1 individual was from Southern Chinese ancestry which was confirmed by principal component analysis (PCA) with 1KGP individuals (Supplementary information, Figs. S25, S26). Further local ancestry inference for both paternal and maternal haplotypes indicated that > 99% of each haplotype are of East Asian ancestry (Supplementary information, Table S32). Here, we used the two complete reference genomes (i.e., CN1 and CHM13) to assess the impact of reference bias on mapping and variant calling performed in different populations. We first mapped the publicly available raw sequencing reads obtained from 1500 samples of seven super-populations (African, American, East Asian, European, Middle Eastern, Oceanian, and South Asian denoted as AFR, AMR, EAS, EUR, MEA, OCE, and SAS, respectively), which are parts of 1KGP and HGDP, onto the CHM13 and CN1 genomes (Supplementary information, Table S33). We found a higher mapping rate for the EAS population on CN1 than on CHM13 (Supplementary information, Figs. S27–S30). For each population, we calculated the difference for the percent of reads uniquely mapped to CN1 and CHM13, and found that this difference decreased significantly (*R* = −0.93, *P* < 2e–16) along with the difference between the genetic distances of this population to Southern Chinese (CHS) and to Northern and Western European (CEU), the populations of which CN1 and CHM13 belongs to, respectively (Fig. 5a). Most of these uniquely mapped reads were observed in the CNV regions (see Materials and Methods) between CN1 and CHM13 genomes. HG005 dataset demonstrates an overall overestimation of copy number in CHM13 (Supplementary Notes), while fewer uniquely mapped reads on the copy gain (CPG) regions in CHM13 were observed compared to those on the CPG regions in CN1 (Supplementary information, Table S34), indicating the better performance of CN1 reference for East Asian. The structural variations between individual genome and the reference can cause clipping mapping (Supplementary Notes). We found that the difference in the number of clipping reads of the population unique to CN1 and CHM13 significantly increased (*R* = 0.92, *P* < 2e–16) along with the difference between the genetic distances of this population to CHS and to CEU (Fig. 5a). To summarize, these results suggest that a population closer to EAS generates more mapping reads and fewer clipping reads using CN1 as a reference. Besides, CN1 performs better on these two parameters for not only EAS, but also AMR and OCE (Fig. 5a).

We further assessed the impact of reference bias on SNP calling for personal genomes using the genomic data of the European descendent HG002 and the Chinese descendent HG005 from an authorized human genome benchmark, the Genome in a Bottle (GIAB).^66^ The heterozygous SNP variants called for HG002 exhibited higher precision and recall rates on the CHM13 reference than on the CN1 reference, while the opposite was observed for those of HG005 (Fig. 5b; Supplementary information, Table S35). We found that ~3% of SNPs were uniquely called in each reference (Fig. 5c; Supplementary information, Fig. S31). Further comparison of these reference-dependent unique SNPs with the GIAB benchmarked truth set showed that HG005 had a higher precision rate on CN1 than on CHM13, while HG002 had a higher precision rate on CHM13 than on CN1 (Supplementary information, Table S36). To further determine the source of these unique SNPs, we extracted sequencing reads contributing to them and located their mapping coordinates in the other reference genome (Supplementary Notes). We found that most of these reads were mapped to multiple positions in the other reference (Fig. 5c; Supplementary information, Fig. S31, Table S37), probably due to the CNVs between the two references. Indeed, we found that ~30% of these unique SNPs were located in the CN1-CHM13 CNV regions, which only occupied < 1% of the entire genome, indicating the significant impact of CNVs on SNP calling.

To further investigate how reference usage affects SNP calling on a large cohort of samples, we collected and aligned the high-coverage genomes (~30×) of 8869 Chinese individuals (Supplementary Notes) onto the CN1 and CHM13 reference genomes. We detected 113,497,226 bi-allelic SNPs (bi-SNPs) with CN1 reference and 113,923,363 SNPs with CHM13 reference (Fig. 5d; Supplementary information, Table S38). The allele frequency spectrum showed more SNPs with fixed or nearly fixed alleles (allele frequency > 0.9) using CHM13 than using CN1 as a reference, suggesting that CN1 represents the major allele of the Chinese population better than CHM13 (Fig. 5e). We detected 1,871,243 unique SNPs using CN1 as a reference and 2,297,380 unique ones using CHM13 as reference (Supplementary information, Table S38). Notably, 91.86% and 92.22% of the CHM13-unique and CN1-unique SNPs were rare (minor allele frequency (MAF) < 0.01) (Fig. 5e), implying their susceptibility to reference bias. We also detected a significant accumulation of these unique SNPs in the centromere (50%) and the structural variations (10%) between the two reference genomes (Supplementary information, Fig. S32). Surprisingly, among those bi-SNPs called in both references, we found that 15,227 called as rare SNPs (MAF < 0.01) in CN1 were called as common SNPs (MAF > 0.05) in CHM13 (Fig. 5f). In contrast, only 2928 rare SNPs called using CHM13 were detected as common SNPs using CN1 (Fig. 5f). To assess the quality of SNPs with inconsistent frequency using different references, we randomly extracted 2500 rare SNPs detected with both references (“Both rare”) and used them as control. We found that both the mapping quality and the SNP quality of the CN1-specific rare SNPs were more similar to the control and significantly higher than those of the CHM13-specific rare SNPs (two-sided *t-*test, *P* < 2.22e–16, Fig. 5g; Supplementary information, Fig. S33). These observations suggest that the rare SNPs called in the Chinese population using CN1 as a reference are more accurate than using CHM13 as a reference.

### CN1 reference assists to detect novel introgression from ancient hominin genomes

Studies have suggested that the East Asian population has encountered more waves of Denisovan admixture than the European population^67^ and carried a higher proportion of Neanderthal ancestral sequences.^68,69^ Since the ancient DNA samples are highly fragmented and are divergent from the modern population, the palaeogenomic analysis is particularly vulnerable to reference bias,^11^ leading to a failure in detecting the potential introgression regions unique to the East Asian populations when using the mixed GRCh38 genome or CHM13 as reference. The East Asian complete reference genome offers us an opportunity to reduce the bias and detect the East Asian-specific archaic introgression. We first mapped the two high-coverage archaic genomes (Altai Neanderthal and Denisovan)^68,70^ against the CN1, CHM13, and GRCh38. We showed that using GRCh38 as a reference, which includes many collapsed duplication regions, identified more artificial heterozygous SNPs in ancient genomes. Using the two complete genomes as references could efficiently reduce such artifacts of the heterozygous SNPs with unusual mapping depth in the ancient genomes (Supplementary information, Fig. S34). Further, comparing the mapping coverages of the ancient genomes to the two complete references, we discovered 8758 (~4.29 Mb in length) and 10,199 (~5.27 Mb) regions in CN1 evenly mapped by the high-coverage reads (> 1/2 whole-genome mapping depth) from Neanderthal and Denisovan genomes, respectively, which were unmapped against the CHM13 genome (Supplementary information, Table S39), indicating that the East Asian genomes may have experienced unique introgression events. Meanwhile, a total of 9388 (~5.75 Mb) and 10,387 (6.76 Mb) specific mapping regions (SMRs) were identified in CHM13, for the Neanderthal and Denisovan genomes, respectively. We detected even more unique CN1 SMRs for Denisovan than those for the Neanderthal genome (1.49 Mb vs 0.73 Mb). At least 2763 (2.08 Mb, 48.4% in total length) and 3765 (2.84 Mb, 54.6%) CN1 SMRs in the Neanderthal and Denisovan genomes were due to the SVs between CN1 and CHM13.

We then calculated the modified *D*-statistics (*f*_d_ in ABBA-BABA test) to detect the putative introgression regions (pIRs) in different modern human populations from Neanderthal and Denisovan genomes using CN1 and CHM13 as the reference genomes.^68,71^ In the quartet “((Bantu Kenya, Han), Neanderthal/Denisovan, and Chimpanzee)”, we identified 114.78 Mb and 82.65 Mb genomic regions with putative introgression signals (*f*_d_ > 0.35) from Neanderthal and Denisovan, respectively (Supplementary information, Table S40). The total lengths of these pIRs detected using CN1 as a reference were a bit longer than those of pIRs detected with CHM13 as a reference (112.18 Mb and 80.65 Mb for Neanderthal and Denisovan, respectively). Interestingly, we detected more pIRs in Oceania or Melanesian population (Bougainville) using CN1 as the reference than using CHM13 (Supplementary information, Fig. S35). Further comparison of pIRs detected using the two references identified over 9.09 Mb novel Neanderthal regions and 7.49 Mb novel Denisovan regions in the modern Han genomes using CN1 as the reference, of which, 5.80 Mb and 4.21 Mb were specifically introgressed to East Asian (Han) but not European population (French) from Neanderthal and Denisovan, respectively.

**Fig. 6:**
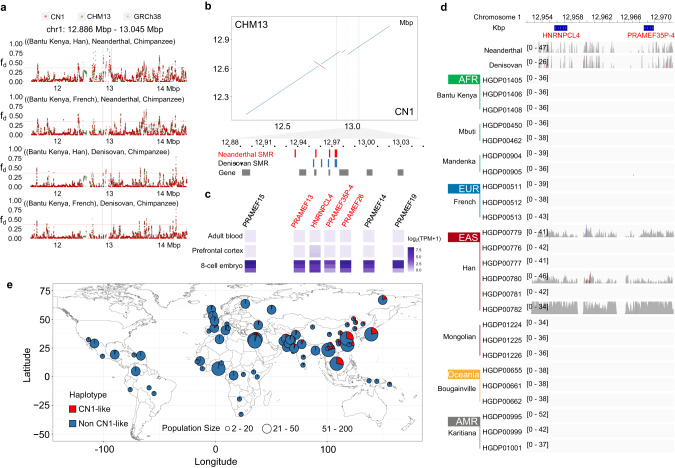
A pIR from archaic genomes in the East Asian population identified using CN1 as a reference. **a** Distribution of modified *D*-statistics *f*_d_ values along chromosome 1 in ABBA-BABA test. Four comparisons were set in topology ((P1, P2), P3, Outgroup), where outgroup was Chimpanzee, P1 was Bantu Kenya, P2 was Han (EAS) or French (EUR), and P3 was Neanderthal or Denisovan genome. Three reference genomes were used, and the window coordinates in CHM13 and GRCh38 were converted into those in CN1 by LiftOver. The interval between the two vertical lines highlights the pIR in Han. The red horizontal line represents the empirical cutoff (*f*_d_ = 0.35). **b** Local synteny between CN1 and CHM13. Red vertical lines indicate the genomic positions of the annotated genes in CN1. SMRs for Neanderthal and Denisovan are marked in red and blue, respectively. **c** Expression profiling of four newly annotated genes (red) and flanking genes located in the pIR in CN1. **d** Mapping depths of different modern human population genomes onto CN1. Each of the depth tracks ranges from zero to the whole-genome depth, respectively. **e** Global distribution of CN1-like haplotype frequency in 75 populations.

Totally 1.63 Mb and 1.39 Mb of pIRs from Neanderthal and Denisovan in the Han population using CN1 as a reference failed in conversion by coordinates to CHM13 directly by LiftOver. Among them, one large pIR from 12.886 Mb to 13.045 Mb on CN1 chr1 for Han was located within a cluster of PRAME family members, while no introgression signal was detected in this region using CHM13 or GRCh38 as the reference (Fig. 6a). This large pIR was also not detected in the French population using any of the three genomes as reference. Local synteny showed an 86 kb insertion (from 12.903 Mb to 12.989 Mb) in CN1 compared to CHM13, containing at least three genes (*PRAMEF13*, *HNRNPCL4*, and *PRAMEF26*) and one pseudo-gene (*PRAMEF35P-4*) (Fig. 6b). Several CN1-specific mapping regions appeared overlapping in this region for both Neanderthal and Denisovan genomes. Analysis of the RNA-seq data from Chinese samples revealed expression of these four genes during embryo development (Fig. 6c). Consistent with the ABBA-BABA tests, a certain number of reads from the two ancient genomes and Han genomes were mapped against this region, but no mapping was found for AFR, EUR, Oceania, and AMR populations (Fig. 6d). A comparison of CN1 with the phased assemblies in HPRC^47^ showed that none of the AFR genomes were CN1-like, but the maternal assembly of HG02080 from Vietnam (EAS) was highly syntenic with CN1 (Supplementary information, Fig. S36). Finally, using a panel of global modern human genomes, we genotyped the presence-and-absence variation of this CN1-like insertion in 75 populations. The CN1-like insertions were broadly distributed in EAS populations with a mean frequency of 25.4%, while none was observed in the African populations, which provided an indispensable piece of evidence supporting archaic introgression into EAS genomes (Fig. 6e).

## Discussion

Recent advances in the pangenome studies have suggested that a graph-based pangenome with whole-genome sequences of individuals from diverse populations can improve the accuracy of variant calling and reduce the reference bias.^72–74^ However, in practical usage, reference genome coordinates are essential for gene and variant annotations, which is complicated to incorporate with graph-based pan-genomic data. Moreover, it is difficult to integrate the visualization of the genomic graph in current genome browsers designed to access linear tracks of genomic data. Therefore, the pangenome graph has to maintain backward compatibility with the linear references for effective interpretation during genetic analysis under the current genomic database framework.^75^ To overcome these difficulties, we aimed to generate a well-annotated T2T genome assembly as a linear reference for the local population. This local reference genome will supplement the pangenome in capturing the full genomic variations and assisting gene-disease association studies in the targeted population. This new reference genome will also be useful to integrate the genomic variations that have been separately reported in several large-scale Chinese genome initiations such as the HuaBiao project,^76^ the NyuWa Genome resource,^77^ the ChinaMAP project^78^ and the Chinese Pangenome Consortium (CPC) project.^18^ Though it will come at some cost on computation and communication in simultaneous utilization of multiple reference genomes, a routine alignment and projection of the up-to-date annotations between the local reference and commonly used reference, such as GRCh38, in a timely way can largely reduce such cost.

The release of the T2T genome assembly of CHM13 in 2022 fully completes the human genome, which enables the comprehensive detection of genomic variants and marks a new era in human genomic research.^58,75,79,80^ However, achieving a T2T genome still requires a substantial amount of manual curation by a group of experts. Meanwhile, a routine pipeline to produce a complete personal genome with two haploid genomes fully phased into the T2T level has yet to be established.^26^ Our current study presents the first truly complete human diploid genome and demonstrates the feasibility of producing a complete personal genome in a T2T de novo way. This complete genome from a Han Chinese allowed us to assess the genetic diversity at the whole-genome level between the two haploids of the same individual and between the individual genomes. We also characterized the SVs on centromeric regions, rDNA, and heterochromatic regions of the Y chromosome, which were not covered in previous comparative studies based on draft human genomes. We showed that the variations in these regions reflect the copy number difference in the repeats and the structural composition of the repeat units. Comparing the two T2T genomes also revealed many novel SVs, indicating that many variations remain unexplored at the population level. For example, we used PacBio HiFi reads of 5 EAS individuals (HG00438, HG00621, HG00673, HG02080, HG005) from HPRC to call SVs using CN1 as a reference to show the efficacy of the CN1 genome. On average, each individual can generate 17,246 SVs, including 12,124 heterozygous SVs and 5122 homozygous SVs (Supplementary information, Table S41). Each sample harbored 3267 unique SVs on average (Supplementary information, Fig. S37). These results highlight the importance of expanding the available human T2T genomes from more diverse ethnic groups. The Human Pangenome Reference Consortium and the Chinese Pangenome Consortium have started building high-quality haploid genomes from diverse samples.^18,26,73^ Besides, preliminary studies based on these datasets have boosted the discovery of novel variants among populations.^47,57^ We could anticipate that the human pangenome at T2T level from more diverse groups will soon provide us a more complete overview of global human genomic diversity in the near future.

The new T2T CN1 genome also enabled a comprehensive evaluation of the reference bias in population genomic studies. Comparing the two performance parameters in resequencing analyses, the mapping and variant calling rate, using the CN1 and CHM13 as references with a wide range of population data, we showed that CN1 outperforms CHM13 on population genomic data from EAS, AMR, and OCE populations. This observation is consistent with the migratory history of these two populations from the East Asian population.^81^ Besides, relatively higher mapping rates and lower clipping rates of reads from these populations were observed on the CN1 genome, primarily attributed to the structural variations between East Asian and European genomic background. Owing to the different mapping performances, the SNP calling on the same individual and population data showed that more SNPs were reference-dependent, indicating the impact of reference bias on SV detection. Notably, most of these reference-dependent SNPs in the population data were rare SNPs. These rare variants are known to play significant roles in inherited rare diseases and explain a substantial portion of the missing heritability of complex diseases^82–84^ and are of great interest for large cohort studies.^85–87^ Our study suggests that an important caution is needed in selecting a reference for population genetic studies on non-European populations, particularly in detecting the rare SNPs. We finally propose that the well-annotated CN1 genome could be an alternative reference in addition to the current reference for future cohort studies in the East Asian population.

## Materials and methods

### Sample preparation and sequencing

A family consisting of seven individuals from three generations from Hubei, China, was recruited, and matched the following criteria: (1) five upward tracing generations were Han Chinese; (2) each individual has a normal phenotype and no genetic diseases; (3) the third generation is a male. The whole blood was collected from a family residing in southern China, consisting of the mother, father, and the son CN1, under the approval number IACUC-RE-2021-10-003 from the Ethics Committee of the Kunming Institute of Zoology, Chinese Academy of Sciences. All participates provided informed consent for sample collection. High-molecular-weight genomic DNA was isolated using the CTAB method, followed by purification with the QIAGEN Genomic kit (Cat#13343, QIAGEN) according to the manufacturer’s protocol for sequencing. The quality of the extracted DNA samples was assessed by analyzing degradation and contamination on 1% agarose gels. The purity of DNA was evaluated using NanoDrop^TM^ One UV-Vis spectrophotometer (Thermo Fisher Scientific, USA), with an OD260/280 ratio ranging from 1.8 to 2.0 and OD260/230 ratio between 2.0 and 2.2. The concentration of DNA was further quantified using the Qubit 4.0 Fluorometer (Invitrogen, USA).

#### PacBio HiFi reads

SMRTbell target size libraries were constructed for sequencing according to PacBio’s standard protocol (Pacific Biosciences, CA, USA) using 15 kb preparation solutions. Sequencing was performed on a PacBio Sequel II instrument with Sequencing Primer V2 and Sequel II Binding Kit 2.0 in GrandOmics.

#### ONT reads

Approximately 8–10 μg of genomic DNA was used to construct an ultra-long Nanopore library using the Ligation sequencing 1D kit (SQK-LSK109, Oxford Nanopore Technologies, UK) following the manufacturer’s instructions after size selection (> 50 kb) using SageHLS HMW library system (Sage Science, USA). About 800 ng DNA libraries were obtained and sequenced on the Promethion (Oxford Nanopore Technologies, UK) at the Genome Center of GrandOmics (Wuhan, China).

#### Genome sequencing

Two different whole-genome sequencing (WGS) datasets were generated. The first one was generated from paired-end 150 bp libraries on MGISEQ T7, with 97× coverage for CN1, 118× coverage for the mother, and 96× coverage for the father. The second one was generated from PCR-free paired-end 150 bp TruSeq (LT) libraries on Illumina NovaSeq 6000, with 108× coverage for CN1, 101× coverage for the mother, and 115× coverage for the father.

#### Hi-C linked reads

Hi-C libraries were constructed using genomic DNA from the white blood cells and sequenced on the MGISEQ T7 platform. A total of 1,194,260,771 read pairs (paired-end 150 bp) were generated.

#### Optical mapping

Optical mapping was performed using Bionano’s next-generation mapping technique based on the Bionano DLE1 data from GrandOmics, including 813.91 Gb (molecules of > 150 kb) and N50 reads of 342 kb from molecules of > 150 kb.

#### mRNA sequencing

White blood cells were isolated from whole blood using the centrifugation method. Total mRNA was extracted from the cells using the TRIzol method. Each mRNA sample was sequenced using the MGISEQ T7 platform to generate ~25 Gb of data.

### Genome assembling, polishing, and annotation

The initial assemblies were constructed with verkko (v1.0)^28^ and hifiasm (v0.16.1)^27^ in the trio mode. Additional assemblies were also constructed using Flye (v2.9-b1774)^31^ with canu-based binned ONT reads. The draft CN1 maternal and paternal genomes were scaffolded mainly based on verkko and hifiasm assemblies (Supplementary Notes). The gaps were initially filled using TGS-GapCloser (v1.2.1)^30^ based on the hifiasm and Flye assemblies and binned ONT reads. To fill the remaining gaps, the 5 kb of upstream and downstream regions were extracted and aligned to the binned ONT reads to identify any alignment. These alignments were further visualized and manually examined using LINKVIEW (https://github.com/YangJianshun/LINKVIEW). Only alignments with proper size and non-conflict orientation were used to fill the gaps. Moreover, the CN1 genome was polished mainly following the T2T polishing pipeline.^24,88^ Briefly, binned ONT reads and all HiFi reads were mapped to each CN1 haploid genome using winnowmap2 (v2.03),^89^ and primary alignments were kept. BGISEQ short reads were mapped to CN1 genome using BWA MEM (v0.7.17-r1188)^90^ and duplications were marked with “bamsormadup” from biobambam2 (v2.0.183).^91^ Small variants were called using the “hybrid” mode in DeepVariant (v1.4.0)^32^ based on the combined HiFi and BGISEQ alignment, and SNVs in the binned ONT alignment were identified using PEPPERDeepVariant (r0.8).^33^ These variants were combined mainly based on variants called from binned ONT alignment (Supplementary Notes). SVs were called with sniffles2 (v2.0.7)^92^ and manually examined using a modified version of bamsnap^93^ (https://github.com/zy041225/bamsnap). These variants were then merged and used to polish the genome using merfin (v1.1)^94^ and bcftools (v1.16).^95^ A total of five rounds of polishing were performed. Detailed assembling and polishing information are available in Supplementary Notes.

Repeats in each genome were identified using RepeatMasker (v4.1.2-p1)^96^ based on the Dfam (v3.6) repeat library. Gene annotation was performed by employing liftoff (v1.6.3)^97^ and LiftOver (v438) to project the GRCh38.p14 RefSeq v110 reference annotation onto the assembly as the main source. Chinese RNA-seq transcriptome data, human uniprot_sprot (release-2022_05) protein data, BRAKER (v2.1.6)^98^ annotation, and Augustus (v3.4.0)^99^ annotation were integrated into the gene dataset using EVidenceModeler (v1.1.1).^100^ The obtained annotation was further complemented with tRNA annotation using tRNAscan-SE (v2.0)^101^ and other ncRNA annotation using Rfam (v14.9).^102^ Details are shown in Supplementary Notes.

Two haplotype genomes were compared using minimap2 (2.24-r1122w)^103^ and paftools (2.24-r1122). All variants were first generated to visualize the heterozygosity between two haplotype genomes. Then, 500 kb non-overlapping windows were slid to calculate the count *h* of SVs. We set the threshold as *h* = 2 SVs per 500 kb, and assigned the window as “hete” (≥ 2) or “homo” (< 2) type, and the plot as the bubble or single path (grey), respectively (https://github.com/T2T-CN1/CN1/tree/main/heterozygosity). Additionally, the heterozygosity rate was mapped to sequential color for each pair of bubbles. Finally, a GFA file was generated using this information and visualized using Bandage (v0.8.1).^104^

### Centromere analysis

The centromere satellites were annotated following the T2T pipeline to produce the cenSat annotation track. Briefly, the αSat was annotated using hmmer (v3.3.2)^105^ with the profile at https://github.com/fedorrik/hmm/blob/main/AS-HORs-hmmer3.0-170921.hmm, and the output was converted to the BED format via the script at https://github.com/enigene/hmmertblout2bed. The regions covered by HORs or divergent HOR monomers were annotated as “hor” or “dhor”, respectively, and the remaining αSat arrays were annotated as “mon”. The HSat2 and HSat3 were identified using the script at https://github.com/altemose/chm13_hsat, and the remaining satellites were identified based on database Dfam (v3.6) using RepeatMasker with parameters “-species Homo sapiens -s”. The presence of HSat2 in CN1 chr15, the absence of inactive HOR, and the presence of HSat2 in CN1 chr14 were confirmed via IGV (2.14.1).^106^ All HOR SVs in the active HOR of CN1 assembly were identified using the script at https://github.com/fedorrik/stv, and the intra-monomer divergence of the active HOR array was calculated following the p-distance formula by deleting all gaps, as previously reported^39^:

$${divergence}\,={mismatched}\,{bases}/({aligned}\,{bases}-{alignment}\,{gaps})\,$$

The method of novel monomer identification is shown in Supplementary Notes.

### Methylation analysis

HiFi-binned ultra-long reads were mapped to the CN1 maternal and paternal genomes using winnowmap. Only primary alignments > 50 kb were retained for centromeric region analyses. The Nanopolish (v0.13.2) pipeline described in a previous report^107^ was employed to estimate the methylation frequency in each CpG site. Log-likelihood values of 1.5 and −1.5 were used for a high probability of methylation and unmethylation, respectively. Information from all covered ONT reads was combined to obtain the methylation level of each site. CpG sites covered by fewer than 5 ONT reads were filtered out. The average methylation frequency was calculated with neighboring three 10-kb bins. The CDRs of each chromosome for CN1 were manually examined and annotated to satisfy the following criteria: (1) the methylation frequencies of consecutive 10-kb bin were considerably lower than the average methylation of the whole HOR region, and (2) the conspicuous hypomethylated region was located in the active HOR. CHM13 methylation data were obtained from https://s3-us-west-2.amazonaws.com/human-pangenomics/index.html?prefix=T2T/CHM13/assemblies/annotation/regulation/. CHM13 CDRs were obtained from a previous report^41^ except for chr21. Because the CHM13 CDR coordinate of this chromosome given in their Supplementary Tables^41^ does not match its methylation data, the methylation data were manually examined to obtain its CDR coordinate. The average methylation level in 10 kb windows was calculated, and the values were visualized in the CN1 diploid genome (or CHM13) using karyoploteR (v1.21.0).^108^ The sequences of CHM13 CDRs were first blasted using blastn (v2.13)^109^ under parameters “-outfmt 6 -dust no -soft_masking false -max_target_seqs 1000000 -ungapped” and the alignment dotplots were visualized using the modified version of dotPlotly available at: https://github.com/zy041225/dotPlotly/blob/master/blastM8DotPlotly.1vs1.addHOR.v1.1.R. The best identity of each CDR pair was extracted using Genodps (https://github.com/rsharris/genodsp) from blastn alignment and identity of each CDR pair was calculated and weighted by alignment length.

### Genomic comparison between CN1 and CHM13

After masking the centromeric and heterochromatic regions into Ns, each chromosome of CN1 maternal (or paternal) genome was aligned to the corresponding chromosome of CHM13 using nucmer (v4.0.0rc1)^110^ with parameter set “--maxmatch -t 12 -l 100 -c 500”. Delta results were later filtered using delta-filter with parameter “-m -i 90 -l 100”, and SVs were identified using SyRi (v1.6.3).^111^ Only SVs > 50 bp were retained. These SVs, along with the flanking regions, were then manually examined using LINKVIEW to filter out false positive SVs introduced due to problematic alignment. The SVs of the combined CN1 genome were generated by combining the SVs detected between CHM13 and CN1.mat genome and between CHM13 and CN1.pat genomes.

The coordinates of SVs in two SV datasets, HGSVC phase II^46^ and HPRC,^47^ were compared to identify CN1-specific novel SVs. Given that the HPRC dataset and CN1 SVs were generated based on CHM13, HGSVC database was lifted over to CHM13. The other two types of SVs (insertion and deletion) were compared because translocation, inversion and duplication were missing in the HGSVC and HPRC. All SVs were merged using SURVIVOR (v1.0.7)^112^ “merge” function with parameters “1000 1 1 -1 -1 50”, and 6641 novel SVs (length ≥ 50 bp) were obtained in CN1.

We further classified CN1 genome into different types of regions based on the SVs. Complex SVs such as inversion, translocation and inverted translocation were taken and classified into the “Other SV” category. Regions with CNVs between the two genomes are classified into “CPG in CN1” and “CPL in CN1” based on the SVs as well. Specifically, if an SV was categorized as “CPG” or “INS” in CN1 as compared to CHM13, or the SV was HDR or TDM and the CN1 region was longer than the CHM13 region, the region would be classified as “CPG in CN1”. Likewise, if an SV was categorized as “CPL” and “DEL” in CN1 compared to CHM13, or the SV was HDR or TDM and the CN1 region was shorter than the CHM13 region, the region would be classified as “CPL in CN1”. Centromere regions were based on the annotation in the two genomes.

### Detecting novel sequences in CN1

The comparison with CHM13 revealed that most chromosomes in CN1 and CHM13 shared the same sequence components, except for HOR SVs and novel monomers found in chr17 and chr21. Because it is unlikely to detect novel sequences in the centromere due to the highly repetitive nature of these regions (even the newly detected monomers in chr21 has > 90% identity with the previous model) and it takes extremely high time cost for genome alignment, both the centromeric and heterochromatic regions in CN1 and CHM13 were first masked by replacing them with Ns. Then CN1 was aligned to CHM13 using nucmer with parameters “-t 12 -l 100 -c 500”, and all fragments mapped to the reference with ≥ 80% identity were removed. The unmapped fragments of > 100 bp were further aligned to the reference using blastn with a parameter setting of “-evalue 1e-5 -num_threads 24 -max_target_seqs 100 -outfmt '6 qseqid qlen sseqid slen pident length mismatch gapopen qstart qend sstart send evalue bitscore'”, and any hits that had an alignment identity of ≥ 80% were excluded. Further, contamination filtering, sequence clustering, and repeat removal were conducted using scripts from the AF-NS tool (https://github.com/HKU-BAL/AF-NS).^53^ Specifically, the longest sequence from one cluster was retained as a representative sequence if they aligned with each other with > 80% coverage. Finally, we utilized RepeatMasker to annotate the candidate novel sequences and removed sequences if 80% of the sequence was low-complexity or simple repeats. To avoid aligner bias, extra filtering was conducted by mapping potential novel sequences to CHM13 using BLAT (v37x1).^113^ After that, the final set of the novel sequence was generated and mapped to CN1 using blastn to find all placements. We searched the CN1 novel sequences among EAS samples from HPRC (HG00438, HG00621, HG00673, HG02080, HG005), and seven primates (macaque, marmoset, gorilla, bonobo, chimpanzee, hamadryas baboon, pileated gibbon) using minimap2 with coverage > 90% and identity > 90% as thresholds.

### Y chromosome analysis

The subregions on CN1-Y and hg38-Y were classified based on the LiftOver results from the CHM13v2.0 annotation (https://s3-us-west-2.amazonaws.com/human-pangenomics/T2T/CHM13/assemblies/annotation/chm13v2.0_chrXY_sequence_class_v1.bed). The LiftOver chain from CHM13v2.0 to hg38 was generated using the nf-LO pipeline as described in https://s3-us-west-2.amazonaws.com/human-pangenomics/T2T/CHM13/assemblies/chain/v1_nflo/v1_nflo_description.html. PAR boundary was confirmed based on the MUMMER alignment between chrX and chrY. The centromere boundary was confirmed according to the pipeline described in a previous study.^57^ Palindrome boundaries were identified following the method described in https://github.com/arangrhie/T2T-HG002Y/tree/main/amplicons_and_palindromes. HSat in CN1 heterochromatin region was annotated as described in a previous study.^39^ HSat in HG002-Y was obtained from https://github.com/altemose/HSatReview/blob/main/Input_Files/chm13v2.0_DistinctArrays_INFO_coords.bed. The whole Y chromosome dotplot between CN1 and hg38 (or HG002) was generated using lastz alignment following https://github.com/arangrhie/T2T-HG002Y/tree/main/alignments/lastz. SVs between the CN1-Y and HG002-Y assemblies were called using SyRi based on the minimap2 alignment generated with parameter set “-ax asm20 --eqx” and further manually confirmed with the dotplot.

To calculate the average identity in each subregion, chrY was split into 27 subregions and subjected to one-to-one MUMMER alignment with parameter set “--maxmatch -l 100”. The best identity and the identity was calculated and weighted by alignment length. DYZ19 alignment between CN1 and HG002 was generated using blastn with parameter set “-dust no -soft_masking false -max_target_seqs 1000000 -ungapped”. Intrachromosomal alignment was visualized using StainedGlass (v0.5),^114^ with 1 kb window size for DYZ19 and 5 kb window size for the heterochromatic region. Subregion alignments between assemblies were visualized based on dotplots using the modified dotPlotly available at https://github.com/zy041225/dotPlotly/blob/master/mummerCoordsDotPlotly.1vs1.R.

### SD analysis

SDs were detected using SEDEF (v1.1, commit g5acd139).^115^ However, since SDs between assemblies cannot be directly lifted over, minimap2 was employed to first lift over their flanking regions. Non-syntenic SDs were detected by considering the length of LiftOver flanking regions (> 3 times or < 1/3 of the SD length before lifting over) and the mapping quality of the LiftOver flanking regions (equal to 0). The intersect gene models and non-syntenic SDs were intersected using bedtools (v2.30.0).^116^

To determine whether CN1 was a more suitable reference genome for the Chinese population than CHM13, the copy number of protein-coding genes in 301 Chinese samples from the 1KGP and the two reference genomes (CHM13 and CN1) were estimated using fastCN (commit f97eb25).^117^ CN1 was regarded as a better representative reference genome for the Chinese population if the copy number of a given gene in these samples was significantly closer to that in CN1 than in CHM13. The significant difference between any two datasets was evaluated using the two-sided *t*-test. The syntenic relationship of *ZDHHC11* gene cluster between CN1 and CHM13 was examined using minimiro (commit 8a77b25, https://github.com/mrvollger/minimiro). In addition, the CNV of *ZDHHC11* was examined in various populations in 317 human samples from the Simons Genome Diversity Project^61^ using fastCN.

### CN1 accelerated region analysis

Whole-genome alignment was performed to obtain multiple sequence alignment by mapping CHM13, and “HG01891” (sequences of autosome and X chromosome from HG01891 and Y chromosome from HG02486), to CN1, with parameter set “--hspthresh = 36400 --format = axt”. The AXT results were first converted to CHAIN files using axtChain with “-minScore = 5000” and then to NET format. The pairwise reciprocal best alignments were obtained following the guide at http://genomewiki.ucsc.edu/index.php/HowTo:_Syntenic_Net_or_Reciprocal_Best. The resulting CN1-CHM13 and CN1-HG01891 MAF blocks were then combined using multiz (v11.2).^118^ Only alignments involving all three genomes were retained for downstream analysis.

To identify CN1 accelerated regions, phyloFit in the PHAST package (v1.5)^119^ was run with the topology “(HG01891, (CHM13, CN1))” to estimate the neutral (“non-conserved”) model based on four-fold degenerate sites. The input MAF was then split into 20 bp windows with 10 bp overlap. Moreover, phyloP with “--method LRT --mode CONACC” was run using the non-conserved model as the input to identify candidate CN1 accelerated regions from these windows. The identified windows with significantly accelerated signals (*P* < 0.01 in the likelihood ratio test (LRT)) were merged and subjected to the second run using phyloP to filter out potential false positives. By manually examining the alignment, we found that some of the alignments can be improved, and thus we realigned sequences of the candidate CN1 accelerated regions with MAFFT (v7.505),^120^ and further subjected them to phyloP run for the third time. The resulting regions with significantly accelerated signals (LRT, *P* < 0.01) were considered as CN1 accelerated regions. Based on the MAF alignment, SNVs between CN1 and CHM13 and between CN1 and HG01891 were extracted from the CN1 accelerated regions and the other regions. SNV density in every 20 bp window was calculated, and the differences in the SNV density of every 20 bp window between CN1 accelerated regions and the other regions were examined using the Wilcoxon rank sum test. To lower the false positive introduced by assembly error, the HiFi and ONT read alignment at SNVs between CN1 and the other two genomes was extracted and examined using a custom python script. The regions containing false positive SNVs were considered false positive CN1 accelerated regions and thus removed.

The 1KGP and HGDP data (see below “Resequencing data analysis”) were used to determine whether the CN1 accelerated regions were also under the differentiation between populations. Therefore, Jointcall VCF was taken, and *Fst* of every SNP between EAS and EUR (or AFR) was calculated using vcftools (v0.1.13).^121^ An empirical *P*-value obtained by comparing the *Fst* of a SNP and the *Fst* distribution of intergenic SNPs was calculated using the same method described in a previous study.^122^ Every CN1 accelerated region was deemed to have > 50% SNVs with a *P* < 0.05.

### Resequencing data analysis

#### Read mapping and variant calling

The mapping performance for short-read WGS data between CN1 (v0.6) and CHM13 (v2.0) was compared based on 1KGP and HGDP datasets. A subset of the data (~700 individuals from 1KGP and ~800 individuals from HGDP) were used to represent diverse populations (Supplementary information, Table S33). CRAM data from 1KGP were transformed into FASTQ format using samtools (v1.10)^123^ and bedtools before mapping. The paired-end reads were processed following the Genome Analysis Toolkit (GATK) best practice guidance using ZTRON (https://en.mgi-tech.com/products/software_info/3) and ZBOLT (MegaBOLT v2.3.0.0, https://en.mgi-tech.com/products/software_info/6). Public variant datasets for GRCh38 (obtained from gs://genomics-public-data/resources/broad/hg38/v0/1000G_phase1.snps.high_confidence.hg38.vcf.gz, gs://genomics-public-data/resources/broad/hg38/v0/hapmap_3.3.hg38.vcf.gz, gs://genomics-public-data/resources/broad/hg38/v0/Mills_and_1000G_gold_standard.indels.hg38.vcf.gz, gs://genomics-public-data/resources/broad/hg38/v0/1000G_omni2.5.hg38.vcf.gz) and dbSNP build 151 were projected on CN1 coordinates to facilitate the variant calling and filtering process (BQSR + VQSR). Variants in these 1500 samples collected using MegaBOLT were merged by jointcall and filtered using VQSR. All mapping and calling statistics were collected using in-house scripts.

#### Mapping performance and its correlation with genetic distance

For each sample *n* in population *m* with the sample size *N*, the percent of reads that were uniquely mapped to CN1 was denoted as UniqMap_*n*,*m*,CN1_ and that were uniquely mapped to CHM13 was denoted as UniqMap_*n*,*m*,CHM13_. For each population, the average UniqMap_*m*,CN1_ and UniqMap_*m*,CHM13_ was calculated as ∑_n_UniqMap_*n*,*m*,CN1_/*N* and ∑_n_UniqMap_*n,m*,CHM13_/*N*, respectively.

The genetic distance between each pair of 80 populations from 1KGP and HGDP was calculated using jointcall and VQSR-pass variants. 1/1000 variants were randomly sampled and further filtered with MAF > 0.01. The remaining variants were used to calculate *Fst* between each population pair using adegenet (v2.1.8) R package.^124^ Since CN1 is a sample of Chinese Han South, and CHM13 is of mostly European ancestry, the *Fst* of each population *m* was extracted from CHS and CEU and denoted as *Fst*_*m*,CHS_ and *Fst*_*m*,CEU_, respectively. Finally, the correlation of mapping performance and genetic distance was plotted using *Fst*_*m*,CHS_ – *Fst*_*m*,CEU_ as the *x* coordinate and UniqMap_*m*,CHM13_ – UniqMap_*m*,CN1_ as the *y* coordinate. The clipping reads were counted using the CIGAR value, and the percentage of uniquely clipped reads was defined as UniqClip. The same calculation process for UniqMap was applied to UniqClip.

#### PCA and local ancestry inference for CN1

Variants of CN1 and 1KGP samples called on CN1 reference are used for the PCA analysis. These variants were filtered by “ --maf 0.05 --geno 0.05 --hwe 1e-6 midp --indep-pairwise 50 5 0.5 --snps-only --autosome” and PCA was done with PLINK (v1.9).^125^

Local ancestry inference was conducted using methods described by Sergey et. al.^79^ Briefly we called the CN1 variant on GRCh38 reference with dipcall, and variants are merged with 1KGP variant dataset downloaded (http://ftp.1000genomes.ebi.ac.uk/vol1/ftp/data_collections/1000_genomes_project/release/20181203_biallelic_SNV/). The ancestry was inferred to the super-population level in 1KGP, i.e., AFR, AMR, EAS, EUR, SAS using RFmix (v2.03).^126^ The ancestry of chrX PAR regions was also inferred using female individuals in 1KGP.

#### Benchmarking CN1-unique and CHM13-unique SNVs using HG002 and HG005

The bias introduced by using different reference genomes (i.e., CN1 and CHM13) was examined using the HG002 and HG005 datasets obtained from https://github.com/genome-in-a-bottle/giab_data_indexes. Raw sequencing data were subsampled to 5×, 10×, 15×, 20×, 25×, and 30× to illustrate the bias influenced by sequencing depth. Mapping and variant calling processes were performed as described above. To avoid the variants introduced by the SNVs among the three references (i.e., CN1, CHM13, and hg38), only heterozygous SNVs were considered. To obtain the unique SNVs called by using different references, all variants called on CN1 were projected onto CHM13 coordinates using the LiftoverVcf command in Picard package (v2.23.8, https://github.com/broadinstitute/picard). The variants on CHM13 were also compared using bedtools and bcftools to obtain the shared CN1-unique and CHM13-unique SNV dataset. SNVs within the centromere and heterochromatin regions were precluded from CN1-unique and CHM13-unique datasets.

The NIST dataset (v4.2.1) was utilized to benchmark the two variant sets called from different reference genomes (i.e., CN1 and CHM13). Briefly, HG002 (or HG005) variants were lifted over to hg38 using “LiftoverVcf” in Picard package. HG002 and HG005 truth set and confident regions were obtained from https://ftp-trace.ncbi.nlm.nih.gov/ReferenceSamples/giab/release/AshkenazimTrio/HG002_NA24385_son/NISTv4.2.1/GRCh38/ and https://ftp-trace.ncbi.nlm.nih.gov/ReferenceSamples/giab/release/ChineseTrio/HG005_NA24631_son/NISTv4.2.1/GRCh38/, and the variants were benchmarked using hap.py (v0.3.15, obtained from https://github.com/Illumina/hap.py). Since only heterozygous SNVs were considered, the benchmark VCF was filtered to contain only heterozygous SNVs. To further investigate the CN1-unique SNVs, SNVs in all reads in the BAM files were extracted using the 30× dataset as an example using a custom script, and the mapping coordinates in CHM13 were traced. The detailed process is shown in Supplementary Notes.

### Chinese cohort study

A total of 8869 Chinese samples from two cohort studies were included. These studies were reviewed and approved by the Ethics Committee of BGI (BGI-IRB 21163, BGI-IRB 20191). Read mapping and variant calling were conducted following the same method in 1KG sample analysis.^127^ VCF files for each sample were merged using bcftools. To improve the quality of called variants, the merged VCF files were filtered using HardFilter (QD < 2.0, MQ < 40.0, FS > 60.0, SOR > 3.0, MQRankSum < −12.5, ReadPosRankSum < −8.0). Variants with quality < 100, indel length > 20 bp, and SNPs within 10 bp distance from an indel were also filtered using bcftools. Besides, genotypes with depth < 10× or > 1.65-fold of the average depth of the individual base were considered missing, and genotypes with alternative allele depth proportion of a heterozygous variant (defined as DP_ALT_ /(DP_REF_ + DP_ALT_)) > 0.8 or < 0.25 were also set as missing. The bi-SNPs and their allele count (AC) were subsequently analyzed using bcftools view with ‘-m2 -M2 -v snps’. To test the difference in mapping quality and SNP variant quality, CN1_rare, CHM13_rare SNPs, and 2500 randomly selected Both_rare SNPs were compared using two-sided *t*-tests.

### Reference bias in detecting introgression from ancient hominin genomes

Raw genome sequences of Altai Neanderthal and Denisovan were downloaded from studies^68,70^ (https://www.eva.mpg.de/genetics/genome-projects/). Merged reads and paired-end reads having at least five bases with a quality of < 15 were excluded. Base qualities of any T base in the first two or last two bases for each read were reduced to two to avoid the effects of residual deamination. The filtered reads, with a minimum length of 30 bp, were then aligned against the reference genomes of CN1, CHM13, and GRCh38, first using BWA aln with parameters “-n 0.01 -l 16500 -o 2” and then using BWA samse.^128^ Reads with a mapping quality of < 30 were removed, and duplicate reads were marked using Picard and discarded. Alignments around small indels were realigned using GATK IndelRealigner,^129^ and raw variants were called based on the realigned BAM file. With the called variants as known sites, base-pair scores were recalibrated using GATK BaseRecalibrator and PrintReads. The recalibrated BAM files for all libraries were merged into one BAM file using samtools. Whole-genome 1 kb window mapping depth was calculated using samtools depth.

To profile the bias in mapping ancient genomic sequences using two gapless reference genomes CN1 and CHM13, each read was labeled as CN1-specific, CHM13-specific, or shared mapping read. Specific mapping reads were extracted from the BAM files, and the depth for each base was counted. Genomic regions with an average specific mapping depth greater than half of whole-genome coverage were merged using a custom script. Specific mapping regions with a length of < 100 bp were discarded. bi-SNPs with genotype quality > 30 for Altai Neanderthal and Denisovan genomes were called individually using the GATK pipeline. Variants in the two ancient human genomes, additional 60 modern human genomes from HGDP (including ten Bantu Kenya genomes in AFR, 20 Han genomes in EAS, 20 French in EUR, and ten Oceanian genomes), and five chimpanzee genomes^130^ were also jointly called for introgression analysis. To investigate the effect of reference bias on detecting introgression from ancient hominin genomes, ABBA-BABA tests were performed using CN1, CHM13, and GRCh38 as reference genomes, respectively.^71^ Under a given four-taxon topology “((P1, P2), P3, O)”, modified *D* statistics (*f*_d_) were calculated. Chimpanzee genomes were set as an outgroup O. P1 was set as Bantu Kenya from AFR. P2 was Han, French, or Bougainville population. P3 was Altai Neanderthal or Denisovan. The sliding windows were set with a size of 10 kb and a step size of 1 kb. Windows with < 30 good sites were discarded. Outlier windows were defined when *f*_d_ values exceeded an empirical cutoff of 0.35 and then merged into pIRs. A pIR with a length of at least 20 kb was retained. To compare the introgression detection bias between references, the windows in CHM13 or GRCh38 were first converted into the coordinates of CN1 using LiftOver, and the differences and sharing in pIRs were profiled using Bedtools intersect with different reference genomes and populations.

Local synteny around 13 Mb on chromosome 1 between CN1 and CHM13 and between CN1 and HPRC assemblies was evaluated using nucmer in MUMMER package. A total of 1493 modern human genomes from HGDP and SGDP were used to characterize the global distribution of the CN1-like insertion around the 13 Mb on chromosome 1, including 13 AFR populations (African Ancestry in Southwest USA, African Caribbean, Bantu Kenya, Bantu South Africa, Biaka, Esan, Gambian Mandinka, Luhya, Mandenka, Mbuti, Mende, San, and Yoruba), eight AMR populations (Colombian, Karitiana, Maya, Mexican Ancestry, Peruvian, Pima, Puerto Rican, and Surui), nine Central South Asia populations (Balochi, Brahui, Burusho, Hazara, Kalash, Makrani, Pathan, Sindhi, and Uygur), 20 EAS populations (Cambodian, Dai, Daur, Han, Hezhen, Japanese, Kinh Vietnamese, Lahu, Miao, Mongolian, Naxi, Northern Han, Oroqen, She, Southern Han Chinese, Tu, Tujia, Xibo, Yakut, and Yi), 13 EUR populations (Adygei, Basque, Bergamo Italian, British, CEPH, Finnish, French, Iberian, Orcadian, Russian, Sardinian, Toscani, and Tuscan), four Middle East populations (Bedouin, Druze, Mozabite, and Palestinian), three Oceania populations (Bougainville, Papuan Highlands, and Papuan Sepik), and five SAS populations (Bengali, Gujarati, Punjabi, Tamil, and Telugu). For each individual, reads with a mapping quality of < 30 when aligned against CN1 were excluded first, and average mapping depth from 12.952 Mb to 12.973 Mb on chromosome 1 was calculated and scaled individually based on whole-genome average depth. If the scaled depth in the insertion region was between 0.4 and 0.8, a heterozygous allele was defined. If the value was greater than 0.8, a homozygous CN1-like insertion allele was defined. The frequency was calculated for each of the 75 populations.

### Supplementary information

Supplementary Notes

Supplementary information, Figures

Supplementary information, Tables

## Data Availability

All raw sequencing data for CN1 analyzed in this study are available at GitHub: https://github.com/T2T-CN1/CN1. The reads are also deposited in the CNCB under the accession number HRA004405. The final CN1 curated assemblies are available in the CNCB with the accession number GWHCBHP00000000 under the BioProject ID PRJCA016397. The accession numbers for the maternal and paternal haplotypes are GWHCBHM00000000 and GWHCBHQ00000000 in CNCB, respectively. We also submitted the assemblies to CNGB with the accession numbers of CNA0069006–CNA0069008 for combined, maternal, and paternal genomes, respectively. The sample datasets are available on CNGB with the BioProject ID CNP0004252. Assemblies, annotation, and variant results are available at https://genome.zju.edu.cn/.

## Code availability

All customized codes and website sources can be accessed from https://github.com/T2T-CN1. Detailed use of codes is mentioned in Results, Materials and Methods and Supplementary Notes.
