# Supplementary material for: The complete and fully-phased diploid genome of a male Han Chinese
Source: Cell Res. 2023 Jul 14;33(10):745–61. doi: 10.1038/s41422-023-00849-5 (PMC10542383; doi:10.1038/s41422-023-00849-5)
Supplement: Supplementary file 1 — Supplementary Notes [file 41422_2023_849_MOESM1_ESM.pdf]

Supplementary Information

Supplementary Notes

Reads binning

The long reads were assigned to haplotypes to estimate the depth and assembly accuracy of diploid assemblies. The ONT ultra-long reads were assigned using canu (v2.2)[1] "`haplotype`" program with parental-specific kmer generated from MGI shotgun reads.

```
canu  -p asm -d CN1 \
      genomeSize=3g \
      useGrid=false \
      merylMemory=100 \
      merylThreads=24 \
      hapThreads=24 \
      hapMemory=100 \
      -haplotypePat $pat_ngs \
      -haplotypeMat $mat_ngs \
      -nanopore-raw $input \
      -stopAfter=haplotype
```

The ONT ultra-long reads were also assigned based on the trio-binning with parental-specific kmer produced from HiFi reads. The HiFi reads were used to generate 21 bp kmers using kmc (v3.2.1)[2] with the parameters "`-k21 -m500 -ci1`" and gain the preliminary specific kmers using filterx (https://github.com/ruanjue/filterx) with parameters "`-k s -1 'cnt=1'`". Referring to the criteria in canu "`haplotype`", the kmers with low frequency and the ONT reads less than 1000 bp in length were filtered. The remaining specific kmers were mapped to the ONT reads to determine the number of specific kmers in reads using "`map`" program (https://github.com/110allan/tod) with parameters "`map $ont.reads $specific.kmer`". To haplotype the ONT reads, the number of kmers in the reads was first normalized by dividing the total number of specific kmer. Additionally, the reads were haplotyped according to the appearance of parental specific kmer: 1) If NC_hap1 was greater than 0 and NC_hap2 was equal to 0, the read was classified into haplotype1; 2) If the rate of NC_hap1/NC_hap2 was greater than 1, the read was classified into haplotype1, where NC_hap1 means the normalized number of kmers from the haplotype1, and NC_hap2 means the normalized number of kmers from the haplotype2. Eventually, 4,600,086 ONT reads, including 2,283,106 paternal reads and 2,316,980 maternal reads, were haplotyped. The comparison of the two methods in haplotyping the ONT reads is shown below in "**rDNA analysis**".

A new pipeline was developed to improve the binning efficiency of HiFi reads, which were considerably shorter than Nanopore reads. The pipeline involves compression of parental HiFi reads by using

dehomopolymerate v.0.4.0 (https://github.com/tseemann/dehomopolymerate). The compressed HiFi reads were then cut into 201 bp kmers dataset using kmc with the parameters "`-k201 -m500 -ci1 $hifi_hpc $name $dir`" and compared with the sorted parental kmer sets using filterx (https://github.com/ruanjue/filterx) with parameters "`-k s -1 'cnt=1'`" to define the specific kmer-spectrum in the maternal or paternal genome. Additionally, the initially specific kmer-spectra with high frequency (>50) and low frequency (<2) were filtered. To obtain more specific kmers in the reads, the method permitting one base mismatch was established. The specific kmers in the reads were obtained by aligning reads to the specific kmers using bwa (v0.7.17-r1188) with parameters 'mem -a -M $MF2.fasta.gz $kmer.fa'. The above results were further processed based on the following three criteria. First, all kmers were filtered if they were mapped to the same position of reads with 1 bp mismatch. Second, all kmers with mismatch were filtered if they were mapped to the same position of reads except only one kmer without mismatch. Third, if only one kmer was mapped to the reads with 1 bp mismatch in the first or last location, the kmer was filtered. The reads were haplotyped based on the remaining mapping kmers in a read. 1) If C_hap1 was greater than or equal to 2 and C_hap2 was equal to 0, the read was classified into haplotype1; 2) If the ratio of C_hap1/C_hap2 was greater than or equal to 2, the read was classified into haplotype1. Eventually, 11,274,860 HiFi reads were haplotyped. Among them, 38.6% were paternal, and 38.5% were maternal.

Genome assembling

Contig construction

Initially, 70x PacBio HiFi data and 80x Nanopore ultra-long reads were used to build the backbone of the diploid genome using verkko (v1.0)[3] in trio mode with default parameters. Assemblies of 2.95 Gb and 2.86 Gb were generated for maternal and paternal genomes, respectively. Seventeen of these scaffolds constituted the complete chromosomes without any gaps. In addition 113 Mb sequences that were not phased by verkko were further phased using the canu "`haplotype`" program. Among them, 89 Mb were assigned to be maternal, and 18 Mb were assigned to be paternal. Moreover, a set of contigs with a length of 5.6 Mb were assigned to both maternal and paternal genomes because they were homozygous and formed the main path of the graph. We also constructed another assembly solely based on Pacbio HiFi reads using hifiasm (v0.16.1)[4] under trio mode.

Mitochondrial genome

The verkko assembly was mapped to the reference sequence NC_012920.1 to search for mitochondrial sequences. Two copies of the mitochondrial genome were assembled into a single scaffold. One of the mitochondrial genomes was confirmed by HiFi reads and kept as the mitochondrial genome.

Scaffolding

The scaffolding of verkko assemblies was enhanced using the hifiasm assembly. At first, verkko assemblies and hifiasm assemblies were separated into two haplotypes for subsequent analyses. For each haplotype, hifiasm and verkko assemblies were assigned to the respective chromosomes and placed along the coordinates by mapping to CHM13. Of note, CHM13 genome was used to merely help contig

placement, but not for reference-guided scaffolding (**Supplementary Note Fig. 1**). Within each chromosome, contigs were merged into a longer scaffold based on the overlap between hifiasm and verkko. After that, 6 (including chrM) and 9 gap-free chromosomes were generated in maternal and paternal genomes, respectively, and 18 and 14 chromosomes containing 30 and 39 gaps in the maternal and paternal genomes were obtained, respectively.

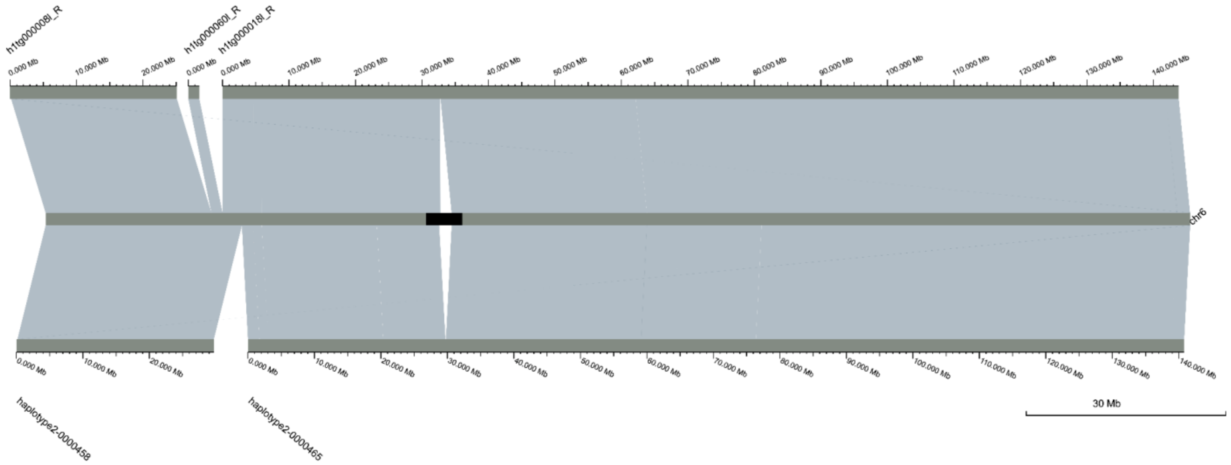

Supplementary Note Figure 1. Illustration of the scaffolding process for paternal chromosome 6 utilizing hifiasm assembly (above) to link verkko assembly (bottom). The hifiasm and verkko assemblies were aligned to CHM13 (middle) to organize and orientate the contigs. The black block in CHM13 indicated the centromere.

Local assembly of distal short arm of chr15

First, the ONT ultra-long reads were mapped to CHM13 using winnowmap2 (v2.03)[5], and the reads that were mapped to the distal short arm of chr15 were extracted using samtools (v1.11) with parameter "`-F 256`". Based on the binning information, the paternal reads were isolated and assembled using Flye (v2.9-b1774)[6]. Meanwhile, HiFi reads of chr15 telomeres were extracted (see below) and assembled using hifiasm. Both Flye assembly and hifiasm assembly were mapped to chr15 of CHM13 genome using unimap (v0.1-r41, https://github.com/lh3/unimap) with parameter "`-cxasm20`". A similar scaffolding strategy was applied to organize and orientate contigs. The contigs mapped to the same region of CHM13 were extracted and realigned using unimap. The redundant sequences were removed and connected together. Moreover, a gap was added if the adjacent contigs did not overlap and filled using the following gap-filling strategy.

Gap-filling

Before gap filling, diploid genomes were assembled using Flye from ONT ultra-long reads. The hifiasm assemblies, Flye assemblies, and binned ONT reads were used to manually fill a gap in sequence until the gap was filled successfully. We chose TGS-GapCloser (v1.2.1)[7] was chosen as the core gap closer; other complicated cases were examined manually in IGV (v2.14.1)[8] and visualization by LINKVIEW (https://github.com/YangJianshun/LINKVIEW). In addition, rDNA gaps were filled separately which is described in the section "rDNA sequence filling".

rDNA sequence filling

The assembly graph generated from verkko illustrated that most chromosomes were linear except for five acrocentric chromosomes (chr13, chr14, chr15, chr21, chr22) that were intertwined due to highly identical rDNA copies. The highly repetitive nature of rDNA and heterozygosity make it a challenge to solve. Thus, only the rDNA copy number was estimated to fill each chromosome with corresponding identical copies except chr21 and chr22 were directly assembled by verkko due to very few rDNA copies.

Telomere extension

Telomere analysis was conducted to define the completeness of telomeres using the vgp-assembly-master "`telomere_analysis.sh`" program with parameters "`0.5 5000`". Only one telomere was assembled on chr1 and chr15 in CN1.pat, chr3 in CN1.mat. Local genome assembly was conducted to gain the missing telomere. The location of the missing telomere was confirmed based on the alignment between CN1 draft genome and CHM13 and the positions of telomeres in CHM13. Firstly, the reads used to complete the local assembly were confirmed. 1) Based on the alignment of ONT reads and CHM13, ONT reads in 500 Kb sequence regions were obtained, including the missing telomere (chr3:200,605,948-201,105,948), and the paternal ONT reads (BGISEQ-BIN) were filtered. 2) h1tg000166l from the hifiasm assembly was aligned with the flanking sequences of missing telomeres. Moreover, based on the alignment of ONT reads and hifiasm assembly, the ONT reads were mapped to h1tg000166l, and the maternal ONT reads (BGISEQ-BIN) were filtered. Additionally, the vgp-assembly-master "`telomere_analysis.sh`" program with parameters "`0.5 10000`" was used to find ONT reads that contain telomere sequences. ONT reads unrelated to telomere were further filtered. 3) Based on the alignment of HiFi reads and CHM13, the HiFi reads in 1 Mb region were obtained, including the missing telomere (chr15:1-1,000,000), and the maternal HiFi reads were filtered. Then, these ONT reads in chr1 and chr3 were used to assemble the telomere via Flye, with parameters "`--nano-raw`", respectively. Next, the HiFi reads in chr15 were used to assemble the telomere via hifiasm. Eventually, the telomere on these local assemblies was annotated using vgp-assembly-master "`telomere_analysis.sh`" program with parameters "`0.5 5000`" (**Supplementary Note Fig. 2**).

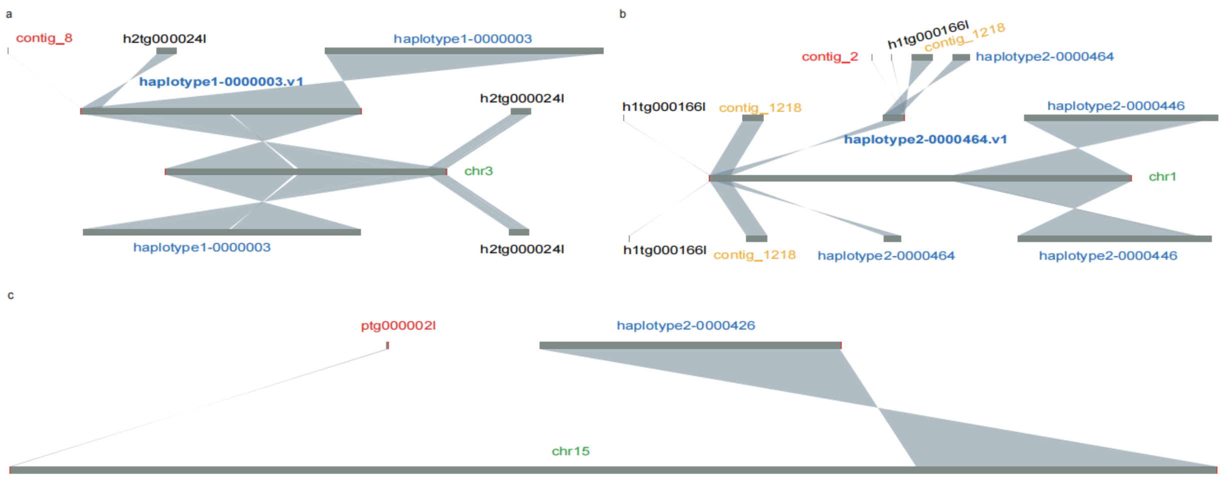

Supplementary Note Figure 2. Illustration of telomere extension in the maternal chr3 (a), paternal chr1 (b), and paternal chr15 (c). The small red blocks on the left indicated the telomere in CHM13, haplotype1-0000003.v1, and haplotype2-0000464.v1. The green, blue, and yellow, and black characters indicated CHM13, verkko, Flye, and hifiasm assemblies. The red character indicates the newly assembled sequences, which include telomere.

a, Hifiasm and verkko assemblies (bottom) were aligned to CHM13 chr3 to define the position of telomere, and contig_8, h2tg000024l and haplotype1-0000003 (above) were used to produce haplotype1-0000003.v1, which includes telomere.

b, Hifiasm, Flye, and verkko assemblies (bottom) were aligned to chr1 to define the position of the telomere, and contig_2, h1tg000166l, contig_1218 and haplotype2-0000464 (above) were used to produce haplotype2-0000464.v1, which includes telomere.

c, Verkko assemblies (above) were aligned to chr1 to define the position of the telomere and assemble the sequence ptg000002l, which includes telomeres.

Y chromosome

From the initial verkko assembly, 14 Y-linked contigs were identified based on the unimap alignment with CHM13. These Y-linked contigs were ordered and scaffolded by manually examining the ONT read alignments that were able to span two contigs with IGV and introduced 10 bp Ns as gap in-between every neighboring contigs. These ONT reads were then aligned again and used in the Y chromosome gap-filling process. Briefly, the ONT reads that cross two contigs were manually examined using IGV. Reads were then subtracted, aligned with MAFFT (v7.505)[9] with parameter set "`--globalpair --maxiterate 100`", called consensus with "`cons`" of the EMBOSS package (v6.6.0)[10]. The consensus sequences were then patched into the assembly (**Supplementary Note Fig. 3**). We noted that in some cases the initial assembly contained redundant sequences at the end of the contig (**Supplementary Note Fig. 3**). During the gap-filling procedure, these redundant sequences were removed.

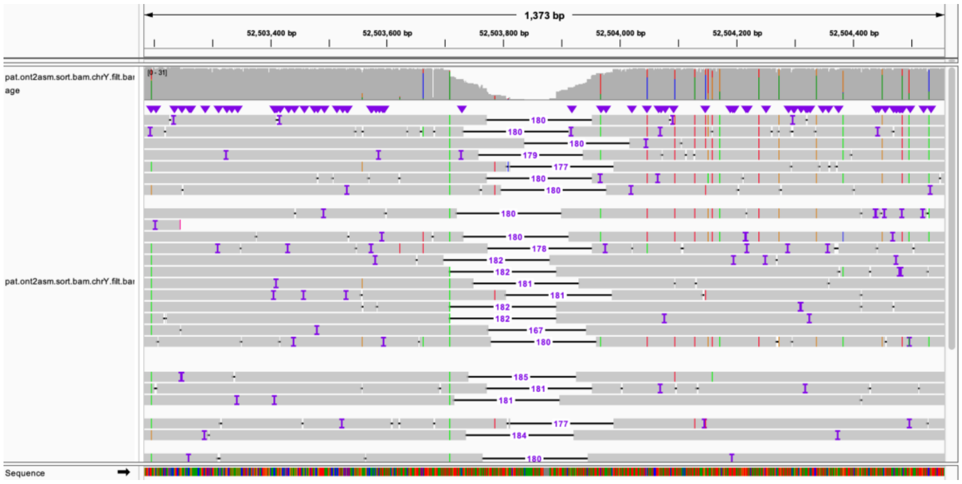

Supplementary Note Figure 3. ONT read alignment to the Y-linked contigs. Deletions were found in ONT reads, suggesting that the redundant sequences were at the end of the contig in the initial assembly.

Assembly validation using Hi-C

The haplotype-specific Hi-C reads were generated using the customized Hi-C trio-binning pipeline (https://github.com/BGI-Qingdao/HicTrioBinning), similar to the method described by Low et al.[11]. Specifically, all Hi-C reads were initially mapped to the haplotype-resolved TGS assemblies using BWA MEM (v0.7.12-1039)[12]. The Read1 and Read2 were separately aligned against two haplotype-resolved TGS assemblies, and only one alignment of the best hit was retained. Then, a haplotype score *MS* of each pair read was computed using the following formula:

$$MS_i = a\ log\ (A_{i,r1}) + b\ log\ (I_{i,r1}) + a\ log\ (A_{i,r2}) + b\ log\ (I_{i,r2})$$

where A represents the alignment length for the mapping result of the ith read pairs, and I is the identity ratio. The coefficients a and b are used to adjust the weight of A and I on the score and were empirically set to 1 and 3[7]. The unmapped reads were given a score of 0. Each read pair had two scores, one score for each haplotype. Read pairs with a higher MS score for maternal were considered maternal-specific, and vice versa. Read pairs with a tied MS score were considered homozygous and allocated to both haplotypes for assembly validation.

To validate our haplotype-resolved assemblies, the maternal- and paternal-specific Hi-C datasets were individually processed using HiC-Pro (v2.8.0)[13]. The two normalized contact maps were constructed for CN1 assembly validation using the 3D-DNA (v170123)[14] program with juicer (v1.5.0)[15] (**Supplementary Note Fig. 4**).

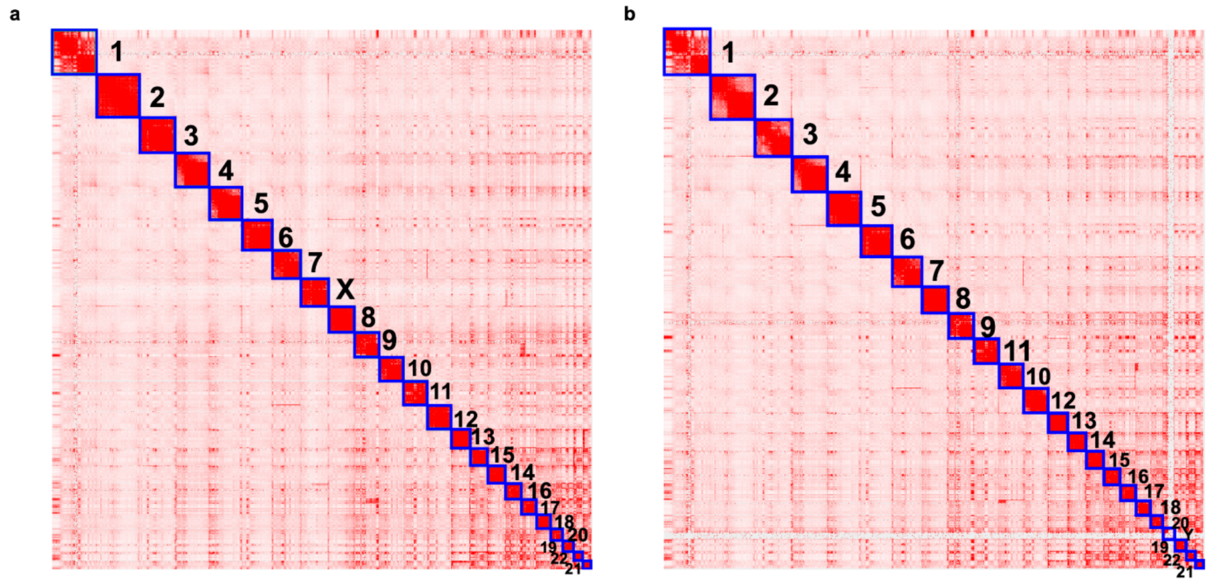

Supplementary Note Figure 4. CN1 Hi-C interaction matrix visualized and validated using CN1.mat (a) and CN1.pat (b) with juicebox.

Large structural variation validation using Bionano optical map

The large structural variations (> 1Mb) were also manually checked using Bionano optical map (a large inversion in Chr 8p as an example in **Supplementary Note Fig. 5**). At first, the Bionano molecules were binned by aligning proband molecules to parental haplotype assemblies to identify paternal and maternal allele molecules. Second, we realigner to maternal and paternal molecules to final maternal and paternal assemblies, respectively, using Bionano Access (version 1.5.2, https://us.bionanoaccess.com/) with the default alignment parameters. We used the function "View Molecules Alignment" in the Bionano Access to visualize the alignment to validate the assembly accuracy.

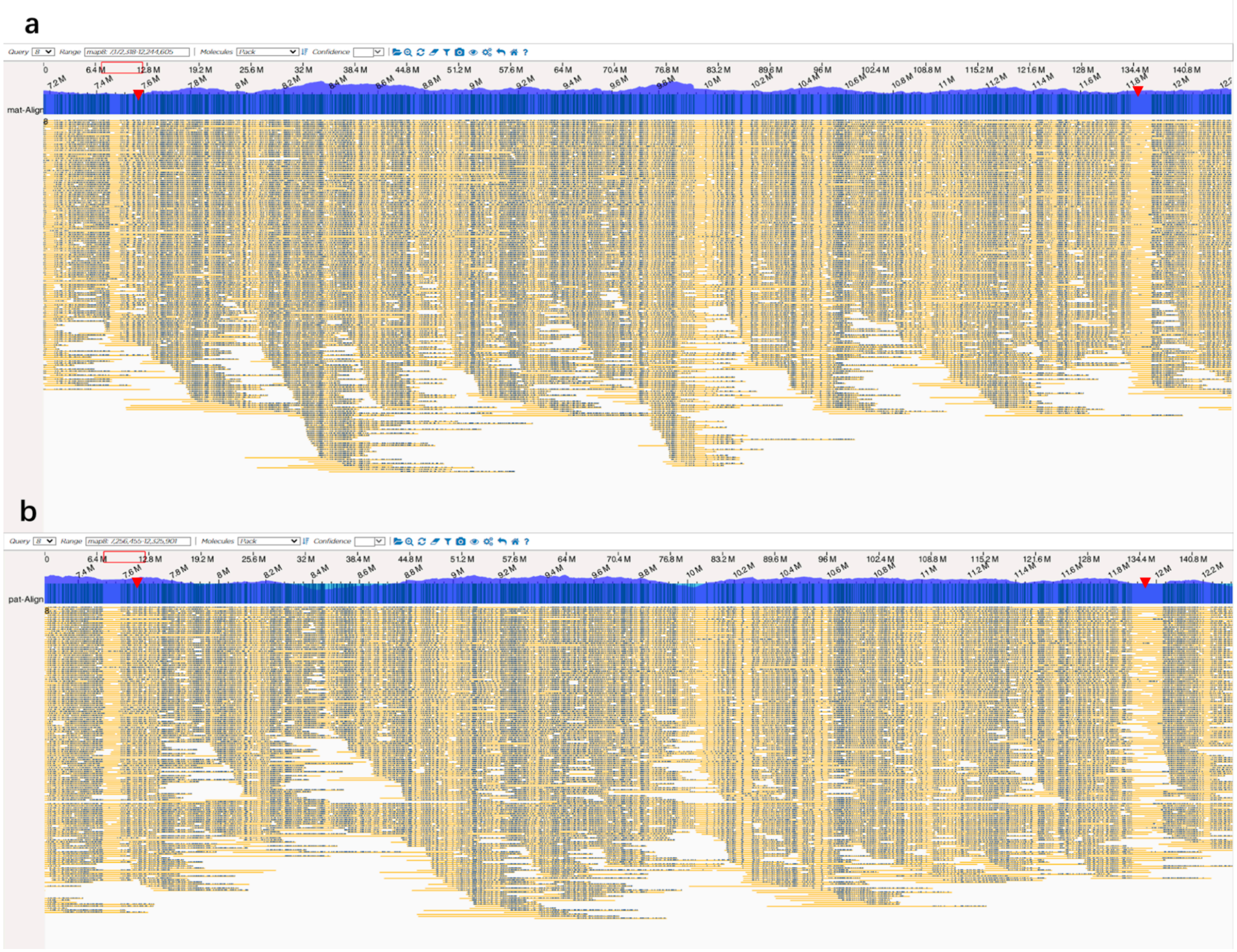

Supplementary Note Figure 5. Illustration of the homozygous large inversion and its 4 Kb flanking region in chromosome 8p using Bionano optical map. The coordinates are 7,572,319-11,844,606 and 7,656,453-11,925,899 in chromosome 8 of CN1.mat (**a**) and CN1.pat (**b**), respectively. The red arrows indicated the breakpoint locations. The continuous molecule coverage supports a real inversion between CN1 and CHM13.

rDNA variation analysis

Estimation of rDNA copy number using ddPCR

rDNA copy number of all trio samples was determined by using ddPCR. First, gDNA was linearized using restriction enzyme HaeIII (R0108S, NEB) according to the manufacturer's instructions. Then linearized gDNA was used as the template in ddPCR reactions to amplify rDNA and single-copy gene *TBP1* using specific primers and corresponding probes (**Supplementary Note Table 1**). Each reaction was prepared with 10 µL of 2x ddPCR Supermix for Probes, 7.2 µL of rDNA and *TBP1* primer mix (10 µM), 1 µL of rDNA and *TBP1* probe mix, 1 µL of gDNA (0.1-0.6 ng/µL), and 0.8 µL of ddH_2O. Droplets were generated after emulsification using a QX200 droplet generator (Bio-Rad, USA) following the manufacturer's instructions. PCR was conducted at conditions of pre-denaturation at 95°C for 10 min followed by 40 cycles of denaturation at 94°C for 30 s and extension at 60°C for 1 min, enzyme deactivation at 98°C for 10 min, and a final hold step at 4°C. After amplification, droplets were read using a QX200 droplet reader (Bio-Rad, USA) and quantified using the QuantaSoft software. rDNA copy number was calculated as the ratio of rDNA copy/µL to *TBP1* copy/µL.

Supplementary Note Table 1. Primers, restriction enzyme, gDNA concentration, and normalization gene used in repeated ddPCR array assays to validate copy count of rDNA in CN1.

Gene	Forward primer	Reverse primer	Probe	Probe dye (s)	Restriction enzyme	Tm
TBP1	5' - GATATGAGACTGTGGGTAAGT-3'	5' - GATCCTTTGAACACCCTAATG-3'	5' - TCTCAAAGCGTCAATT-3'	5' VIC and 3' MGB	HaeIII	56
rDNA	5'-AACGTGAGCTGGGTTTAG-3'	5'-CTCGTACTGAGCAGGATTAC-3'	5'-CACATCATCAGTAGGGT-3'	5' FAM and 3' MGB	HaeIII	56

Classification of rDNA models

The CHM13 genome contains eight models of rDNA morphs, including chr13, chr14, chr15a, chr15b, chr15c, chr21a, chr21b, chr22a[16]. Previous studies have shown more variations between rDNA units among different chromosomes than within the same chromosomes[16], which enables accurate assignment of rDNA reads to chromosomes. Thus, eight CHM13 models were used as the reference to classify rDNA reads in CN1 and identify new potential morphs using the detected large SVs (> 1 Kb indels). Given that each rDNA unit is approximately 44 Kb, we employed ONT reads (> 30 Kb) to identify the rDNA model. We used minimap2 to map ONT reads to eight reported rDNA models in CHM13 with parameters "minimap2 -ax map-ont -H -k 19 -B 5". For a note, "-H" and "-B 5" can improve the mapping accuracy for such very similar sequences. As a result, most ONT reads (6,620) can be unambiguously assigned to a certain rDNA model, with few reads returned more than one hit (26). These multiple hit reads can be further assigned according to identity and coverage.

As rDNA arrays are highly homogenized in the same chromosome, rDNA ONT reads were binned using the parental HiFi hamper to improve the phasing accuracy of rDNA copies (**Supplementary Note Fig. 6**, "**Read binning**"). After that, the haplotype rDNA copy number was inferred using the following formula:

$$250 * \frac{haplotype\text{-}specific\ and\ chromosome\text{-}specific\ ONT\ reads\ base}{total\ ONT\ base\ of\ rDNA},$$

where 250 was the total rDNA copies estimated by ddPCR.

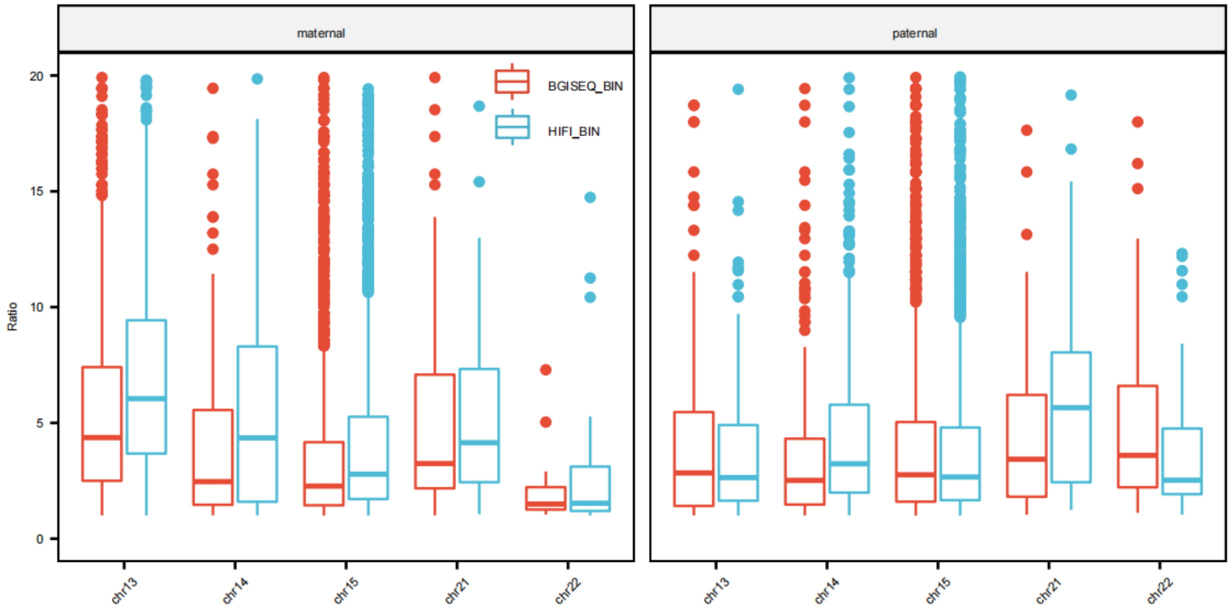

Supplementary Note Figure 6. Comparison of two binning methods for rDNA ONT reads. For each ONT read, the hapmer ratio was calculated according to canu binning result and HiFi binning result, respectively. For example, if a read was assigned to the maternal genome, the hapmer ratio was calculated as $Number_{maternal\text{-}specific\text{-}kmer}$ / $Number_{paternal\text{-}specific\text{-}kmer}$, and vice versa.

Polishing

From v0.4 to v0.6

Although it is preferable to polish the maternal (or paternal) genome using maternal (or paternal) reads, it is challenging to bin the short reads data. Only 77% HiFi and 88% ONT reads can be binned using the trio-binning strategy. Consequently, to minimize introducing switch errors during downstream polishing, the T2T polishing pipeline[17] (https://github.com/arangrhie/T2T-Polish/blob/master/doc/T2T_polishing_case_study.md) was adapted to polish CN1 genome, primarily using the variants called from binned ONT reads to determine the genotype and guide the selection of the heterozygous variants called from HiFi and short reads. Long reads (binned ONT and all HiFi data) were mapped using winnowmap2, and primary alignments were retained. MGISEQ short reads were mapped using BWA MEM, and duplications were marked using "`bamsormadup`" from biobambam2 (v2.0.183)[18]. Small variants were called using the "`hybrid`" model in DeepVariant (v1.4.0)[19] based on

the combined HiFi and MGISEQ alignments, and SNVs from binned ONT alignment were identified using PEPPERDeepVariant (r0.8)[20]. These variants were combined following the workflow shown in **Supplementary Note Figure 7**. Structural variants were called based on the binned ONT alignment using sniffles2 (v2.0.7)[21]. The region from both binned ONT alignment and unbinned HiFi alignment we extracted based on the ONT alignment and visualized using a modified version of bamsnap[22] (https://github.com/zy041225/bamsnap). The ONT and HiFi alignment was further manually examined to confirm the precise coordinate and size of each SV. These small variants and large SVs were then merged and used to polish the genome using merfin (v1.1)[23] and bcftools (v1.16)[24]. Two rounds of genome polishing (from v0.4 to v0.5, and from v0.5 to v0.6) were performed following the workflow to generate the v0.6 version of the genome (CN1_mat.v0.6.fasta and CN1_pat.v0.6.fasta).

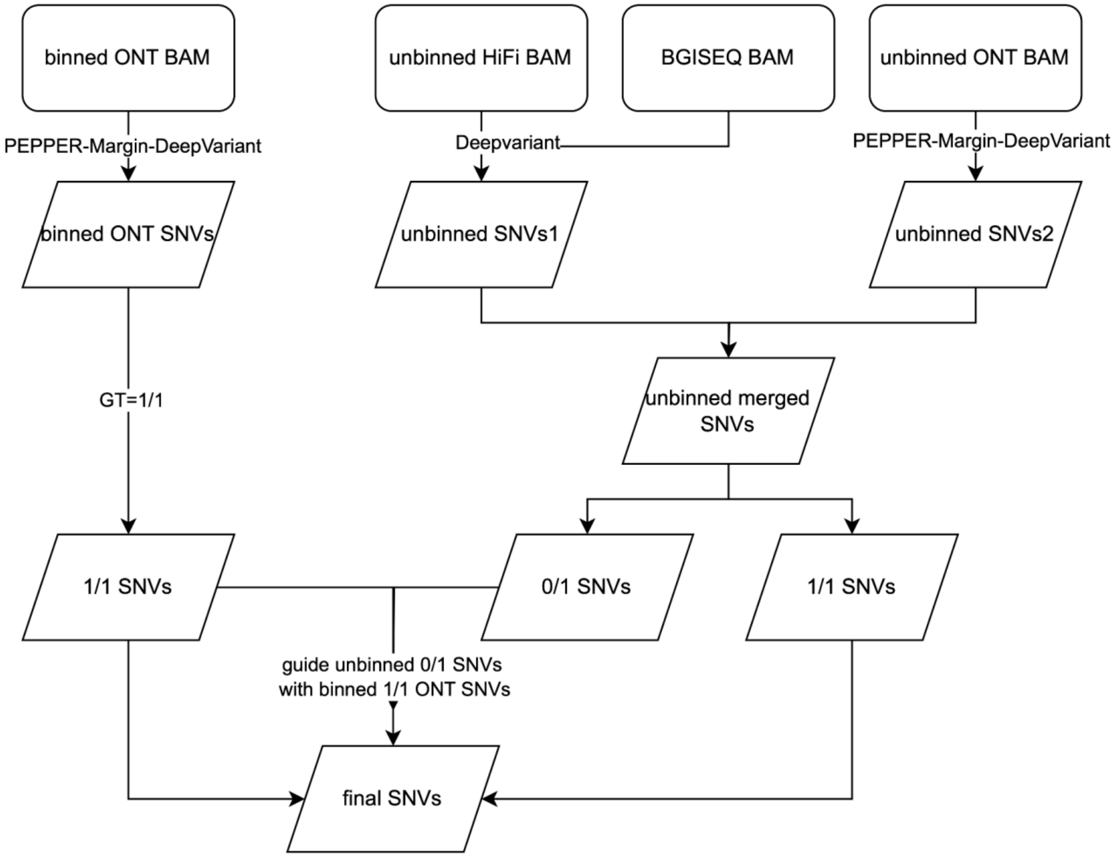

Supplementary Note Figure 7. The combined strategy for small variants to conduct polishing.

From v0.6 to v0.7.1

Based on the assembly-only kmer coordinates reported by merqury (v1.3)[25] (see below "**Assembly quality value (QV)**"), the binned HiFi and ONT read alignment at the region was further visualized using the modified bamsnap for manual examination. The variants called in the regions were further manually examined and used to polish the genome. Clipped regions were also identified and fixed during this round of polishing to generate CN v0.7.1 genome (see "**Resolving CLIP region**").

From v0.7.1 to v0.8

All HiFi reads were mapped to CN1 v0.7.1 maternal or paternal genome using winnowmap2. Illumina reads were mapped using BWA MEM, and variants were called using Deepvariant from the hybrid BAM alignment of HiFi and Illumina alignments. Variants marked as 'PASS' by deepvariant with VAF ≥ 0.5 were retained, and the called genotype was required to be '1/1'. These variants were further filtered using merfin and used to polish the maternal and paternal genome, and CN1 v0.8 genome was obtained.

From v0.8 to v0.8.1

Using merqury we found that the 5' end of chr15 in both maternal and paternal genomes were enriched with assembly-only kmer. Since the region was patched with sequences assembled with ONT reads using Flye, the sequence was replaced with a sequence assembled with HiFi reads using hifiasm. Hifiasm contigs were mapped to CN1 v0.8 genome using unimap. After manual examination of the alignment at chr15:0-10Mb, two and three contigs in maternal and paternal chr15 were replaced, respectively, and a final CN1 v0.8.1 genome was obtained.

Assembly quality value (QV)

The PacBio HiFi reads and Illumina PCR-free pair-end reads were utilized to construct a hybrid kmer dataset and evaluate the assemblies using merqury, following the example given at https://github.com/arangrhie/T2T-Polish/tree/master/merqury. The command to generate CN1 hybrid kmer dataset was shown below.

```
meryl memory=20g threads=20 greater-than 1 illumina.meryl output
illumina.gt1.meryl
meryl memory=20g threads=20 greater-than 1 HiFi.meryl output
HiFi.gt1.meryl
meryl memory=20g threads=20 divide-round 4 illumina.gt1.meryl output
illumina.gt1.div4.meryl
meryl memory=20g threads=20 divide-round 3 HiFi.gt1.meryl output
HiFi.gt1.div3.meryl
meryl memory=20g threads=20 union-max illumina.gt1.div4.meryl
HiFi.gt1.div3.meryl output hybrid.meryl
```

One haploid genome (CN1_combine.v0.6.fasta) was generated by combining the maternal and paternal assemblies based on assembly QV (**Supplementary Table 4**). In detail, the maternal or paternal sequences in v0.6 for each chromosome with higher QV were combined.

The publicly available data were downloaded to evaluate the QV of T2T-CHM13 and HG002 genome. T2T-CHM13 genome, except for chrY, was evaluated with HiFi and Illumina reads from SRP190633 and SRP051383. T2T-CHM13 chrY and HG002 genome were evaluated with HiFi and Illumina reads from https://s3-us-west-2.amazonaws.com/human-pangenomics/index.html?prefix=NHGRI_UCSC_panel/HG002/hpp_HG002_NA24385_son_v1/. The diploid HG002 genomes were combined according to the QV of each chromosome to compare HG002

and CN1 genomes. The assembly-only kmer coordinates on CHM13 (or HG002) were lifted over to CN1 using crossmap (v0.6.4)[26] and visualized along the CN1 genome using karyoploteR (v1.21.0)[27]. The liftover chain files were generated using nf-LO (v1.8.0)[28] following the description at https://s3-us-west-2.amazonaws.com/human-pangenomics/T2T/CHM13/assemblies/chain/v1_nflo/v1_nflo_description.html.

Coverage examination

The binned HiFi and binned ONT reads were mapped to the corresponding maternal (or paternal) genome using LRA (v1.3.2)[29], minimap2 (v2.24)[30], and winnowmap2. Primary alignments from each aligner were separately extracted and processed following the method described at https://github.com/arangrhie/T2T-Polish/tree/master/coverage. The regions were further merged based on the three HiFi (or ONT) results, and only the region was retained if two or more alignments obtained using LRA, minimap2, and winnowmap2 suggested a potential problem. The read alignment was further confirmed using the modified bamsnap and IGV to exclude false positives introduced by reads binning. The problematic HiFi and ONT regions were further combined only when one region showed abnormal coverage in both HiFi and ONT alignment. Most potential problematic regions were in the centromeres. Only the regions outside the centromeric regions were examined and confirmed.

Resolving CLIP region

To confirm and resolve the CLIP region, all related reads, including the primary and supplementary reads within the clipping region and their flanking 20 Kb regions, were extracted. In all these cases, the CLIP signals in the assembly were caused by redundant sequence, as the primary alignment and its supplementary alignments were usually mapped to regions flanking to the CLIP region. To resolve this issue, the same procedure for gap-filling in the Y chromosome was employed.

Resolving LOW region

To confirm the regions with low coverage in both HiFi and ONT alignments, the unique markers (k = 21) on the assembly and Illumina reads were generated following the T2T workflow at https://github.com/arangrhie/T2T-Polish/tree/master/marker_assisted. Related reads and markers at the LOW coverage regions and their flanking 40 kb region were extracted, and the markers were mapped to these reads with bowtie2 (v2.5.0)[31] with parameters "`--end-to-end --very-sensitive -a`". Marker alignments between assembly and raw reads were then visualized using LINKVIEW for syntenic confirmation, and the distance between these markers was examined. Our results showed that in all low-coverage regions, the marker distances in the reads were within 5% of the marker distances in the assembly. Thus, these regions with low coverages were not an assembly issue but more likely to be caused by sequencing bias. In one region of the v0.6 maternal assembly (CN1.mat chr3:199,878,656-199,879,680) and one region of the v0.6 paternal assembly (CN1.pat chr3:197,471,491-197,512,147), only reads mapped with markers from one flanking region were identified. In addition, seven regions in CN1.pat v0.6 were unable to be confirmed because the nearest markers were too distant (> 70 Kb) from the low-coverage region.

Genome annotation

Gene annotation

CHAIN files for lifting-over from hg38 (or CHM13v2.0) to CN1 genomes were generated following the instruction at http://genomewiki.ucsc.edu/index.php/Same_species_lift_over_construction. During the alignment, the hg38 scaffolds with the assigned chromosome were blasted to the corresponding chromosome in CN1. The sequences of the other unassigned scaffolds in hg38 were blasted to all sequences in CN1. The generated chain files were used in most analyses in this study. The liftover chain files were also generated from CHM13 to maternal/paternal genome using nf-LO following the description at https://s3-us-west-2.amazonaws.com/human-pangenomics/T2T/CHM13/assemblies/chain/v1_nflo/v1_nflo_description.html. These liftover chain files were available at https://genome.zju.edu.cn/Downloads .

The genes of CN1 T2T genome were annotated using the liftoff (v1.6.3)[32] and liftOver (v438) to project the GRCh38.p14 RefSeq v110 reference annotation onto the assembly. In addition, with uniprot_sprot (release-2022_05) human protein sequence as the protein alignments and Chinese RNA-seq transcriptome data as the transcript alignments, BRAKER (v2.1.6)[33] annotation, Augustus (v3.4.0)[34] annotation, and lifted annotation were integrated into the integrated gene data set using EVidenceModeler (v1.1.1)[35] (**Supplementary Fig. 4**). Combined with the annotation of tRNA by tRNAscan-SE (v2.0) and the annotation of other ncRNA using cmscan (INFERNAL 1.1.4) with Rfam (v14.9), a total of 59,589 genes were annotated in the haploid T2T Han genome (CN v0.8).

```
[liftoff v1.6.3] liftoff CN1.fa GRCh38.p14.fa -sc 0.95 -copies -g
GRCh38.p14.gff -polish -o CN1.liftoff.gff -chroms chroms.txt -f
type.txt -p 300

[liftOver kent source version 438 with gff format] liftOver -gff
GRCh38.p14.gff GRCh38.p14_to_CN1.chain.gz mapped.gff unmap.gff
[liftOver kent source version 438 with genePred format] liftOver -
genePred GRCh38.p14.gff.genePred GRCh38.p14_to_CN1.chain.gz
mapped.gff.genePred unmap.gff.genePred

[uniprot_sprot with exonerate version 2.2.0] exonerate --model
protein2genome --showvulgar no --showalignment no --showquerygff no --
showtargetgff yes --softmasktarget yes --percent 80 --targetchunkid 1
--targetchunktotal 100 -q uniprot_sprot.fasta.cut/uniprot_sprot.fasta
-t CN1.fa > CN1.fa.out

[Chinese RNA-seq transcriptome with StringTie v2.2.1 & TransDecoder
v5.6.0]
StringTie v2.2.1:
stringtie -p 48 ${each_library} -o ${each_library}.noguied.gtf
stringtie -p 48 --merge -o merged.gtf ./various_library.gtf.list
```

```
TransDecoder v5.6.0:
      TransDecoder/util/gtf_genome_to_cdna_fasta.pl merged.gtf CN1.fa >
merged.gtf.transcripts.fa
      TransDecoder.LongOrfs -t merged.gtf.transcripts.fa &&
TransDecoder.Predict -t merged.gtf.transcripts.fa
      TransDecoder/util/gtf_to_alignment_gff3.pl merged.gtf >
merged.gtf.gff3
      TransDecoder/util/cdna_alignment_orf_to_genome_orf.pl
merged.gtf.transcripts.fa.transdecoder.gff3 merged.gtf.gff3
merged.gtf.transcripts.fa > StringTie.TransDecoder.genome.gff3

[Chinese RNA-seq transcriptome with Trinity v2.2.1 & PASA v2.5.2]
Trinity v2.2.1:
      Trinity --seqType fq --max_memory 50G --left
${each_library}.fq1.clean.fq.gz --right
${each_library}.fq2.clean.fq.gz --CPU 6 --trimmomatic --full_cleanup
PASA v2.5.2:
      perl PASApipeline-v2.5.2/Launch_PASA_pipeline.pl -c
pasa.alignAssembly.Template.txt -C -R --ALIGNERS blat,gmap,minimap2 --
CPU 150 -g CN1.fa -t ./merged.Trinity.fasta
[BRAKER v2.1.6] perl BRAKER_2.1.6/scripts/braker.pl --species=CN1 --
cores=48 --genome=CN1.fa --bam=Chinese.RNA-seq.bamList --workingdir=$3
--softmasking

[Augustus v3.4.0] augustus --species=human --gff3=on CN1.fa >
augustus.gff

[EVidenceModeler v1.1.1]
perl EVidenceModeler-1.1.1/EvmUtils/partition_EVM_inputs.pl --genome
CN1.fa --gene_predictions gene_predictions.gff --protein_alignments
protein_alignments.gff --transcript_alignments
transcript_alignments.gff --segmentSize 2000000 --overlapSize 500000 -
-partition_listing partitions_list.out
perl EVidenceModeler-1.1.1/EvmUtils/write_EVM_commands.pl --genome
CN1.fa --weights weights.txt --gene_predictions gene_predictions.gff -
-protein_alignments protein_alignments.gff --transcript_alignments
transcript_alignments.gff --output_file_name evm.out --partitions
partitions_list.out > commands.list
perl EVidenceModeler-1.1.1/EvmUtils/recombine_EVM_partial_outputs.pl -
-partitions partitions_list.out --output_file_name evm.out
perl EVidenceModeler-1.1.1/EvmUtils/convert_EVM_outputs_to_GFF3.pl  --
partitions partitions_list.out --output evm.out  --genome CN1.fa
find . -regex ".*evm.out.gff3" -exec cat {} \; | bedtools sort -i - >
CN1.EVM.gff
```

[ncRNA]
tRNAscan-SE version 2.0:
```
tRNAscan-SE CN1.fa -o trnascan-se_out -f trnascan-se_struct -m
trnascan-se_stats -b trnascan-se.bed -a trnascan-se.fa -l trnascan-
se.log
grep -e pseudo -e Undet trnascan-se_out | awk '{print $1".tRNA"$2"-
"}' > trnascan-se_out.lowqual && grep -v -f trnascan-se_out.lowqual
trnascan-se.bed > trnascan-se.filtered.bed && gffread trnascan-
se.filtered.bed -F -o trnascan-se.filtered.gff3
```
other ncRNA with cmscan:
```
     cmscan --cut_ga --rfam --nohmmonly --tblout CN1.tblout --fmt 2 --
cpu 192 -Z ${Z} --clanin Rfam_14.9/Rfam.clanin Rfam_14.9/Rfam.cm
CN1.fa > CN1.cmscan
     perl infernal-tblout2gff.pl --cmscan --fmt2 CN1.tblout --all --
source infernal -E 1e-5 > CN1.infernal.ncRNA.gff3
     perl Infernal_parsed2curated.pl CN1.infernal.ncRNA.gff3 >
CN1.infernal.ncRNA.parsed.gff3
```

[FINAL Data Set]
Complement_annotations with AGAT v1.0.0:
```
perl AGAT/bin/agat_sp_complement_annotations.pl --ref ${liftoff} --add
${liftOver} --add ${tRNAscan-SE} --add ${ncRNA} -out FINAL.gff3
```
CDS_Phase with (GenomeTools) v1.6.0:
```
gt gff3 -sort -tidy -retainids FINAL.gff3 > FINAL.CDS_Phase.gff3
```

[annotation transfer from CN1 v0.6 to CN1 v0.8]
Liftoff v1.6.3:
```
liftoff -sc 0.95 -g CN1_v0.6.gff3 -polish -o CN1_v0.8.liftoff.gff3 -
chroms chroms.txt -exclude_partial -p 168 CN1_v0.8.fasta
CN1_v0.6.fasta
```

liftOver kent source version 438:
```
liftOver -gff CN1_v0.6.gff3 ./CN1.v0.6_to_v0.7.1.chain
CN1_v0.7.liftOver.gff3 CN1_v0.7.unmap.gff3 && liftOver -gff
CN1_v0.7.liftOver.gff3 ./CN1.v0.6_to_v0.7.1.chain
CN1_v0.8.liftOver.gff3 CN1_v0.8.unmap.gff3
```

Complement_annotations with AGAT v1.0.0:
```
perl AGAT/bin/agat_sp_merge_annotations.pl --gff CN1_v0.8.liftoff.gff3
--gff CN1_v0.8.liftOver.gff3 --out CN1_v0.8.gff3
```

Centromere analysis

Monomer classification and novel monomer identification

A relatively high divergence of CN1.mat S2C13/21H1L.1 was observed as compared to that of CHM13 (**Supplementary Figs. 10a** & **10b**) based on the raw monomer classification, suggesting the presence of some new types of monomers in the CN1.mat chr21. To explore this, all S2C13/21H1L.1 in CN1.mat chr21 were extracted and aligned using muscle (v3.8.31)[36] with default parameters. The consensus sequence was obtained using "`cons`" in the EMBOSS package (v6.6.0) with default parameters. Then, all these monomers with the consensus sequence were aligned using "`needle`" in the EMBOSS package, and the alignments were converted to a 0-1 matrix, where 0 indicates a match, and 1 indicates a mismatch compared to the consensus sequence. A HartiganPlot generated using "`PlotHartigan`" from 'useful' (v1.2.6) R package showed two distinct clusters (**Supplementary Fig. 10c).** The best two clusters were identified using the "`FitKMeans`" from the same R package and validated using the monomer phylogenetic tree, which was built and visualized using MEGA7[37], with "`Minimum-Evolution tree, Model/Method = p-distance, Substitutions to Include=Transitions+Transversions, Rates among Sites=Uniform Rates, Gap/Missing data Treatment = Partial deletion, Site Coverage Cutoff=95, ME Search level = 1`". No considerable mixtures of monomers from different clusters were found (**Supplementary Fig. 10d**). Finally, the consensus sequences of the two clusters were generated. The monomer phylogenetic tree was constructed from these two consensuses, and other monomer consensus sequences were obtained from the three haploid chr21. These findings revealed that one of the two clusters was an analog of the original S2C13/21H1L.1, and the other formed a new monomer group, which was named S2C13/21H1L.1#. Then, these two consensus sequences (i.e., mat.S2C13/21H1L.1 and mat.S2C13/21H1L.1#) were added to the hmm-profile and reannotated the active HOR array of CN1.mat chr21. The intra-monomer divergence was then recalculated and visualized (**Supplementary Fig. 10e**). The monomers in all CN1 HiFi reads were used as a new hmm-profile to annotate, and the number of the monomer S2C13/21H1L.1 and S2C13/21H1L.1# in the binned HiFi reads were counted. The results revealed 64,708 S2C13/21H1L.1# monomers in the maternal reads and 337 in the paternal reads. In contrast, there were 11,525 and 12,838 maternal and paternal reads in S2C13/21H1L.1, respectively. These findings indicated that the new monomer S2C13/21H1L.1# might only exist in the CN1 maternal genome.

Chromosome 17 also contained a relatively high divergent monomer, S3C17H1L.15, in both CN1.mat and CN1.pat. The CN1.mat S3C17H1L.15 was further classified into subgroups using a similar method applied to S2C13/21H1L.1, and the corresponding graphs were available in **Supplementary Fig. 9**. The profile was then updated and used to annotate both maternal and paternal active HOR arrays in chr17 and recalculate the intra-array divergence (**Supplementary Fig. 9**). Moreover, the new profile was used to annotate the HiFi reads, revealing 85,880 and 92,091 paternal and maternal reads containing S3C17H1L.15#, respectively.

Heterozygosity analysis of centromere regions

There is no agreement yet on which aligner can fully resolve the alignment for centromeric satellites. Here we try minimap2 and TandemAligner (v0.1)[38] to perform the alignment and call the variants in centromeric regions. For details, we use the "-x asm5" setting for minimap2 alignment and followed by "paftools.js call" with default parameters to call all variants, and we use the TandemAligner with default parameter to compare the corresponding centromere regions of maternal and paternal CN1 genome. The results of the two software show that the heterozygosity rate at the SNV level is a little higher than that in the non-centromeric regions: averagely 1.65-fold (minimap) and 1.35-fold (TandemAligner) compared to the non-centromeric regions. However, when we focus on SVs (> 50 bp), the heterozygosity rate in the centromere region is much higher compared to the non-centromeric region (~5-fold for minimap2 and ~65-fold for TandemAligner). Specifically, the heterozygosity rate is about 1 SV per 347 Kb in the non-centromeric regions; the rate in centromere regions is about 1 SV per 64 Kb estimated from minimap and 1 SV per 5.3 Kb estimated from TandemAligner. To make a conservative estimation, we used the results from minimap2 for this section in the main text.

Resequencing data analysis

Gene clipping rate in different references

Genes are highly conserved and have functional significance compared to intergenic regions. Thus, the number of clipping reads was used as a proxy to investigate the mapping performance of gene regions. To filter out reference-specific annotated genes with multiple copies within a single reference, genes from GRCh38, CHM13, and CN1 (v0.6) were compared. The number of clipping reads within the gene body of the remaining 17,874 shared and single-copy genes were counted and the average number of clipping reads for each gene in each population was calculated. The proportion of genes with more clipping reads on CN1 or CHM13 varied among different super-populations, with EAS possessing the highest number of genes with more clipped reads in CHM13, while Middle East and EUR possessing the highest number of genes with more clipping reads in CN1 (**Supplementary Note Fig. 8**). Most genes exhibited similar clipping read numbers on CN1 and CHM13, except for 33 genes in EAS (**Supplementary Note Fig. 9**) with excess (e^2 times more) clipping reads on CN1 than CHM13 and 26 genes with excess clipping read numbers on CHM13 than CN1. These genes with excess clipping reads on either CN1 or CHM13 reference in EAS, which totaled 27 and 20, respectively, entirely overlapped with those in EUR with excess clipping reads on either CN1 or CHM13 (**Supplementary Note Fig. 9**). The gene set with excess clipping reads, which overlapped in different populations, cannot be explained by population-specific variation and is mostly characterized by insertions. For example, the *MEGF9* gene in CHM13 contained a LINE1 insertion of 1.4 kb (chr9:132,861,863-132,863,277). The possibility of misassembly of CHM13 was ruled out by checking the HiFi long reads alignment (**Supplementary Note Figs. 10 & 11**). These insertions were reference-specific and affected the mapping process for resequencing samples. Moreover, fewer such causing-clipping insertions were identified in CN1 than in CHM13, suggesting better mapping performance for gene regions in CN1 than in CHM13.

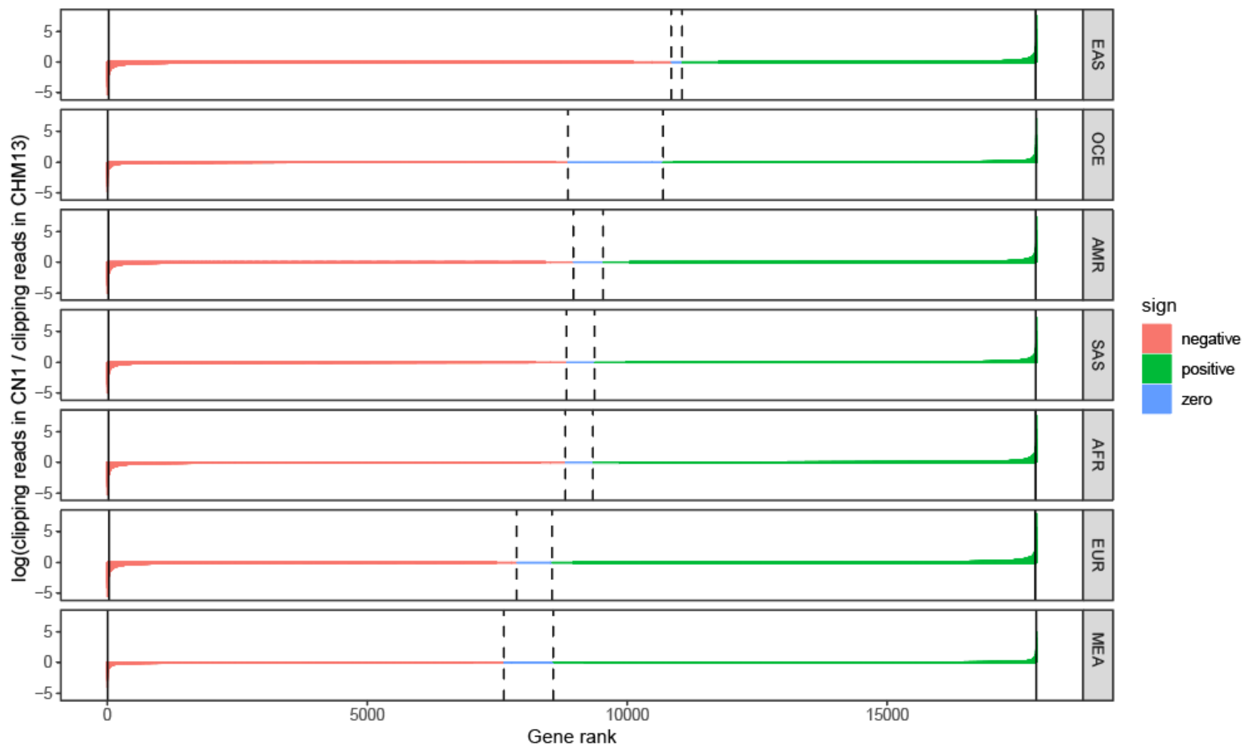

Supplementary Note Figure 8. The number of clipping reads of each gene in different super-populations. For each gene, the number of clipping reads is counted in both CN1 and CHM13. The 17,874 genes were numerically sorted based on the difference in the number of clipping reads as $log\ (CN1/CHM13)$. The y-axis is represented with red, green, and blue bars indicating the negative, positive, and zero coordinates, respectively, while the dash-line indicates transitions to another category. Each bar represents a gene, and the difference is shown along with the corresponding super-population.

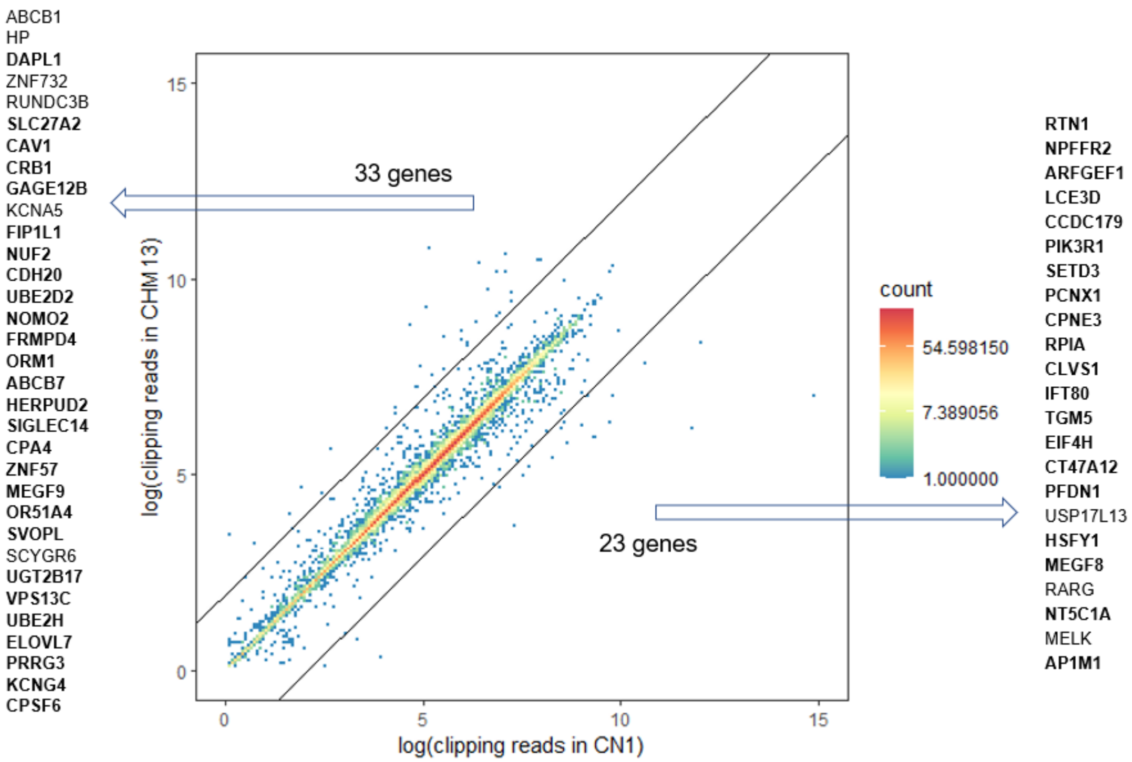

Supplementary Note Figure 9. Genes with an excess number of clipping reads in either CN1 or CHM13 for the EUR population. The dot outside the regions within x-2 < y < x+2 indicates genes with an excess number of clipping reads. Gene symbol IDs are listed by the side, and those overlapping with genes that also have excess clipping reads in EAS (27/33 and 20/23) are highlighted in bold font. A total of 17,874 genes are involved.

MEGF9

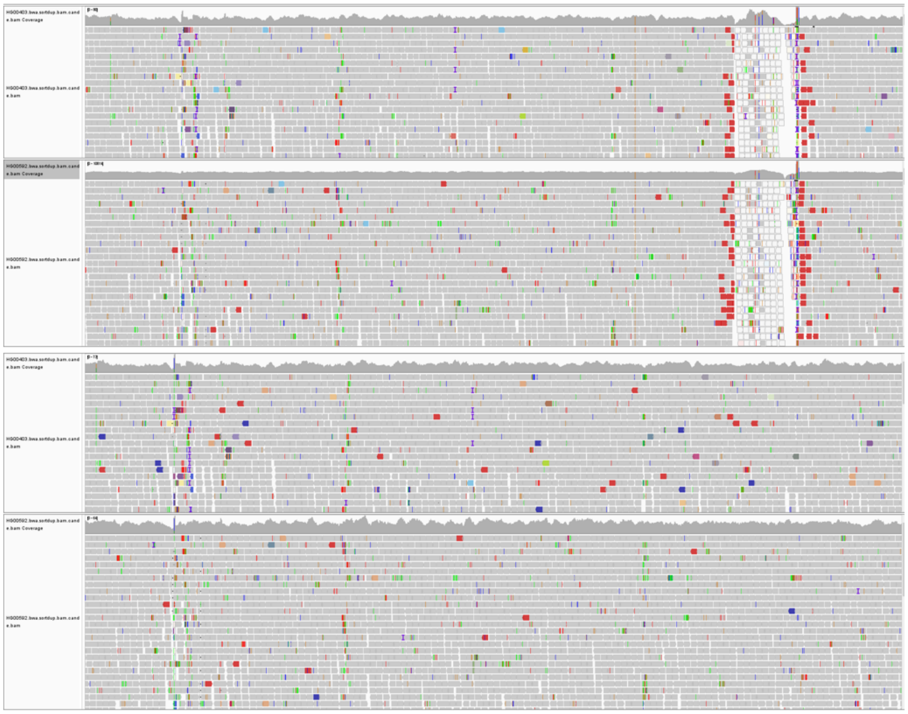

Supplementary Note Figure 10. Clipping reads caused by an insertion within *MEGF9* in CHM13. Two randomly chosen samples were aligned to the WGS data near the insertion and visualized using IGV. The top two tracks show the alignment of these two samples on CHM13 (chr9:132,856,138-132,867,484), while the bottom tracks show the alignment on CN1 (v0.6) (chr9:120,105,015-120,115,401).

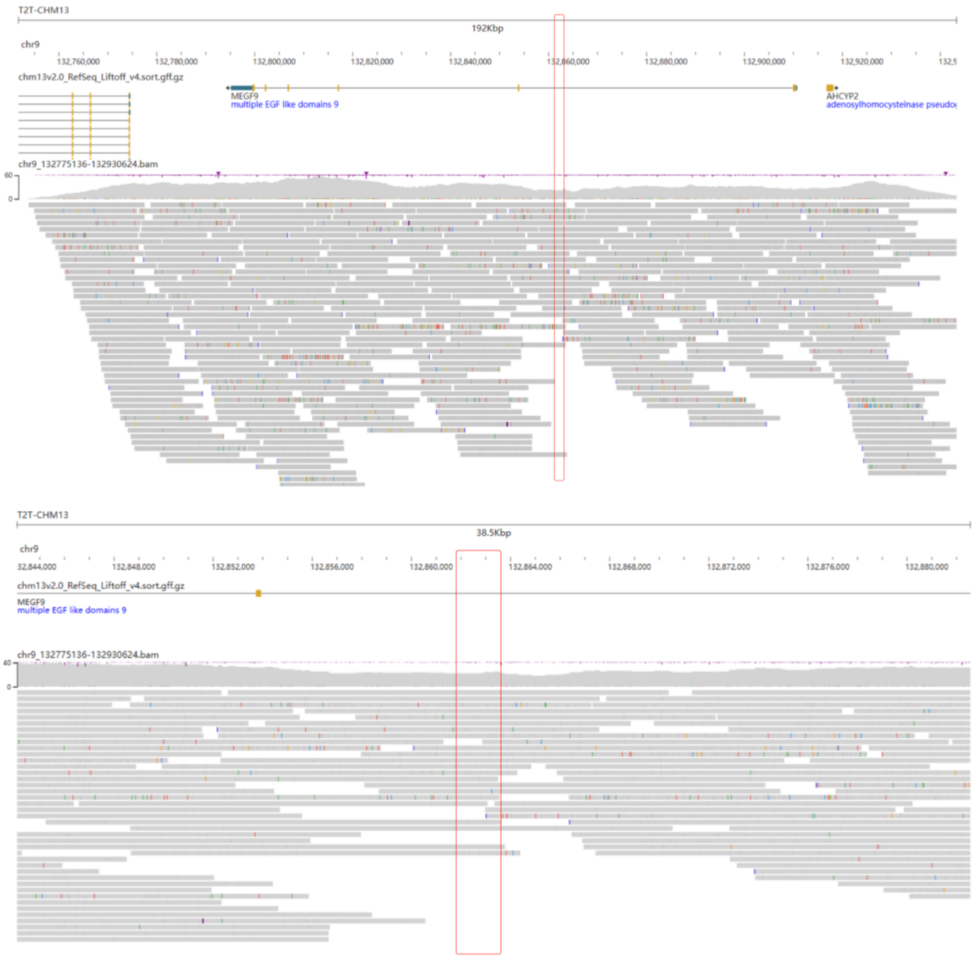

Supplementary Note Figure 11. Verification of the *MEGF9* insertion using CHM13 HiFi long reads. Alignment of CHM13 HiFi long reads near the insertion of *MEGF9* in the CHM13 reference is extracted. Red rectangles indicate the insertion region and the bottom panel shows a zoom-in view.

Reads uniquely mapped to either CN1 or CHM13

We recalculated the uniquely mapping reads number for either CN1 and CHM13 by excluding reads that are mapped to the centromere. The result shows that the ratio difference is only reduced a bit, thus the centromeric region is not the major course of this pattern. It is reasonable for some populations like AMR and OCE to also have a higher mapping rate in CN1 genome because of their closer genetic relationship with EAS. We should acknowledge that the mapping rate difference on other populations like SAS, MEA, EUR is very small.

And we further analyzed the distribution of uniquely mapping reads for HG002 and HG005, representing European and East Asian ancestry respectively. The genomic sequences are classified into copy-gain (CPG), copy-loss (CPL), other SVs (Other_SV), centromere (Cent), other remaining sequences (Other), according to the comparison between CN1 and CHM13. This result shows that most uniquely mapping reads reside in CPG between the two references. We further calculated the coverage depth for each base

in CPG regions on CN1 and CHM13 using 5× sequencing data for HG002 and HG005. In CPG regions, for HG002 (European) coverage depth on CN1 and CHM13 are similar, while for HG005 (East Asian), coverage depth in CHM13 is a bit lower than expected, indicating an overestimate of copy number on CHM13 (**Supplementary Note Table 2, Supplementary Note Figs. 12 & 13**).

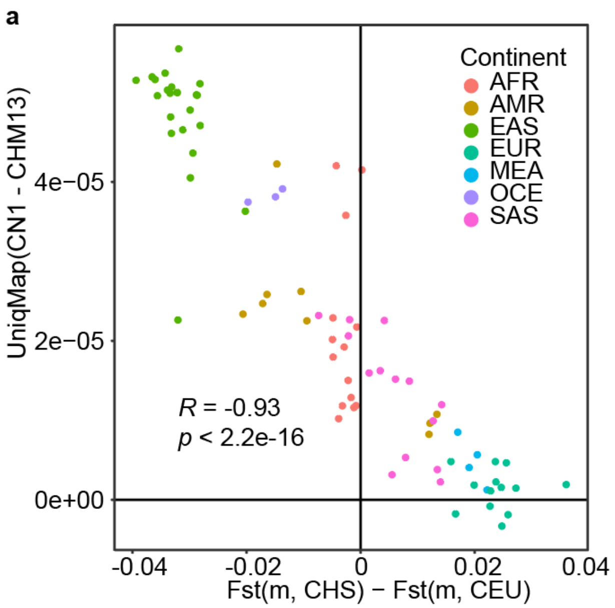

Supplementary Note Figure 12. The difference between the rate of uniquely mapped reads on CN1 and on CHM13 is correlated with the genetic relationship difference (a). The result is similar when reads in centromere are excluded (b).

Supplementary Note Table 2. The count of reads that are uniquely mapped to either CN1 or CHM13 in different region classes. 5× sequencing data for HG002 and HG005 were used.

Class	HG002		HG005	
	CN1	CHM13	CN1	CHM13
**CPG**	13,658	11,796	16,113	9,030
**CPL**	8	5	48	26
**Other_SV**	438	111	550	137
**Cent**	773	496	1,503	975
**Other**	354	1,331	4,114	4,497
**Total**	15,223	13,734	22,280	14,639

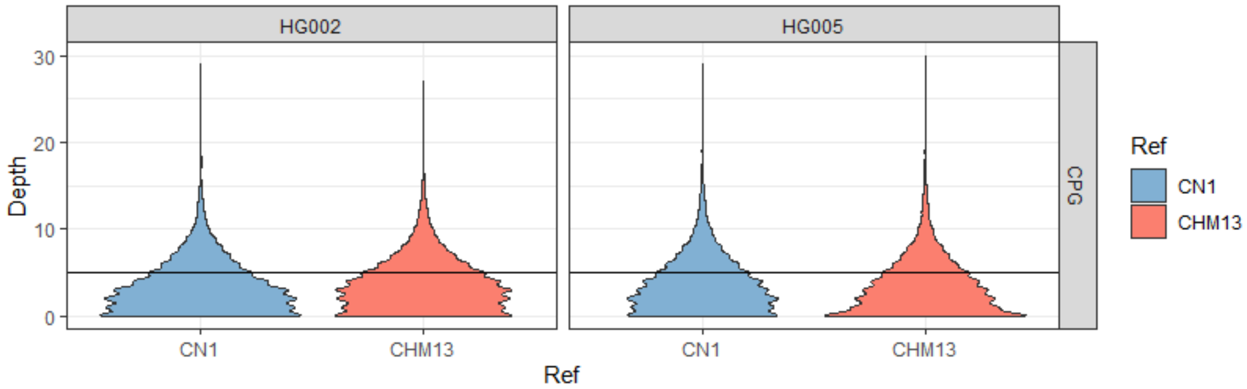

Supplementary Note Figure 13. The coverage depth for CPG regions of CN1 and CHM13. 5× sequencing data HG002 and HG005 are used (horizontal line).

Unique SNV Classification

To further investigate the CN1 unique SNVs, all reads contributing to each SNV were extracted from the BAM files of the 30x dataset using a custom script, and their mapping coordinates in CHM13 were traced. The covered reads of each heterozygous SNV were assigned to the reference type read (RTR) group or alternative type read (ATR) group according to their genotype. Both RTR and ATR groups for each SNV were classified by their mapping status. 1) Reads that mapped to more than one locus with > 2x depth in CHM13 and their top two loci had a depth ratio of (most depth / second most) <2 were defined as multiple loci-mapping reads and the top two covered loci were reported; 2) Reads that mapped to one locus with > 2x depth in CHM13 and their top locus had a depth ratio >2 were defined as single locus-mapping reads, and their most covered locus was reported. Unmapped reads were assigned to *null* and counted. According to the mapping status for both RTR and ATR groups, CN1 unique variants were classified into seven categories (**Supplementary Note Fig. 14** & **Supplementary Table 37**). 1) Unique SNVs with < 4 RTR were classified as *RefLow*; 2) Unique SNVs that were not mapped to either ATR or RTR were classified as *Unmap*; 3) Unique SNVs with either RTR or ATR mapped to multiple loci were classified as *TargetDup1*. 4) Unique SNVs that had ATR's locus congruent with RTR's locus and were also called as heterozygous SNVs in CHM13 were classified as *Alias* and removed from the final unique SNV dataset and **Supplementary Table 37**. 5) Unique SNVs that had ATR's locus congruent with RTR's locus and were not called heterozygous in CHM13 were classified as *Uncall*. 6) SNVs with ATR's locus within 50 bp from RTR's locus were classified as *Indel*. 7) SNVs with ATR's locus > 50 bp from RTR's locus were classified as *TargetDup2*. *TargetDup1* and *TargetDup2* were merged and classified as *TargetDup*. A similar analysis was performed to classify the CHM13 unique variants based on the related read alignment in CN1. Since most of the unique SNVs were *TargetDup*, their genomic coordinates were further examined using the SVs between CN1 and CHM13 (see "**Genomic comparison between CN1 and CHM13**" in **Materials and Methods**) and checked if they resided in the CNV regions, including indels, copy gains, and copy loss.

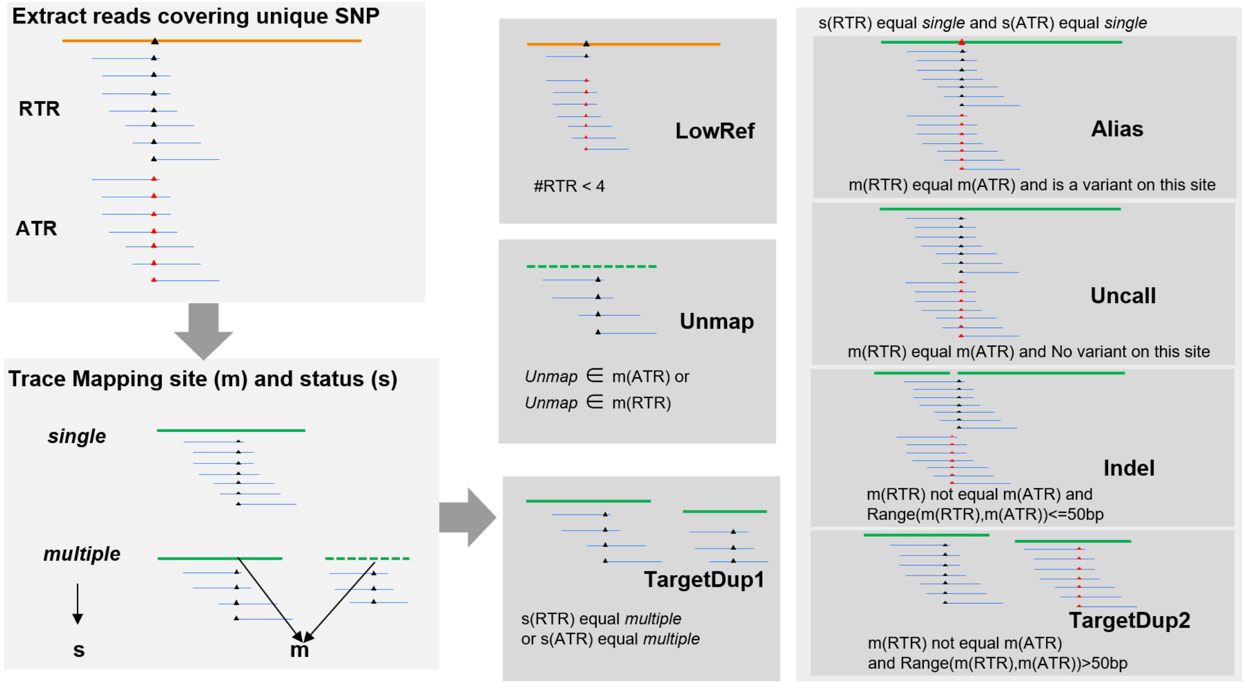

Supplementary Note Figure 14. Schematic process of unique SNVs assignment based on reads alignment. The orange line indicates the reference A, the green line indicates the reference B, and the blue lines represent sequencing reads. The black and red triangles denote the reference and alternative alleles on reference A, respectively.

Reference

1 Koren, S. *et al.* De novo assembly of haplotype-resolved genomes with trio binning. *Nat Biotechnol* (2018). https://doi.org:10.1038/nbt.4277
2 Kokot, M., Dlugosz, M. & Deorowicz, S. KMC 3: counting and manipulating k-mer statistics. *Bioinformatics* **33**, 2759-2761 (2017). https://doi.org:10.1093/bioinformatics/btx304
3 Rautiainen, M. *et al.* Telomere-to-telomere assembly of diploid chromosomes with Verkko. *Nat Biotechnol* (2023). https://doi.org:10.1038/s41587-023-01662-6
4 Cheng, H., Concepcion, G. T., Feng, X., Zhang, H. & Li, H. Haplotype-resolved de novo assembly using phased assembly graphs with hifiasm. *Nat Methods* **18**, 170-175 (2021). https://doi.org:10.1038/s41592-020-01056-5
5 Jain, C., Rhie, A., Hansen, N. F., Koren, S. & Phillippy, A. M. Long-read mapping to repetitive reference sequences using Winnowmap2. *Nat Methods* **19**, 705-710 (2022). https://doi.org:10.1038/s41592-022-01457-8
6 Kolmogorov, M., Yuan, J., Lin, Y. & Pevzner, P. A. Assembly of long, error-prone reads using repeat graphs. *Nat Biotechnol* **37**, 540-546 (2019). https://doi.org:10.1038/s41587-019-0072-8
7 Xu, M. *et al.* TGS-GapCloser: A fast and accurate gap closer for large genomes with low coverage of error-prone long reads. *Gigascience* **9** (2020). https://doi.org:10.1093/gigascience/giaa094
8 Thorvaldsdottir, H., Robinson, J. T. & Mesirov, J. P. Integrative Genomics Viewer (IGV): high-performance genomics data visualization and exploration. *Brief Bioinform* **14**, 178-192 (2013). https://doi.org:10.1093/bib/bbs017
9 Katoh, K. & Standley, D. M. MAFFT multiple sequence alignment software version 7: improvements in performance and usability. *Mol Biol Evol* **30**, 772-780 (2013). https://doi.org:10.1093/molbev/mst010
10 Rice, P., Longden, I. & Bleasby, A. EMBOSS: the European Molecular Biology Open Software Suite. *Trends Genet* **16**, 276-277 (2000). https://doi.org:10.1016/s0168-9525(00)02024-2
11 Low, W. Y. *et al.* Haplotype-resolved genomes provide insights into structural variation and gene content in Angus and Brahman cattle. *Nat Commun* **11**, 2071 (2020). https://doi.org:10.1038/s41467-020-15848-y
12 Li, H. Aligning sequence reads, clone sequences and assembly contigs with BWA-MEM. *arXiv preprint arXiv:1303.3997* (2013).
13 Servant, N. *et al.* HiC-Pro: an optimized and flexible pipeline for Hi-C data processing. *Genome Biol* **16**, 259 (2015). https://doi.org:10.1186/s13059-015-0831-x
14 Dudchenko, O. *et al.* De novo assembly of the Aedes aegypti genome using Hi-C yields chromosome-length scaffolds. *Science* **356**, 92-95 (2017). https://doi.org:10.1126/science.aal3327
15 Durand, N. C. *et al.* Juicer Provides a One-Click System for Analyzing Loop-Resolution Hi-C Experiments. *Cell Syst* **3**, 95-98 (2016). https://doi.org:10.1016/j.cels.2016.07.002
16 Nurk, S. *et al.* The complete sequence of a human genome. *Science* **376**, 44-53 (2022). https://doi.org:10.1126/science.abj6987
17 Mc Cartney, A. M. *et al.* Chasing perfection: validation and polishing strategies for telomere-to-telomere genome assemblies. *Nat Methods* **19**, 687-695 (2022). https://doi.org:10.1038/s41592-022-01440-3
18 Tischler, G. & Leonard, S. biobambam: tools for read pair collation based algorithms on BAM files. *Source Code for Biology and Medicine* **9**, 1-18 (2014).

19 Poplin, R. *et al.* A universal SNP and small-indel variant caller using deep neural networks. *Nat Biotechnol* **36**, 983-987 (2018). https://doi.org:10.1038/nbt.4235
20 Shafin, K. *et al.* Haplotype-aware variant calling with PEPPER-Margin-DeepVariant enables high accuracy in nanopore long-reads. *Nat Methods* **18**, 1322-1332 (2021). https://doi.org:10.1038/s41592-021-01299-w
21 Moritz, S. *et al.* Comprehensive Structural Variant Detection: From Mosaic to Population-Level. *bioRxiv*, 2022.2004.2004.487055 (2022). https://doi.org:10.1101/2022.04.04.487055
22 Kwon, M., Lee, S., Berselli, M., Chu, C. & Park, P. J. BamSnap: a lightweight viewer for sequencing reads in BAM files. *Bioinformatics* **37**, 263-264 (2021). https://doi.org:10.1093/bioinformatics/btaa1101
23 Formenti, G. *et al.* Merfin: improved variant filtering, assembly evaluation and polishing via k-mer validation. *Nat Methods* **19**, 696-704 (2022). https://doi.org:10.1038/s41592-022-01445-y
24 Danecek, P. *et al.* Twelve years of SAMtools and BCFtools. *Gigascience* **10** (2021). https://doi.org:10.1093/gigascience/giab008
25 Rhie, A., Walenz, B. P., Koren, S. & Phillippy, A. M. Merqury: reference-free quality, completeness, and phasing assessment for genome assemblies. *Genome Biol* **21**, 245 (2020). https://doi.org:10.1186/s13059-020-02134-9
26 Zhao, H. *et al.* CrossMap: a versatile tool for coordinate conversion between genome assemblies. *Bioinformatics* **30**, 1006-1007 (2014). https://doi.org:10.1093/bioinformatics/btt730
27 Gel, B. & Serra, E. karyoploteR: an R/Bioconductor package to plot customizable genomes displaying arbitrary data. *Bioinformatics* **33**, 3088-3090 (2017). https://doi.org:10.1093/bioinformatics/btx346
28 Talenti, A. & Prendergast, J. nf-LO: A Scalable, Containerized Workflow for Genome-to-Genome Lift Over. *Genome Biol Evol* **13** (2021). https://doi.org:10.1093/gbe/evab183
29 Ren, J. & Chaisson, M. J. P. lra: A long read aligner for sequences and contigs. *PLoS Comput Biol* **17**, e1009078 (2021). https://doi.org:10.1371/journal.pcbi.1009078
30 Li, H. New strategies to improve minimap2 alignment accuracy. *Bioinformatics* **37**, 4572-4574 (2021). https://doi.org:10.1093/bioinformatics/btab705
31 Langmead, B. & Salzberg, S. L. Fast gapped-read alignment with Bowtie 2. *Nat Methods* **9**, 357-359 (2012). https://doi.org:10.1038/nmeth.1923
32 Shumate, A. & Salzberg, S. L. Liftoff: accurate mapping of gene annotations. *Bioinformatics* **37**, 1639-1643 (2021). https://doi.org:10.1093/bioinformatics/btaa1016
33 Hoff, K. J., Lomsadze, A., Borodovsky, M. & Stanke, M. Whole-Genome Annotation with BRAKER. *Methods Mol Biol* **1962**, 65-95 (2019). https://doi.org:10.1007/978-1-4939-9173-0_5
34 Stanke, M. *et al.* AUGUSTUS: ab initio prediction of alternative transcripts. *Nucleic Acids Res* **34**, W435-439 (2006). https://doi.org:10.1093/nar/gkl200
35 Haas, B. J. *et al.* Automated eukaryotic gene structure annotation using EVidenceModeler and the Program to Assemble Spliced Alignments. *Genome Biol* **9**, R7 (2008). https://doi.org:10.1186/gb-2008-9-1-r7
36 Edgar, R. C. MUSCLE: multiple sequence alignment with high accuracy and high throughput. *Nucleic Acids Res* **32**, 1792-1797 (2004). https://doi.org:10.1093/nar/gkh340
37 Kumar, S., Stecher, G. & Tamura, K. MEGA7: Molecular Evolutionary Genetics Analysis Version 7.0 for Bigger Datasets. *Mol Biol Evol* **33**, 1870-1874 (2016). https://doi.org:10.1093/molbev/msw054
38 Bzikadze, A. V. & Pevzner, P. A. TandemAligner: a new parameter-free framework for fast sequence alignment. *bioRxiv*, 2022.2009. 2015.507041 (2022).

39 Krumsiek, J., Arnold, R. & Rattei, T. Gepard: a rapid and sensitive tool for creating dotplots on genome scale. *Bioinformatics* **23**, 1026-1028 (2007). https://doi.org:10.1093/bioinformatics/btm039
