# Supplementary material for: The complete and fully-phased diploid genome of a male Han Chinese
Source: Cell Res. 2023 Jul 14;33(10):745–61. doi: 10.1038/s41422-023-00849-5 (PMC10542383; doi:10.1038/s41422-023-00849-5)
Supplement: Supplementary file 2 — Supplementary information, Figures [file 41422_2023_849_MOESM2_ESM.pdf]

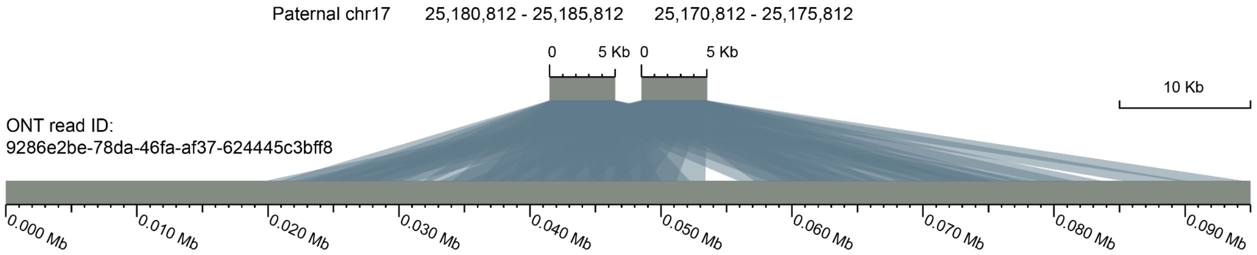

Supplementary information, Fig. S1. Demonstration of a 5 Kb gap that is filled with ONT UL reads in paternal chr17.

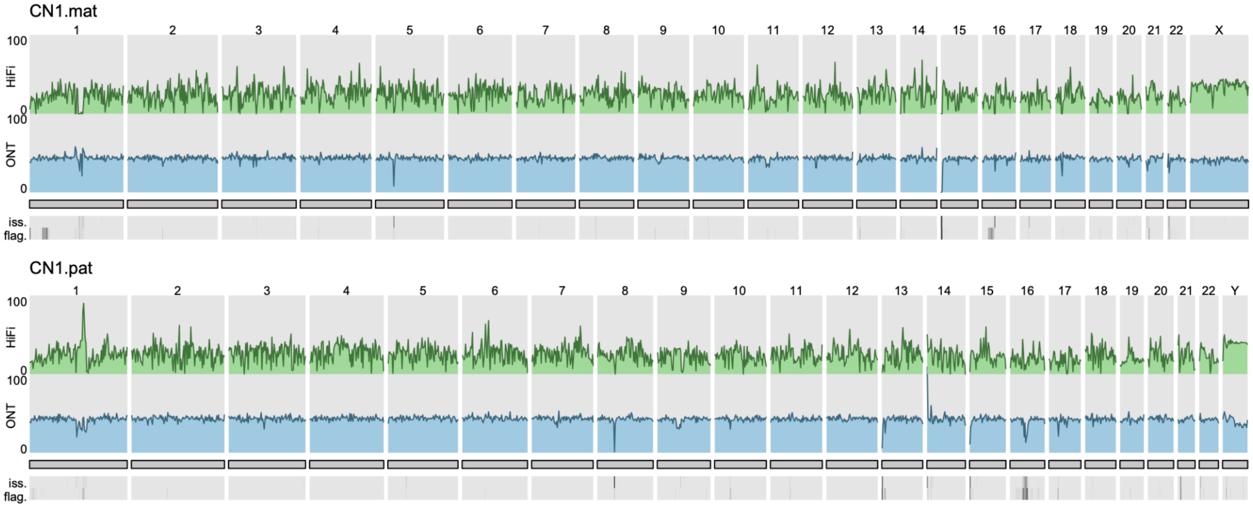

Supplementary information, Fig. S2. **Sequence coverage and validation of CN1 diploid genome**. Coverage of binned HiFi and ONT reads along the CN1 diploid genome.

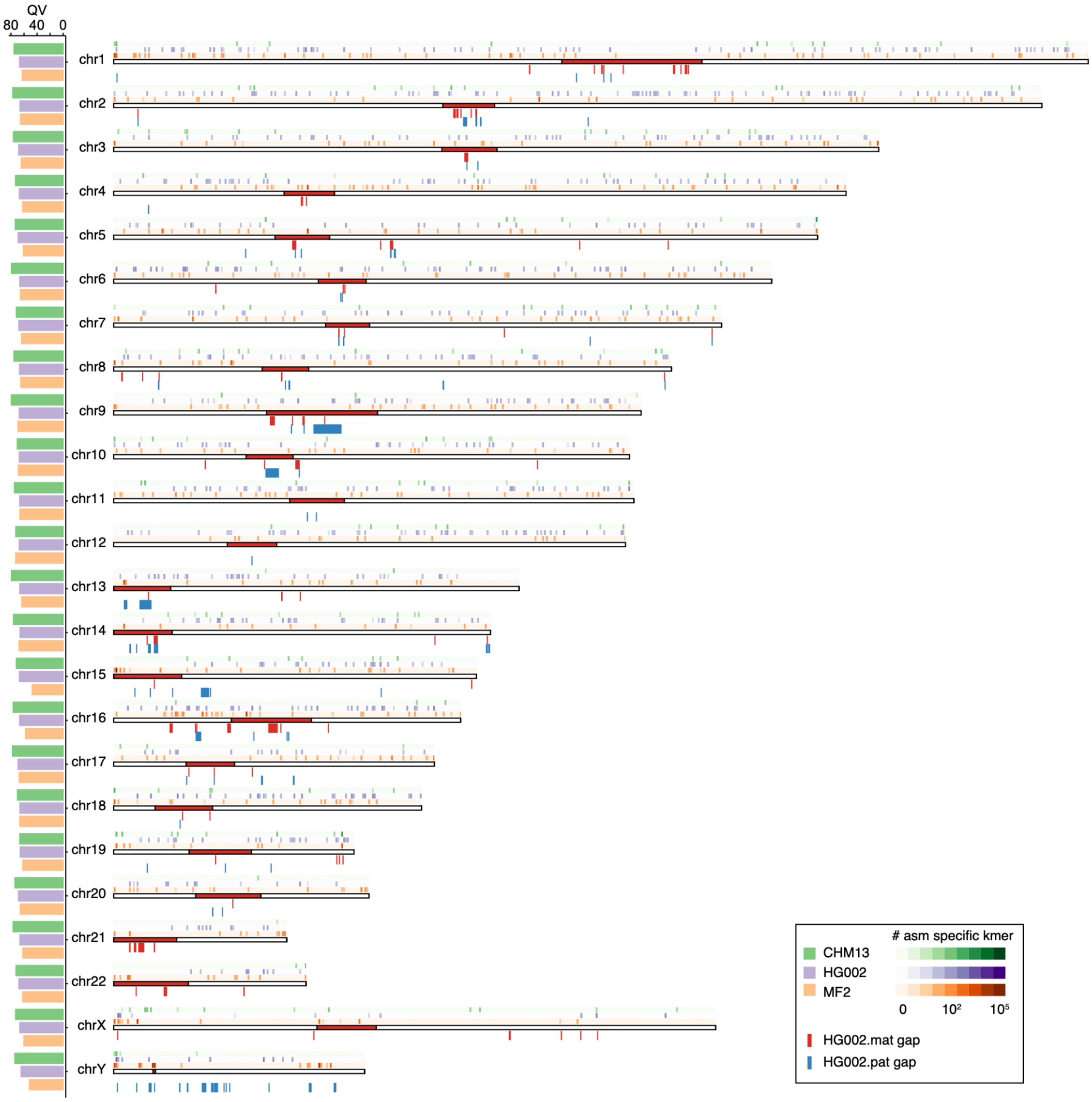

Supplementary information, Fig. S3. QV and assembly-only kmer distribution along each chromosome in CHM13v2.0, HG002 (combined) and CN1v0.8.1 (combined). The coordinates of HG002 gaps in CN1 are also plotted along each chromosome.

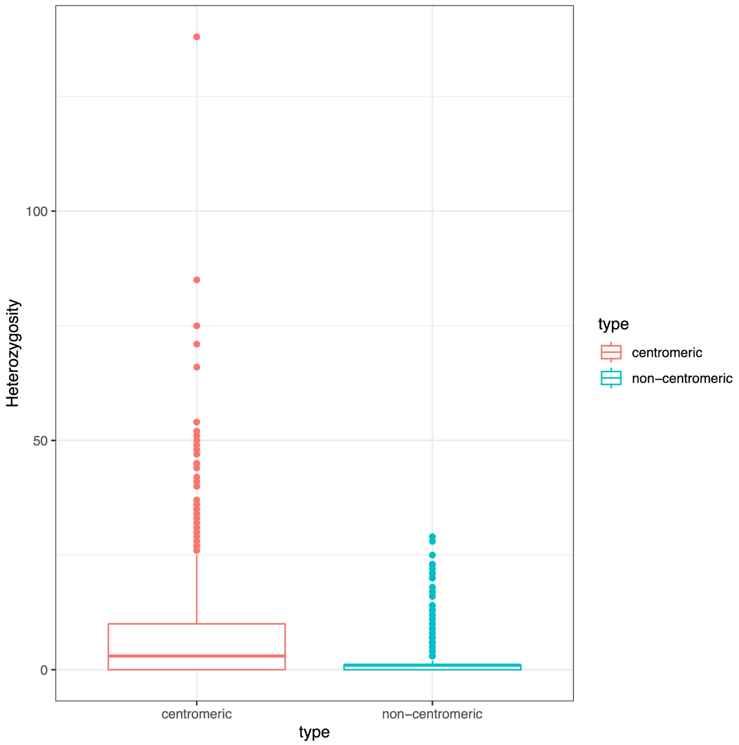

Supplementary information, Fig. S4. Heterozygosity comparison between centromeric and non-centromeric regions. The heterozygosity rate was calculated as the SV count in each 500 kb window.

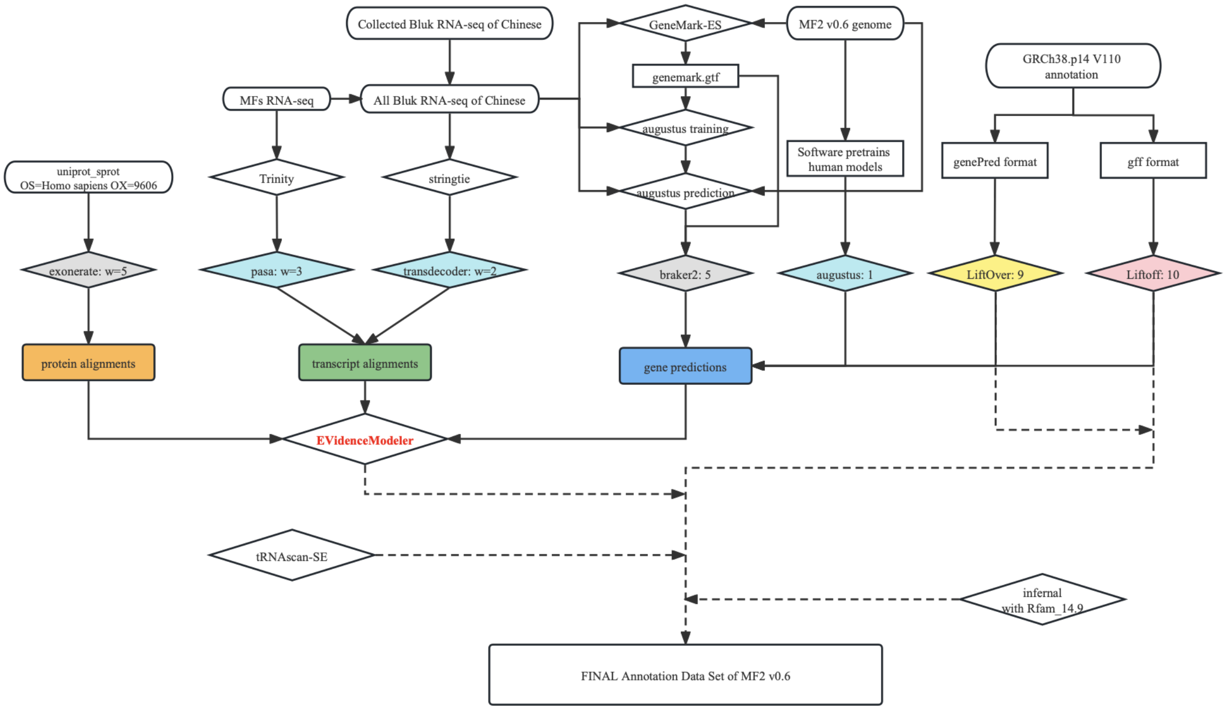

Supplementary information, Fig. S5. The annotation pipeline for CN1 genome.

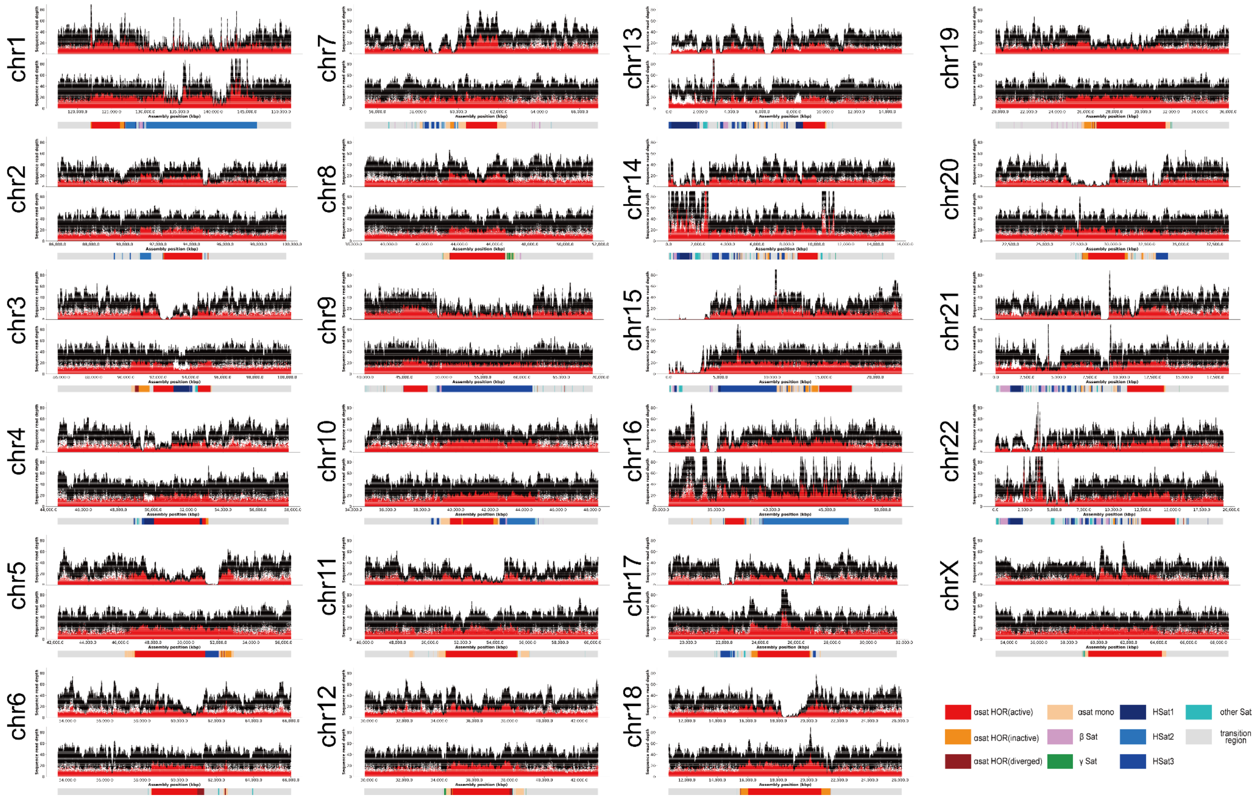

Supplementary information, Fig. S6. NucFreq plot of the ONT coverage of CN1 maternal centromere region. For each chromosome, the top track is the coverage generated by veritymap, the bottom track is the coverage generated by winnowmap, and the colored bars are the annotation of the centromere satellites. The performance of winnowmap is much better than the veritymap.

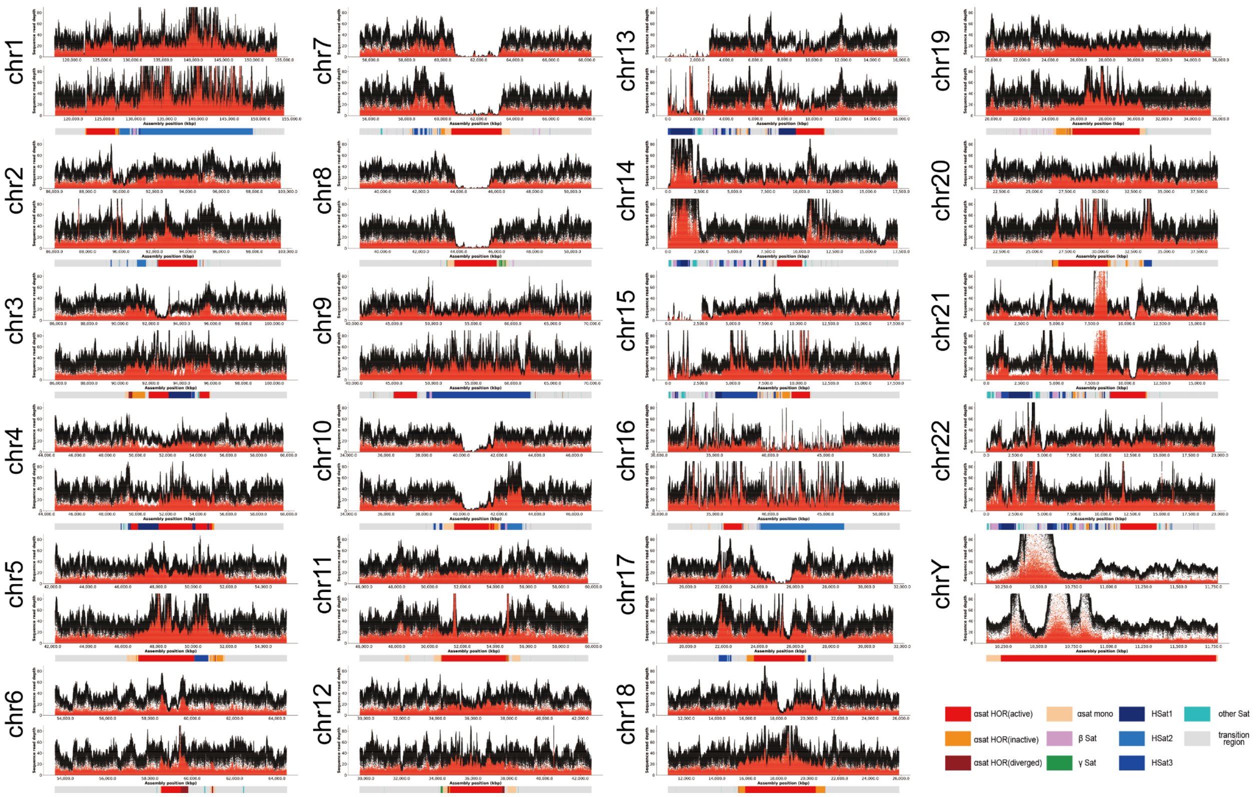

Supplementary information, Fig. S7. NucFreq plot of the ONT coverage of CN1 paternal centromere region. For each chromosome, the top track is the coverage generated by veritymap, the bottom track is the coverage generated by winnowmap, and the colored bars are the annotation of the centromere satellites. The performance of winnowmap is much better than the veritymap.

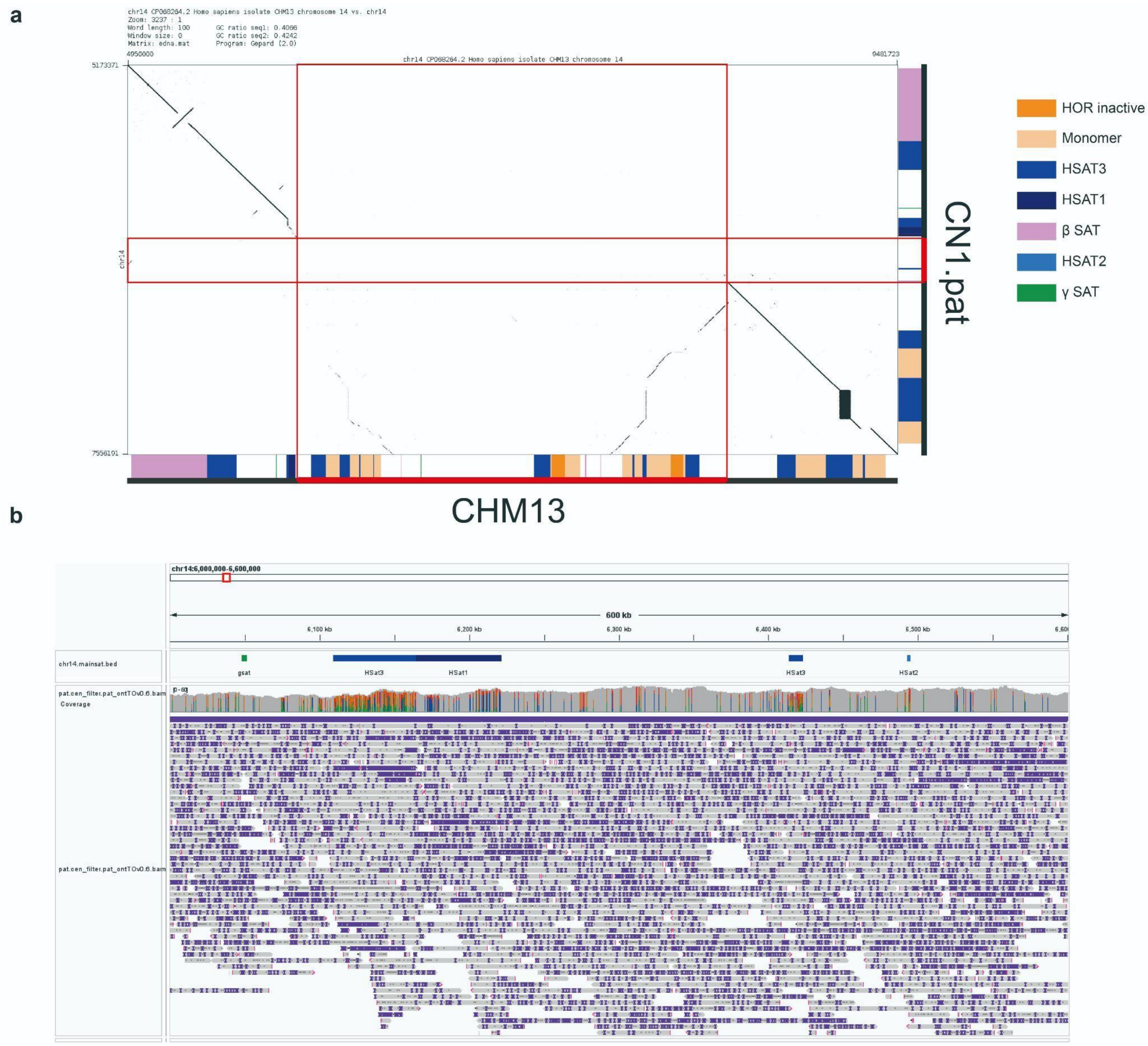

Supplementary information, Fig. S8. Validation of the absence of inactive HOR in CN1 paternal chr14

a, The dotplot (drawn by gepard (v2.1)[39] with word length 100) shows that the 2.46 Mb array containing an inactive HOR in CHM13 chromosome 14 is substituted in 294 Kb sequence in CN1.pat. This leads to the absence of the inactive HOR array and the emergence of HSat2 in CN1.pat.

b, Zooming in the red line region in CN1 paternal chromosome 14 illustrates that the inactive HOR absence and emergence of HSat2 in CN1.pat is not an assembly issue.

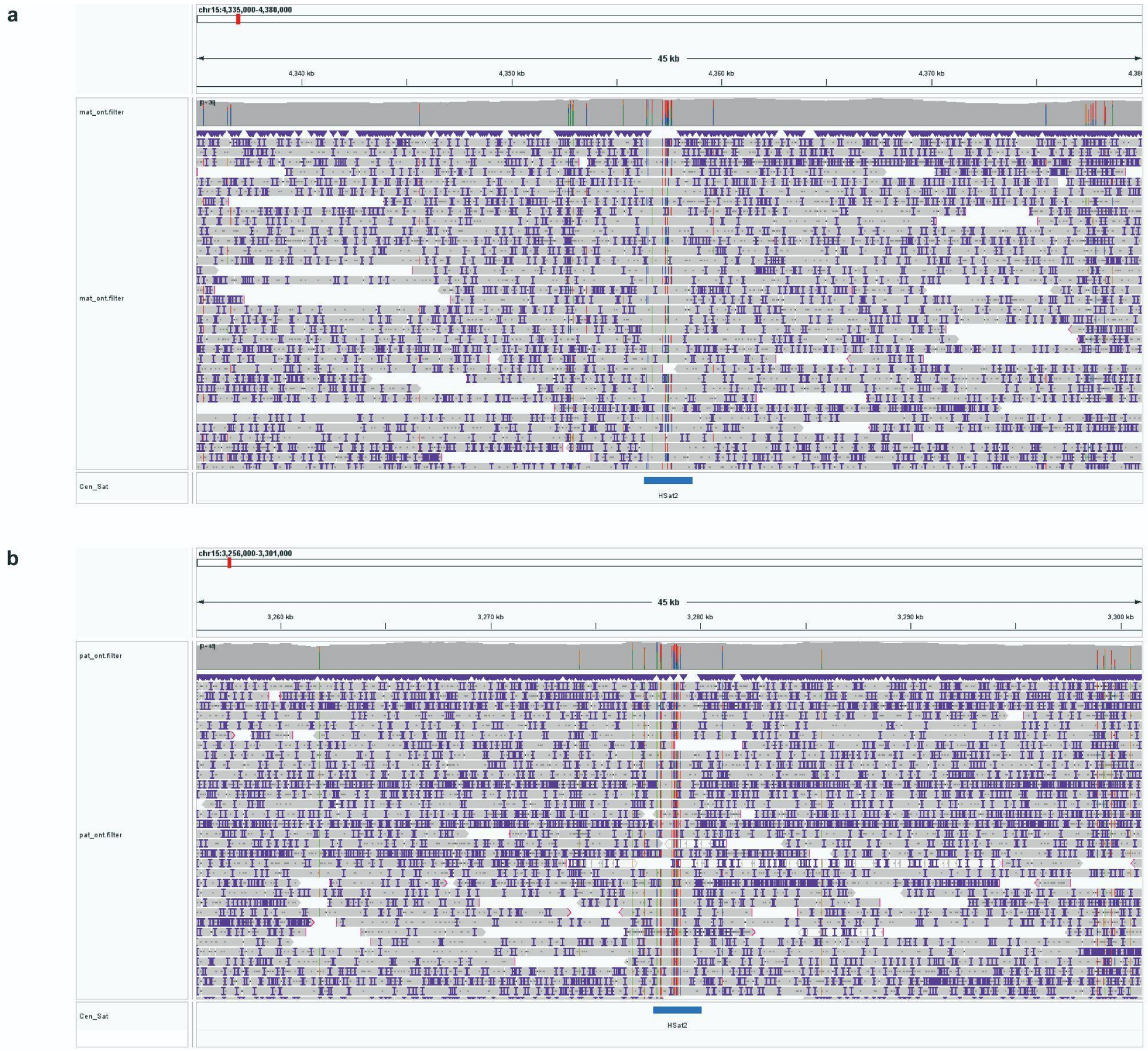

Supplementary information, Fig. S9. An additional ~2 Kb HSat2 in chromosome 15 of CN1.mat (a) and CN1.pat (b) can be verified by the phased ONT reads.

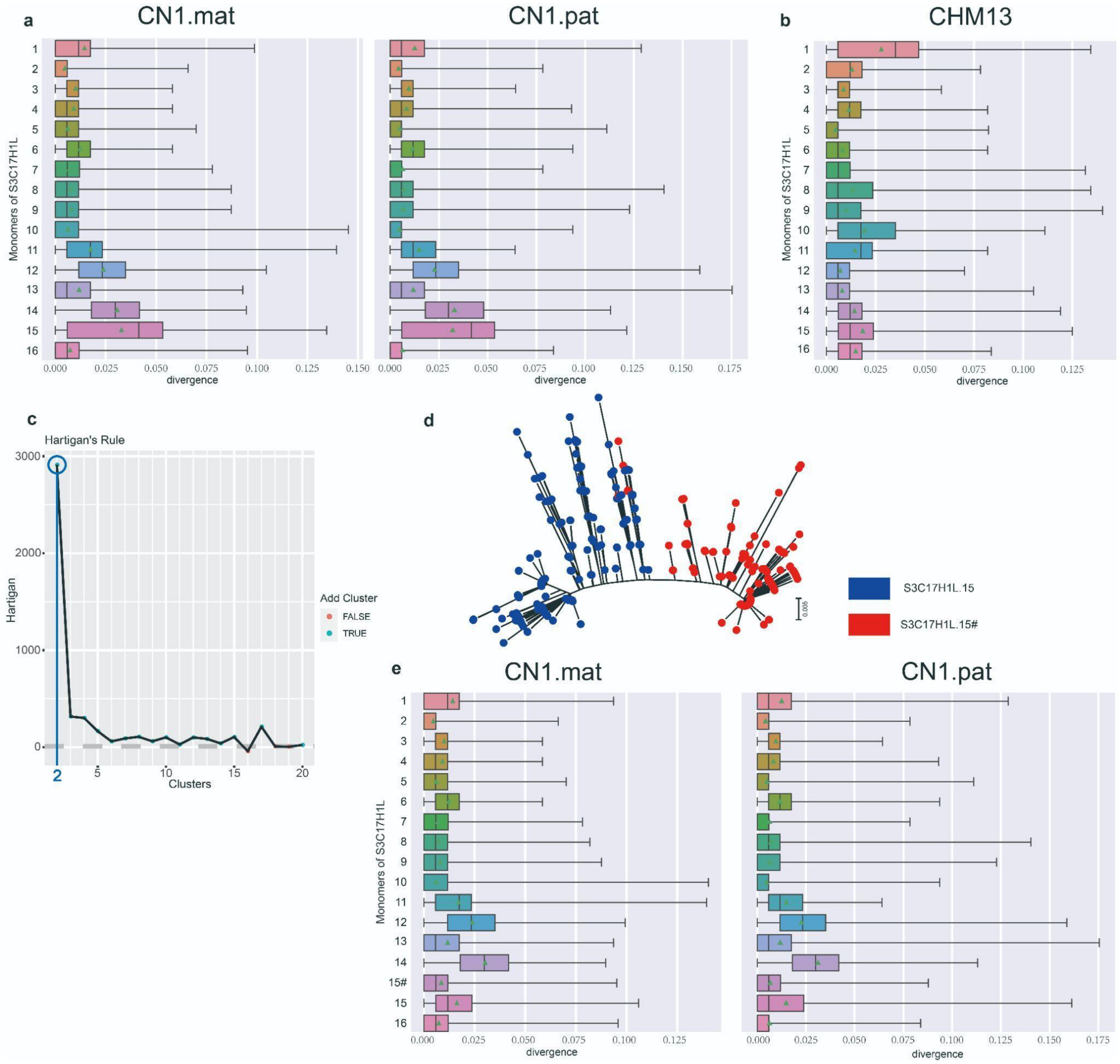

Supplementary information, Fig. S10. Classification and validation of the new monomer in CN1 chr17.

a, **b**, Boxplot of pairwise divergences of each type of monomer in chromosome 17 of CHM13 and CN1. Both CN1.mat and CN1.pat S3C17H1L exhibit relatively high intra-monomer divergence compared to that of CHM13, indicating that this type of monomer of CN1 may be further classified into subgroups. Box plots show median (line), mean (triangle), quartiles (boxes), and range (whiskers).

c, Hartigan plot supports that the S3C17H1L.1 monomers can be clustered into 2 groups

d, The phylogenetic tree of the raw S3C17H1L.1 monomers, where different color indicates different subgroups in our clusters.

e, Boxplot of intra-monomer divergences in chromosome 17 after classification.

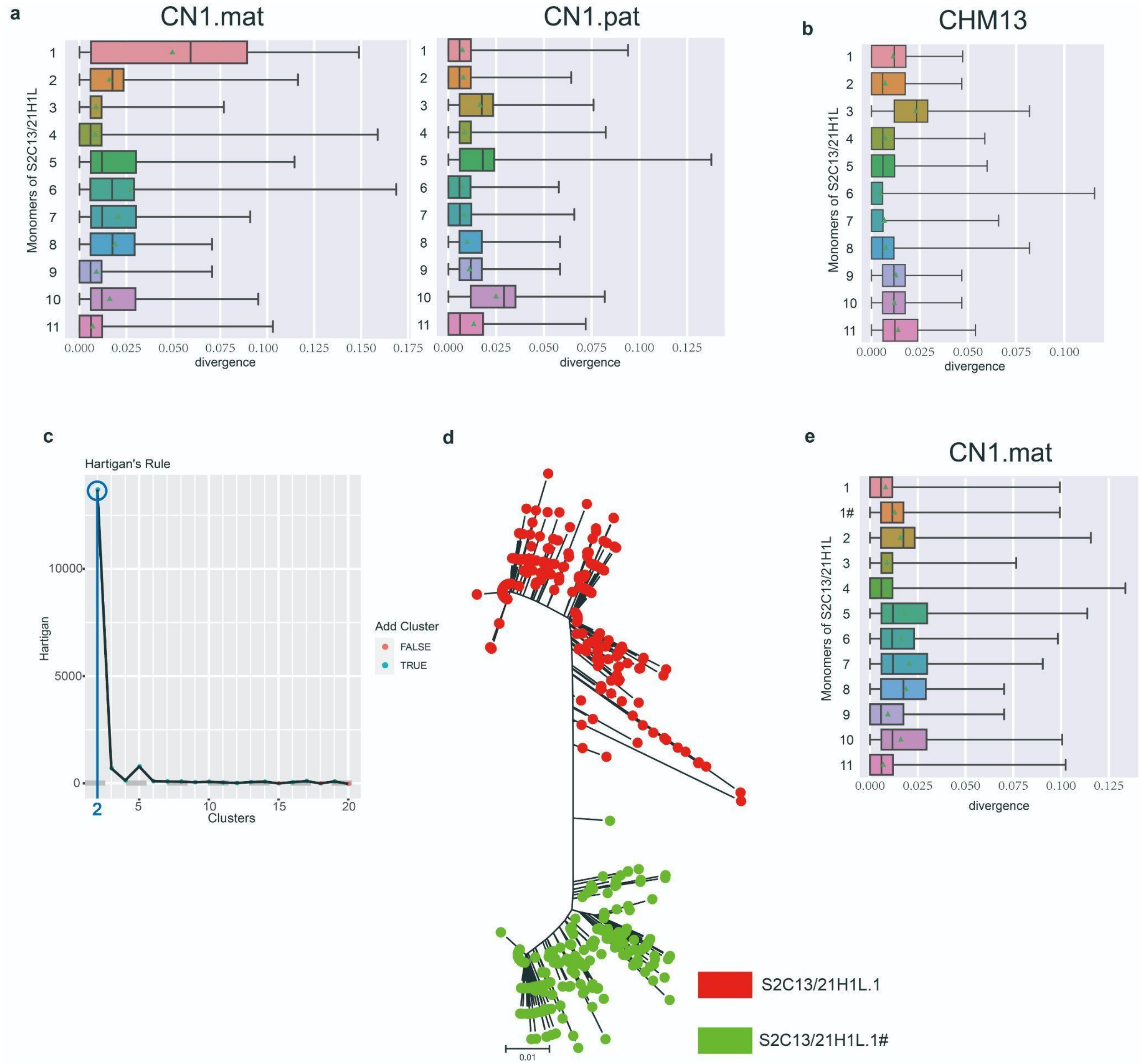

Supplementary information, Fig. S11. Classification and validation of the new monomer in CN1.mat chr21.

a, b, Boxplot of pairwise divergences of each type of monomer in chromosome 21 of CHM13 and CN1. Only CN1.mat raw S2C13/21H1L.1 exhibits relatively high intra-monomer divergence compared to that of CHM13 and CN1.pat, indicating that this type of monomer of CN1.mat can be further classified into subgroups. Box plots show median (line), mean (triangle), quartiles (boxes), and range (whiskers).

c, Hartigan plot supports that the raw S2C13/21H1L.1 monomers can be clustered into 2 groups

d, The phylogenetic tree of the raw S2C13/21H1L.1 monomers, where different color indicates different subgroups in our clusters.

e, Boxplot of intra-monomer divergences of CN1.mat chromosome 21 after new classification.

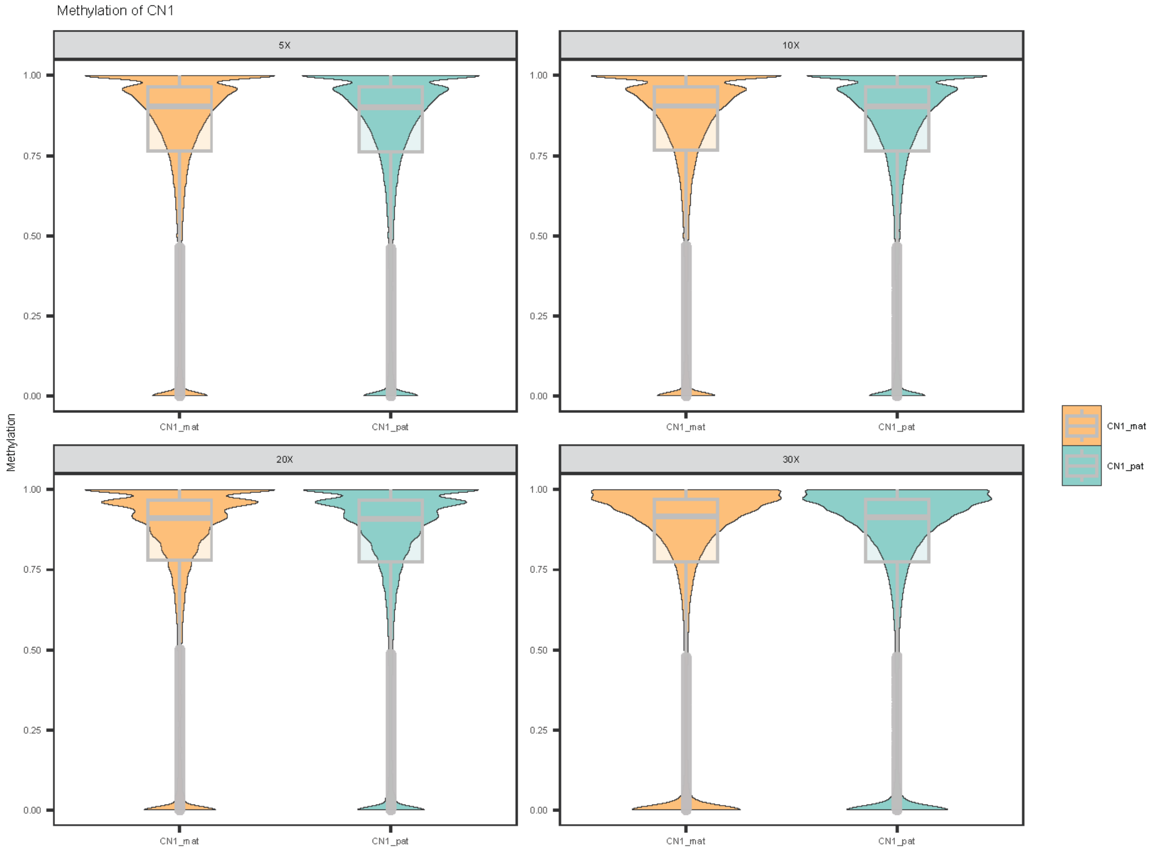

Supplementary information, Fig. S12. Distribution of CpG site methylation level under different depth cutoffs for CN1 diploid genome. CN1_mat and CN1_pat indicate maternal and paternal assembly of CN1, respectively.

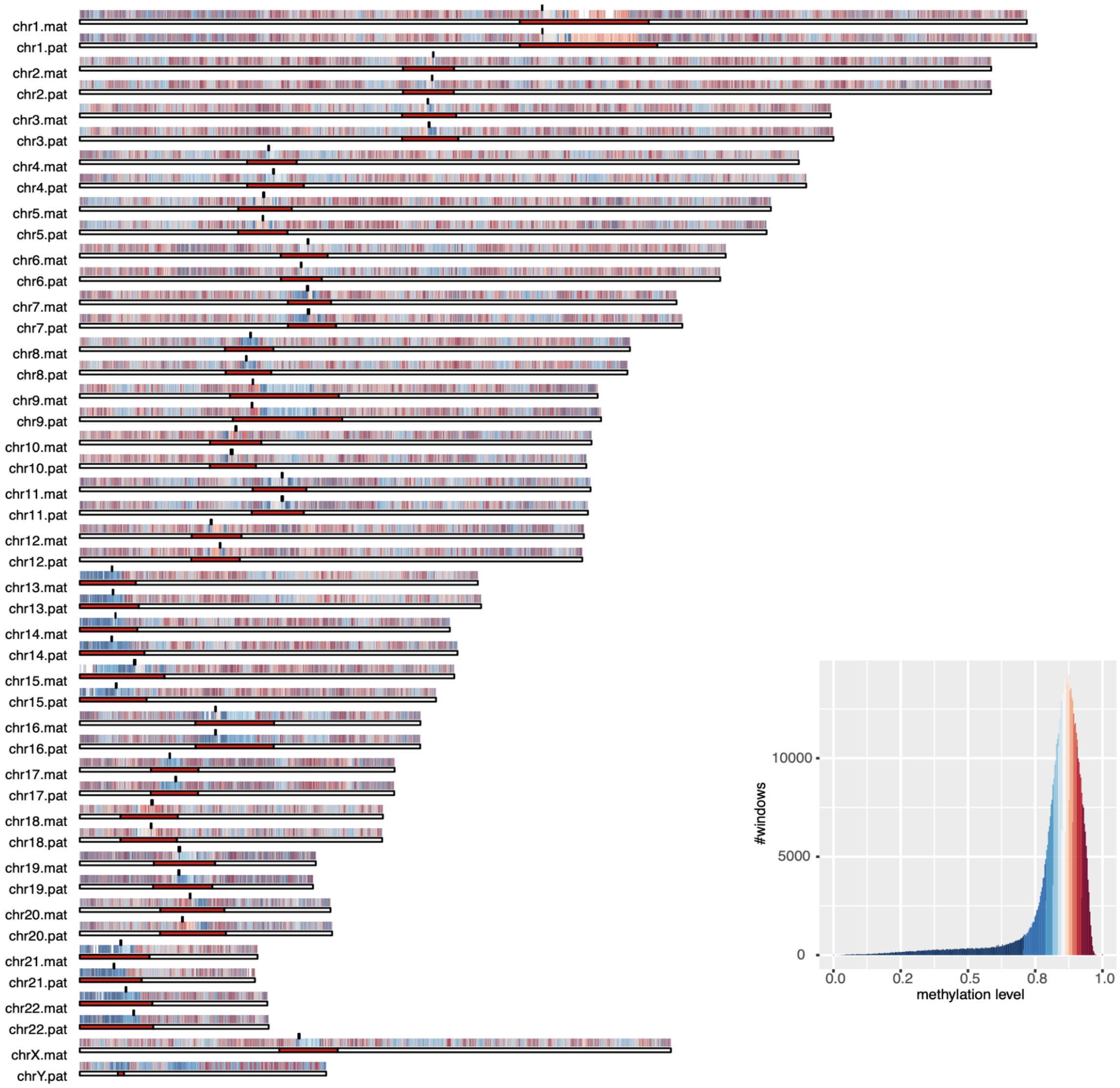

Supplementary information, Fig. S13. Methylation level in 10 Kb windows across CN1 diploid genome. Centromeres and CDRs are marked as red and black blocks, respectively. Chr12 seems to have different CDR coordinates between maternal (first blue region) and paternal (second blue region).

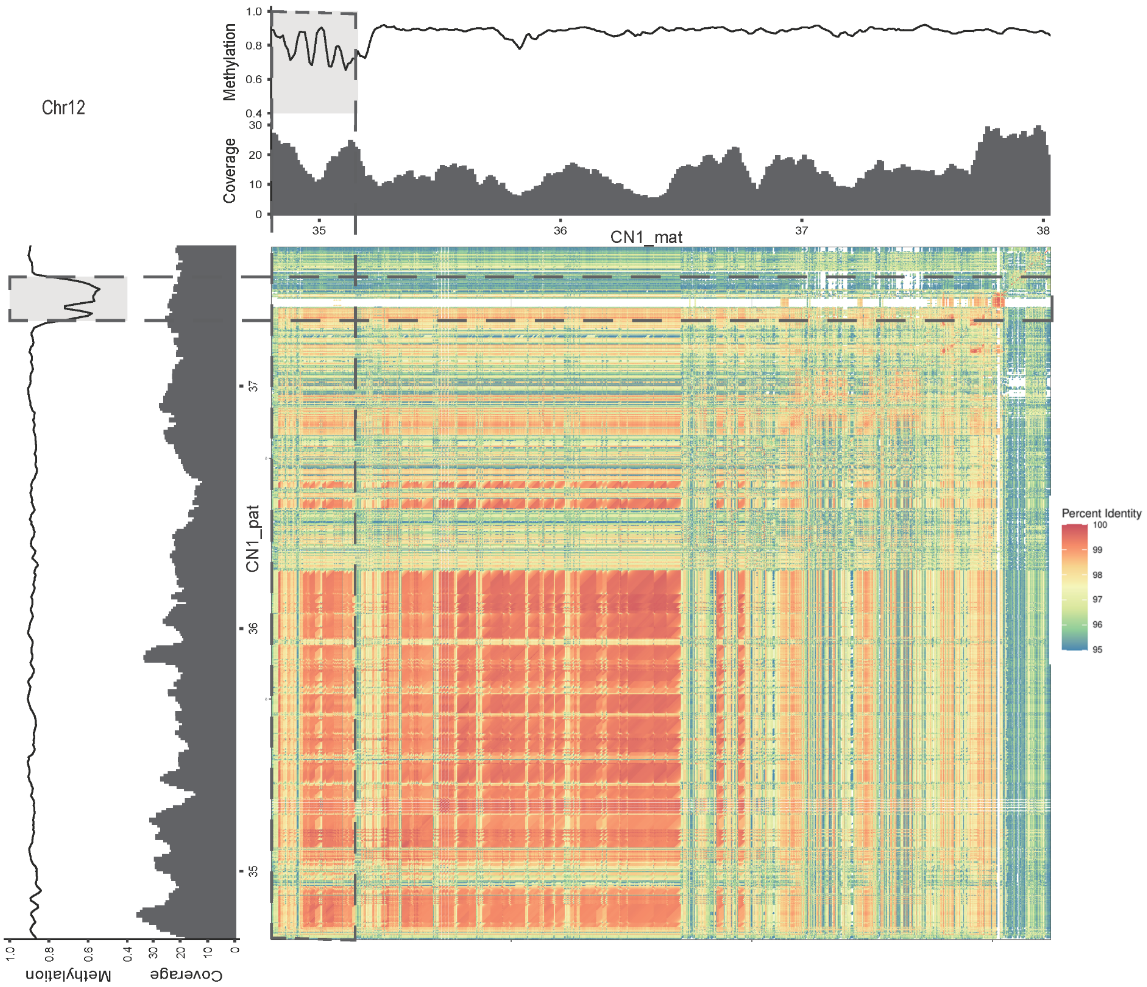

Supplementary information, Fig. S14. Confirmation of CDR coordinates with CDR and its flanking regions in chr12. CDRs are shown in dash-line boxes. Methylation level and corresponding ONT reads depth are plotted along each haploid genome. The color indicates sequence identity.

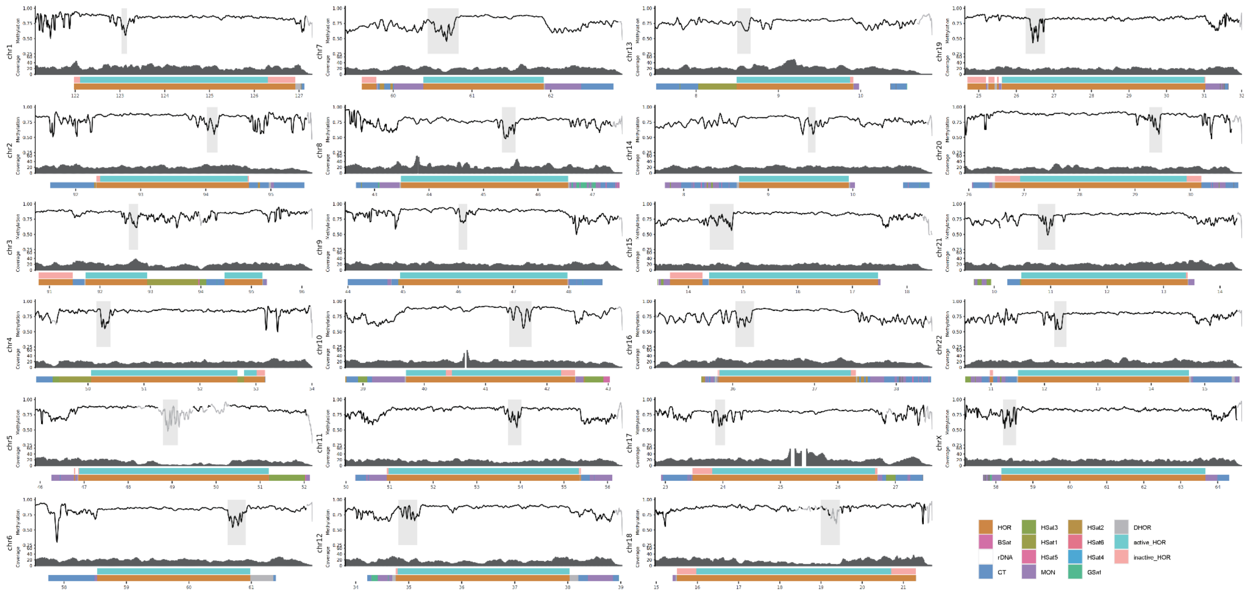

Supplementary information, Fig. S15. Methylation level of CN1.mat CDR and its flanking region. CDRs are shown in the gray shadows. Methylation frequencies estimated with < 5 ONT reads are drawn in gray lines.

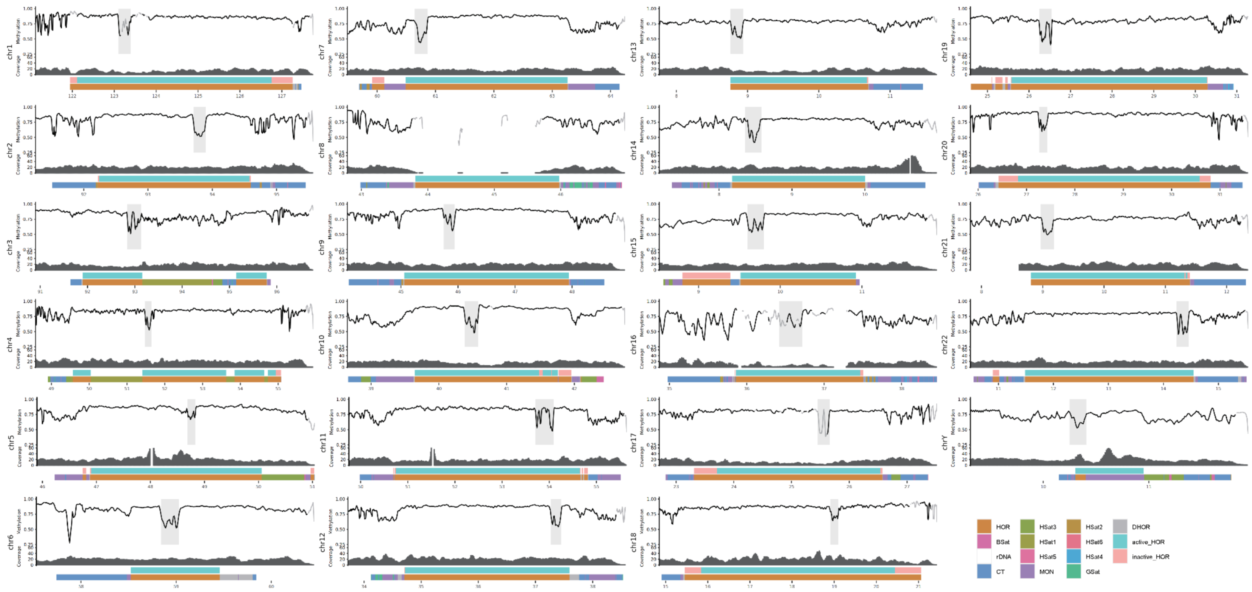

Supplementary information, Fig. S16.Methylation level of CN1.pat CDR and its flanking region. CDRs are shown in the gray shadows. Methylation frequencies estimated with < 5 ONT reads are drawn in gray lines.

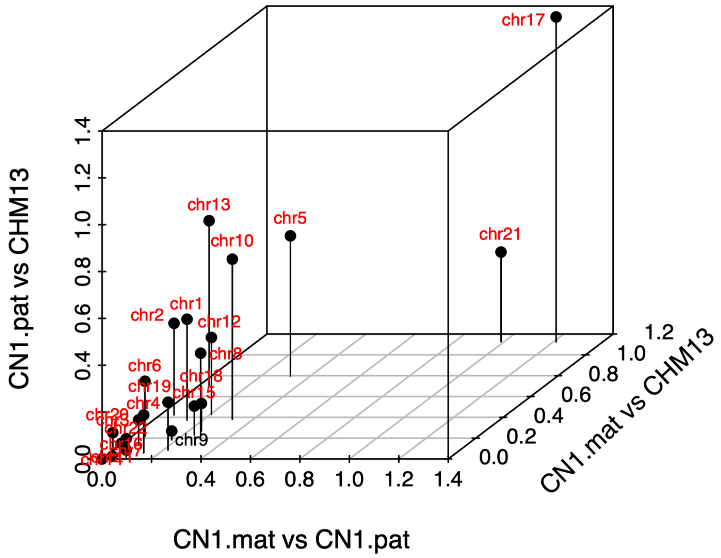

Supplementary information, Fig. S17. CDR HOR composition comparison in CN1.mat, CN1.pat and CHM13. Pairwise euclidean distance is calculated based on the HOR structural variant number ratio in each haploid genome.

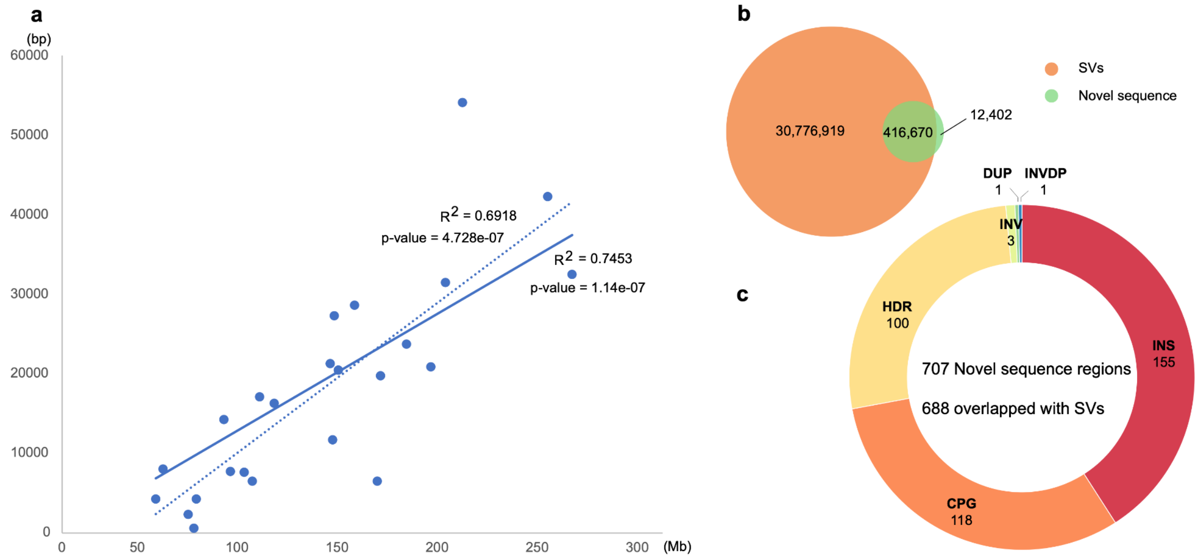

Supplementary information, Fig. S18. The distribution and content of the novel sequence.

a, the correlation between chromosome length and novel sequence length. The dash trendline was fitted using all data while the full line was fitted excluding the outlier (chr3).

b, The intersection between novel sequences and SVs, measured in length.

c, The types of SVs that overlapped with novel sequence regions.

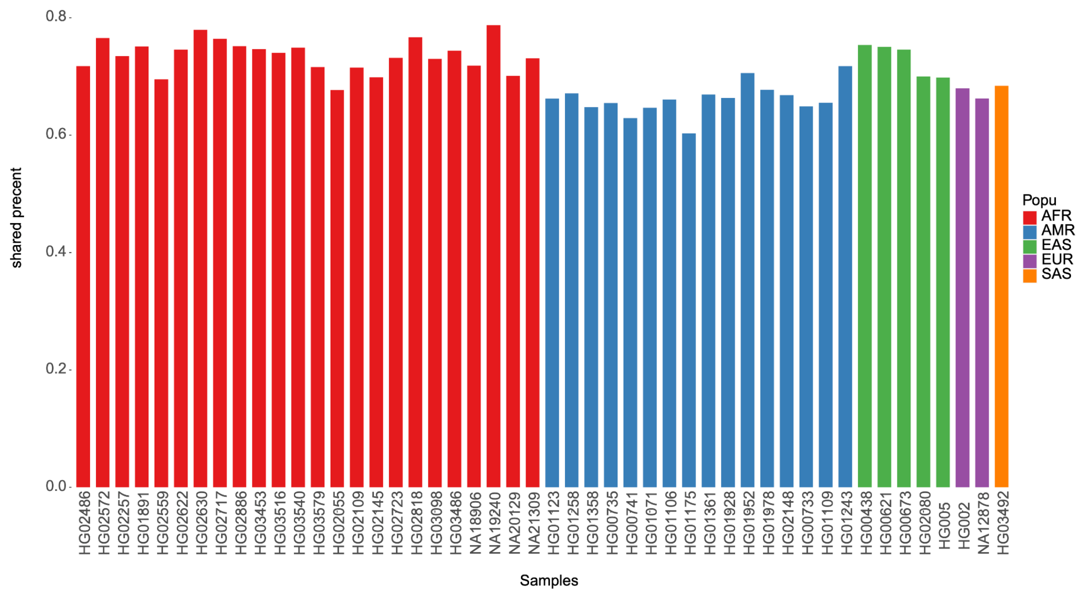

Supplementary information, Fig. S19. The percentage distribution of CN1 novel sequences shared with other individuals in HPRC. NA12878 was downloaded from NCBI (GCA_002077035.3).

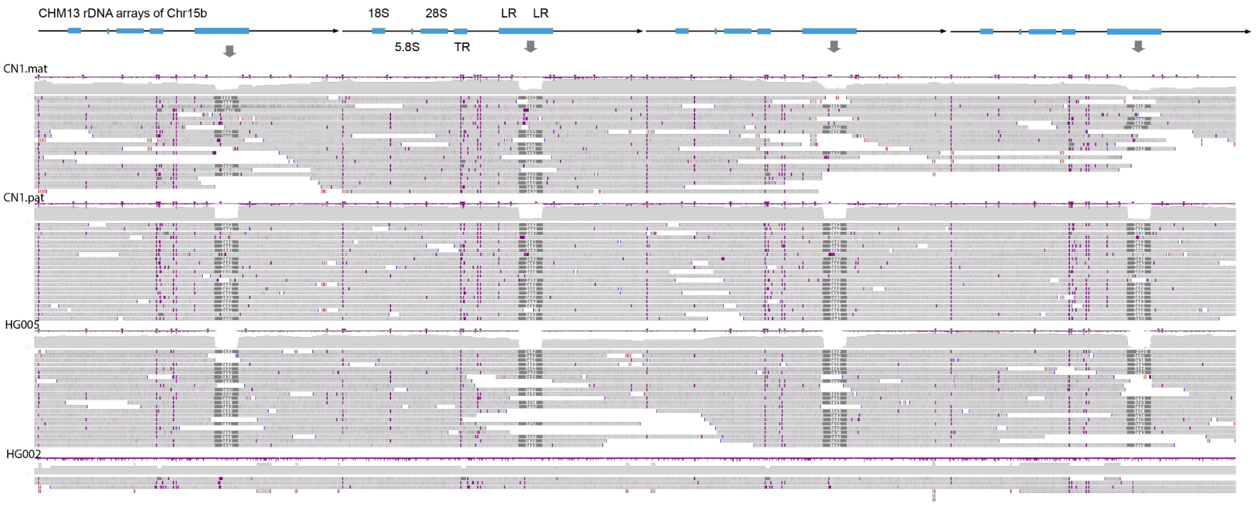

Supplementary information, Fig. S20. Large SV in chr15b model among CHM13, CN1, HG005, and HG002.

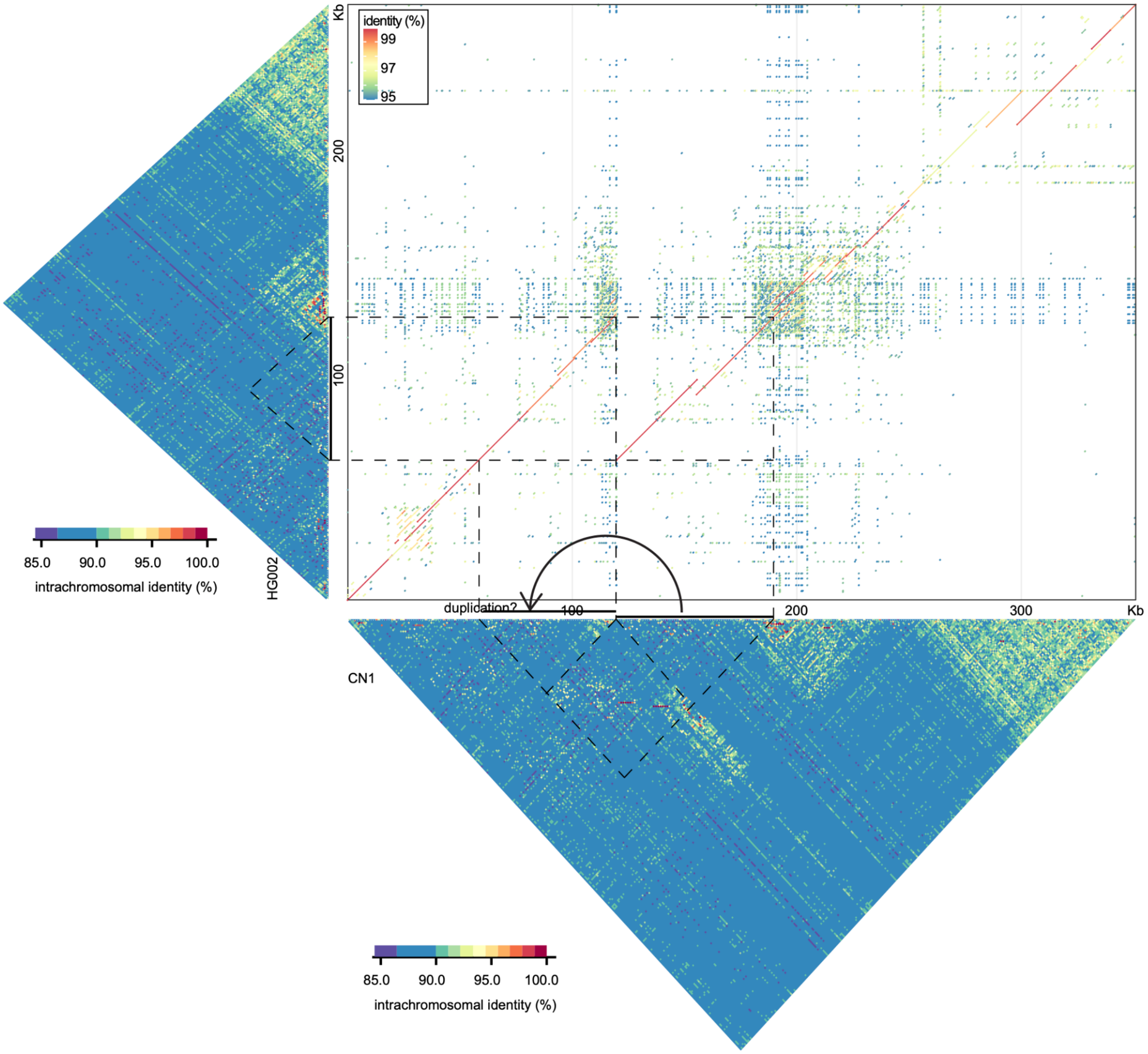

Supplementary information, Fig. S21. Comparison of DYZ19 in CN1 and HG002. A duplication event is likely to happen in CN1, resulting in a ~85 Kb expansion as compared to HG002 and disrupting the intra-array (red block). Regions plotted are: CN1 chrY:21,044,070-21,395,036 and HG002 chrY:20,961,203-21,226,263.

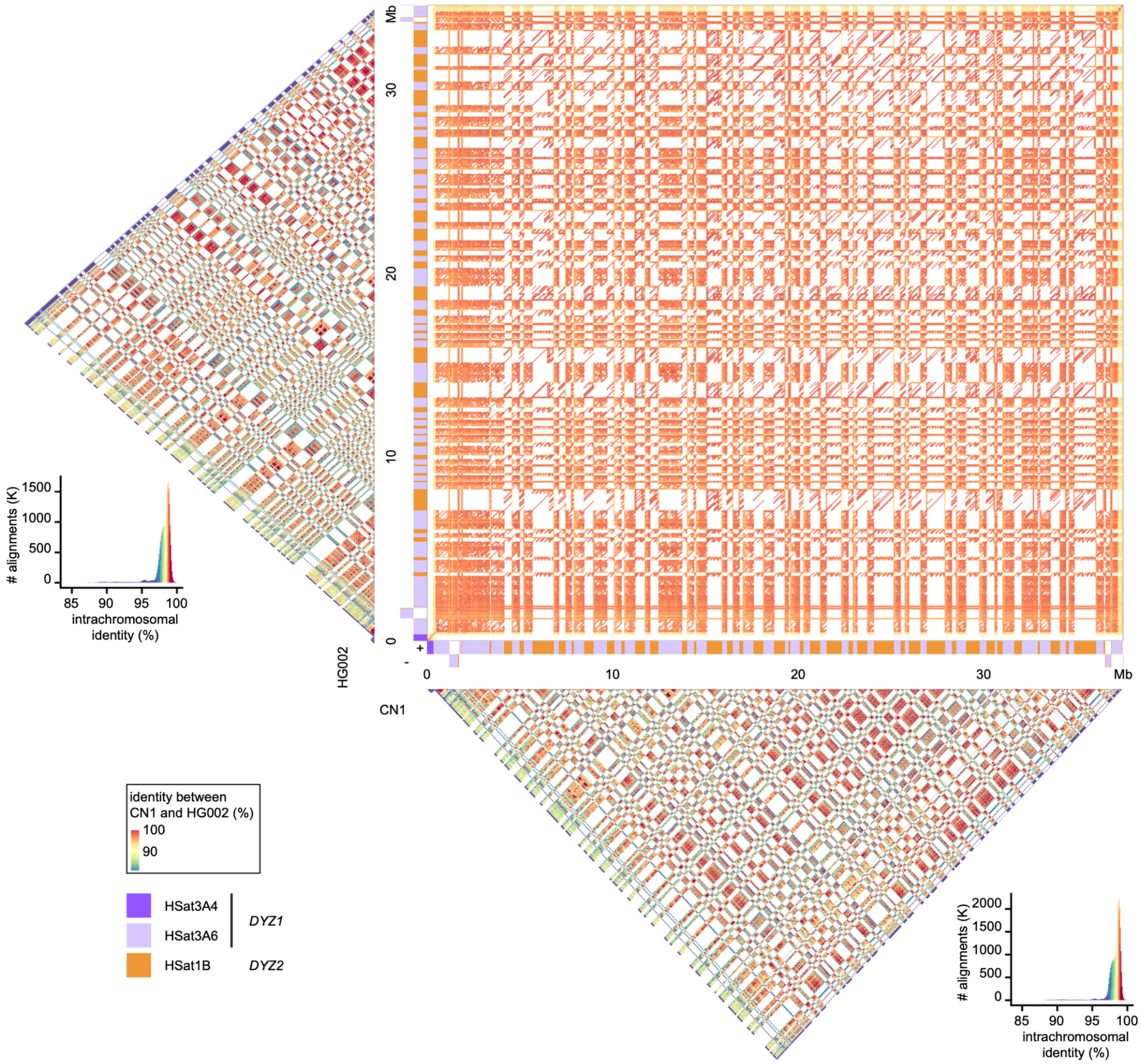

Supplementary information, Fig. S22. Comparison of heterochromatic region in CN1 and HG002. The *DYZ1* and *DYZ2* organization pattern is conserved between the two Y chromosomes. Only DYZ2, i.e. HSat1B, exhibit longer length in CN1 Y compared to HG002 Y. Regions plotted are: CN1 chrY:27,680,339-6,5207,358 and HG002 chrY:27,449,931-62,072,743. Block color indicates type of HSat.

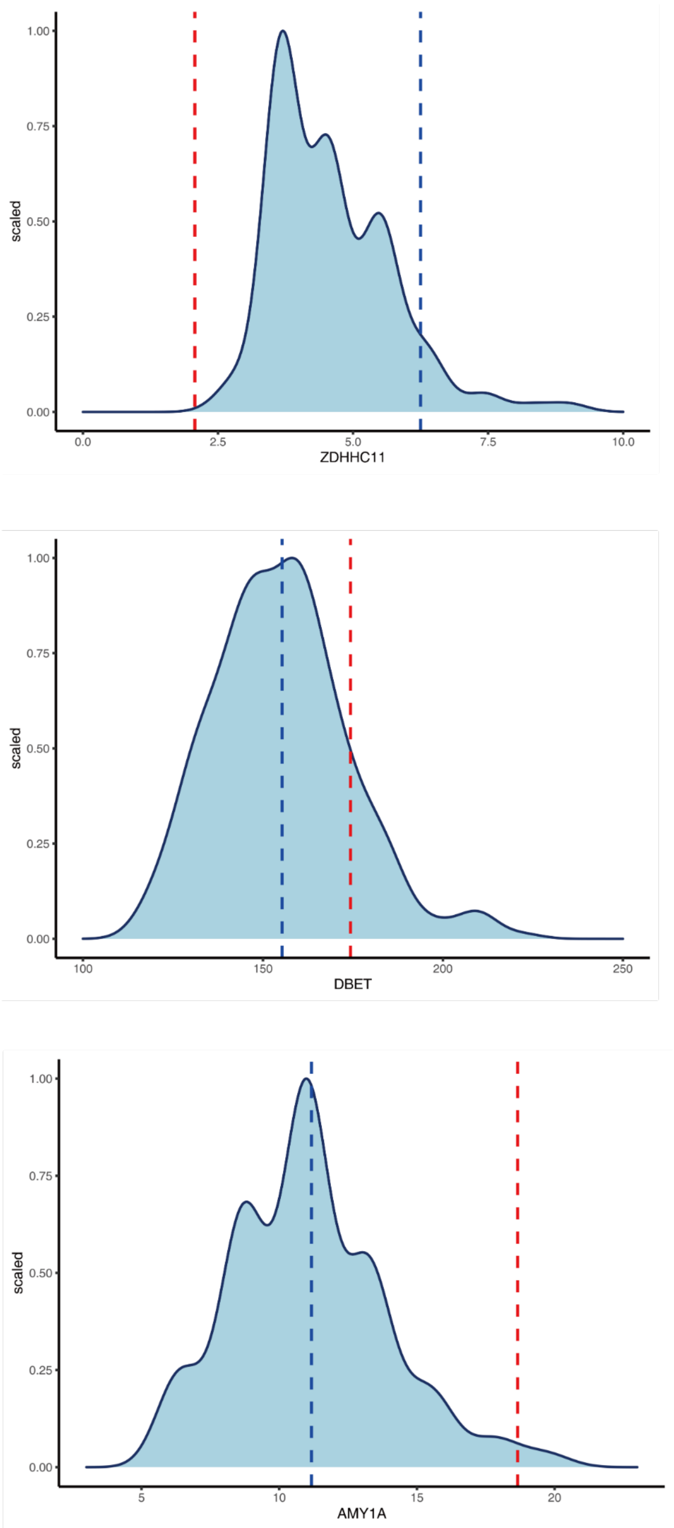

Supplementary information, Fig. S23. Copy number variation among 301 samples in 1KGP (*ZDHHC11*, *DBET* and *AMY1A* as examples). The blue dashed line represents the copy number of the gene on CN1, while the red line represents that on T2T-CHM13.

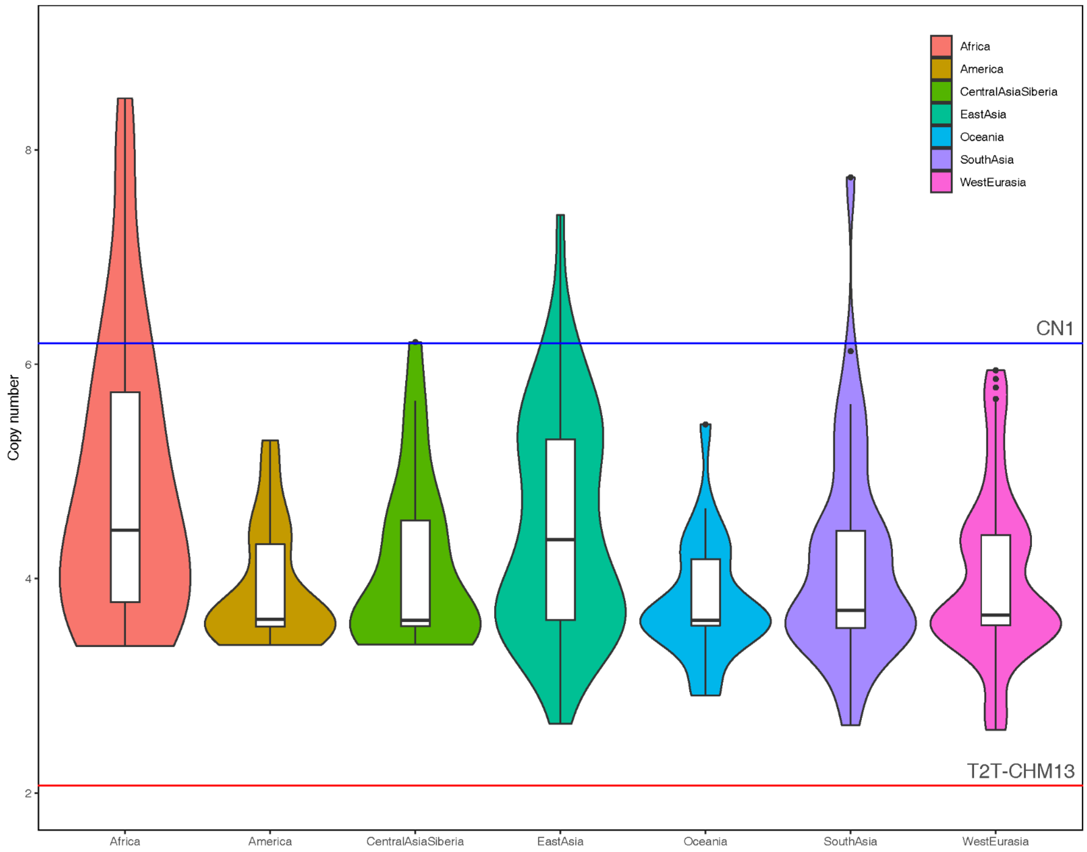

Supplementary information, Fig. S24. *ZDHHC11* copy number distribution of 317 human samples from the Simons Genome Diversity Project. The blue line represents the copy number of *ZDHHC11* on CN1, while the red line represents that on T2T-CHM13. CN1 can better represent the copy number of *ZDHHC11* in most populations, particularly in East Asian, South Asian and African populations.

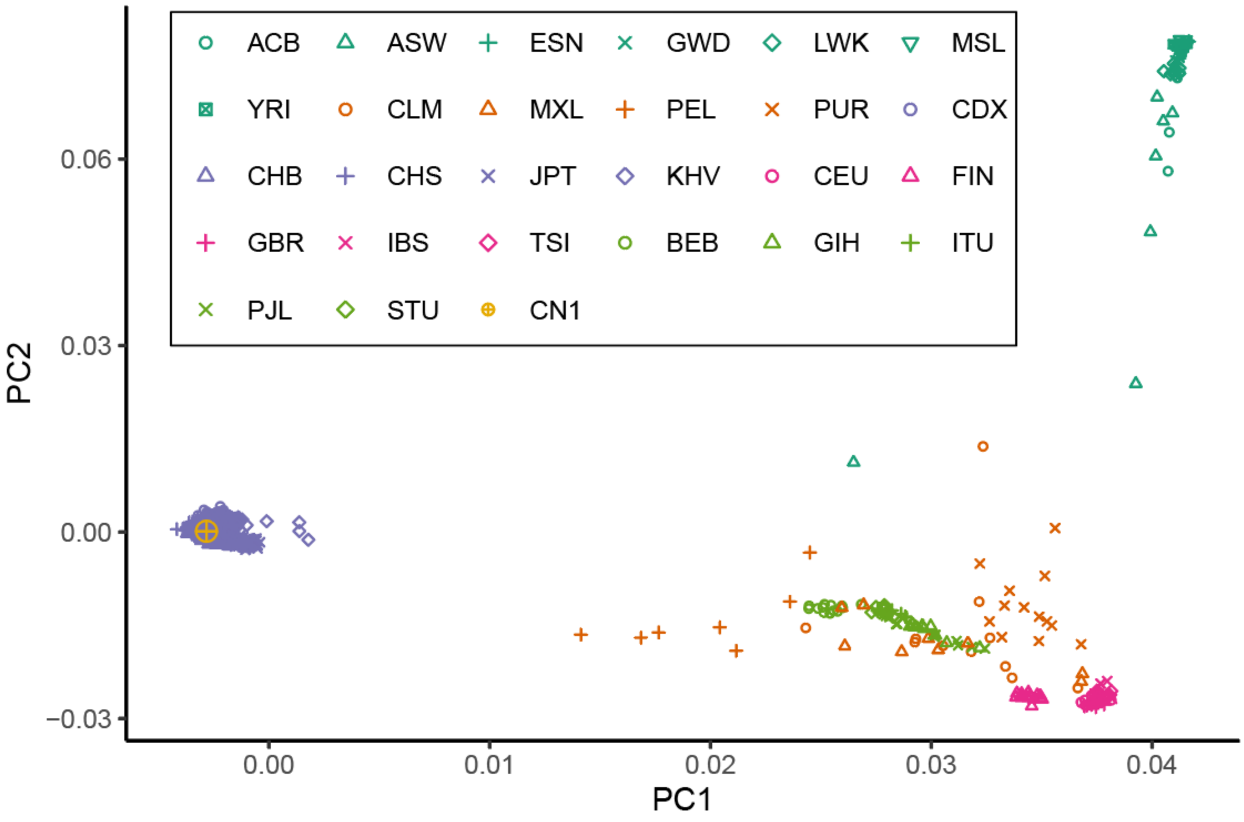

Supplementary information, Fig. S25. PCA of CN1 individual and 1KGP samples. CN1 is located within the East Asian super-population.

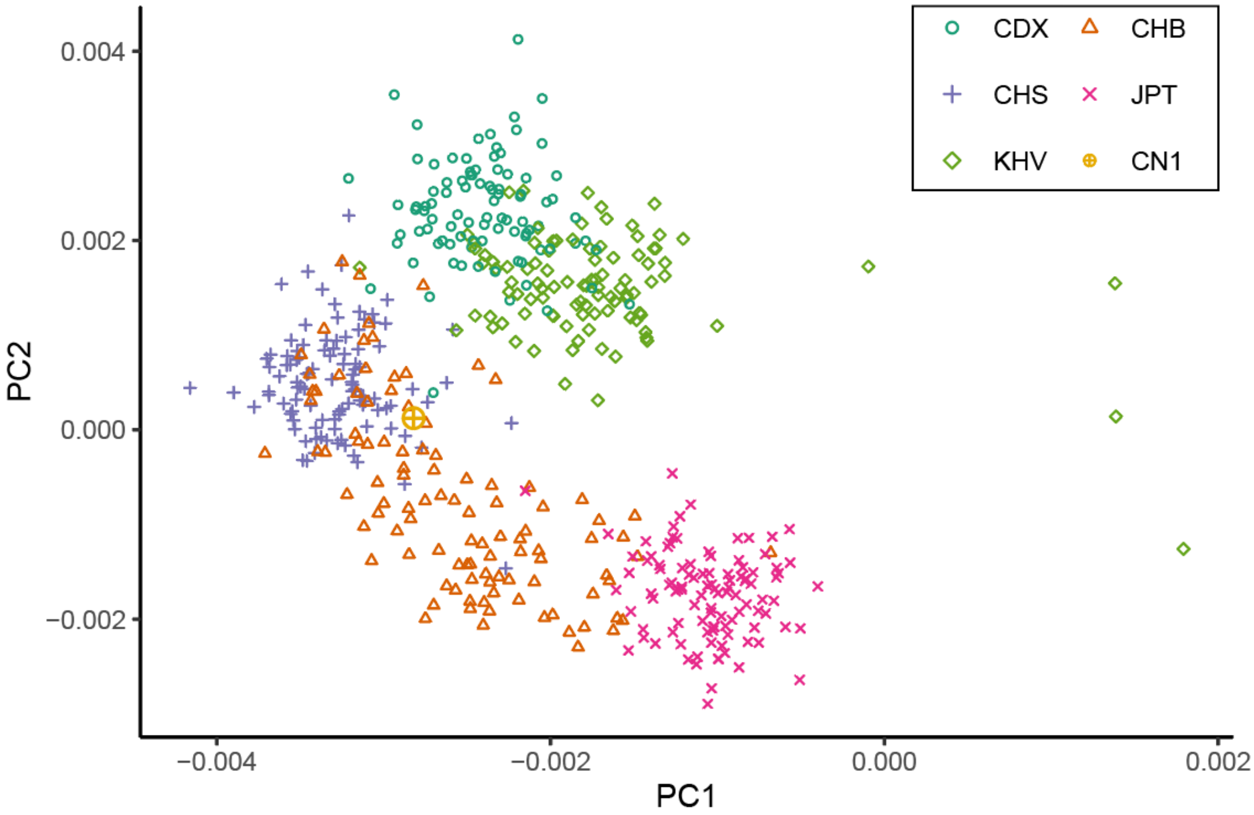

Supplementary information, Fig. S26. PCA of CN1 individual and East Asian samples from 1KGP. CN1 is located within CHS population.

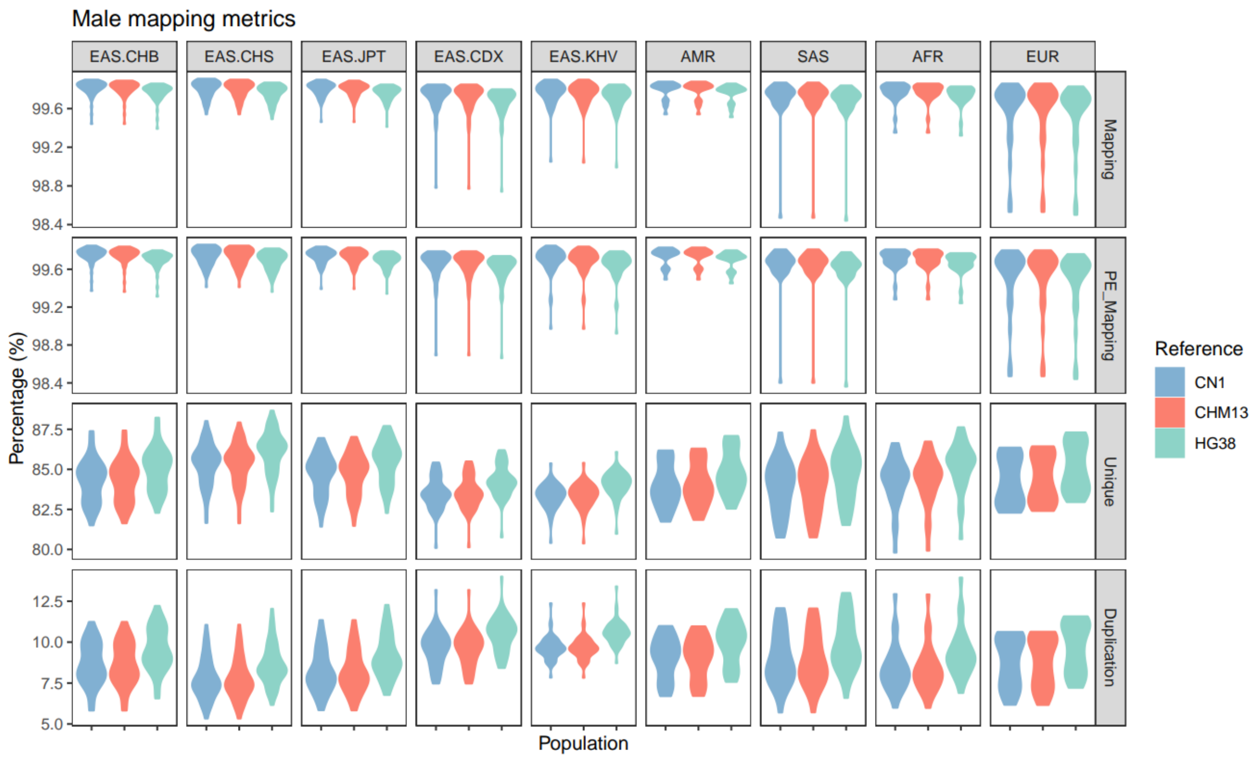

Supplementary information, Fig. S27. Mapping metrics for male individuals in selected 1KGP. Mapping rate, PE mapping rate, uniquely mapped rate and duplication rate was calculated based on raw total reads. Five populations from EAS super-population are unfolded.

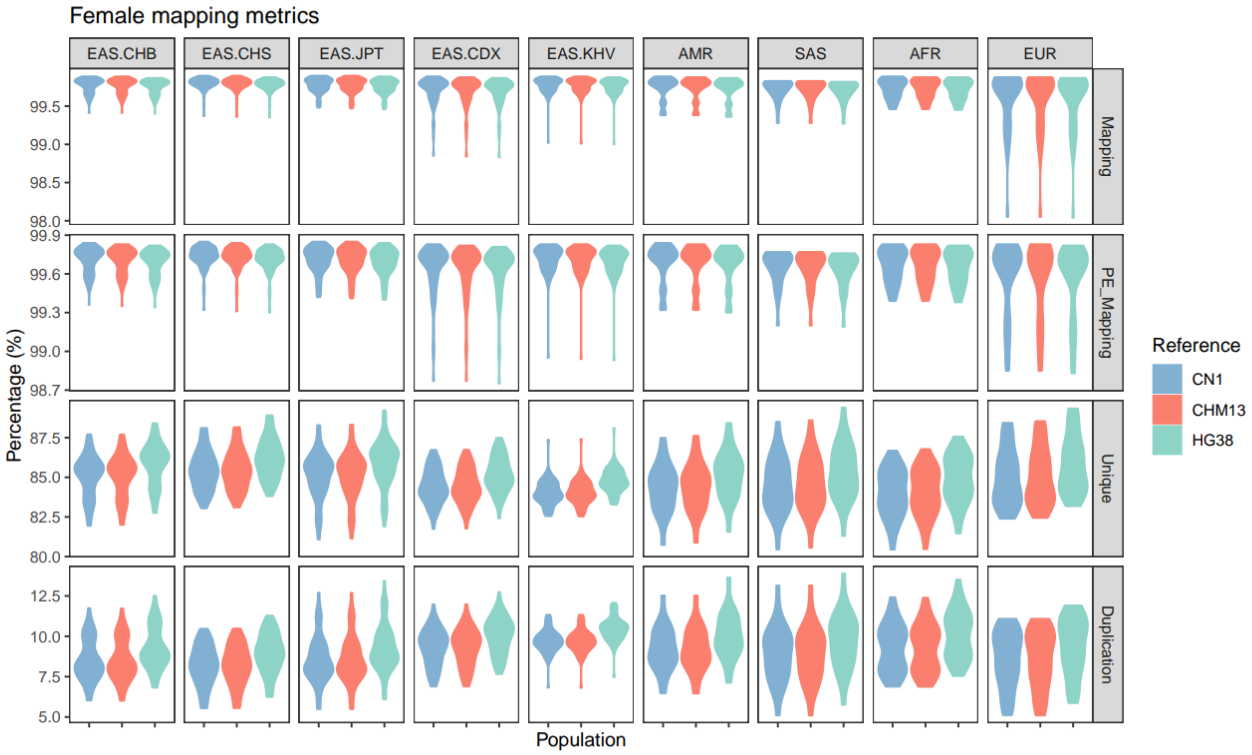

Supplementary information, Fig. S28. Mapping metrics for female individuals in selected 1KGP samples. The same statistics were applied on female individuals.

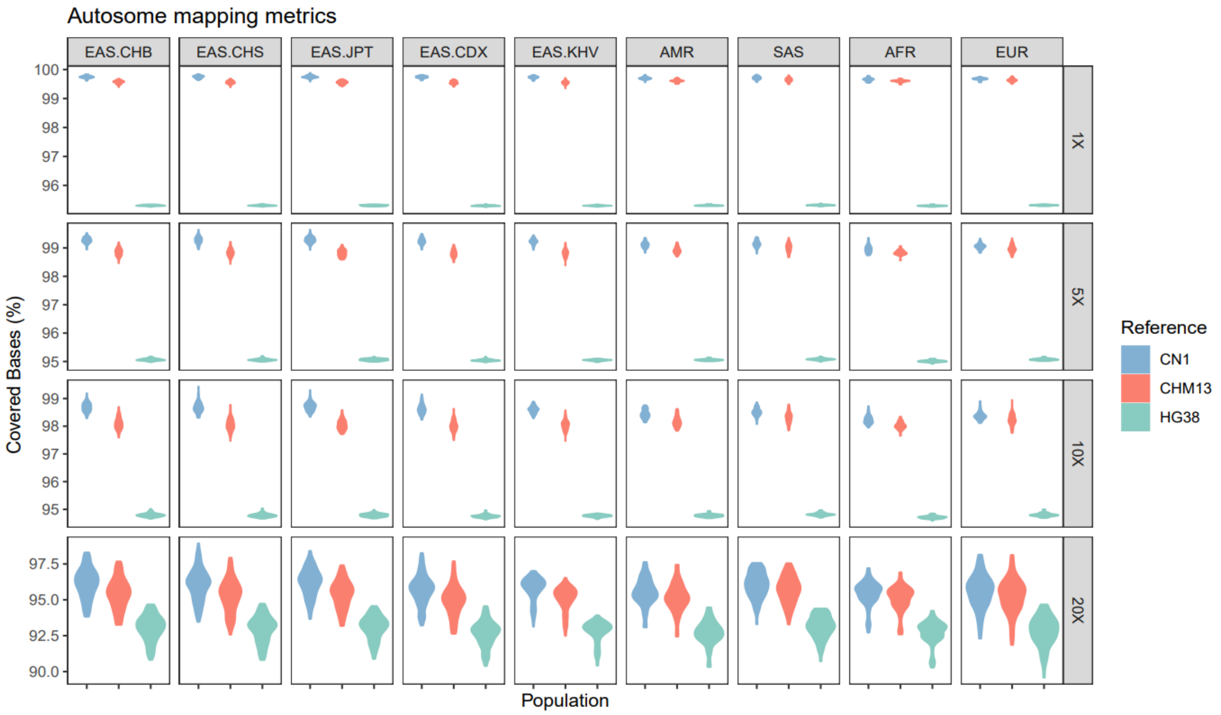

Supplementary information, Fig. S29. The coverage to the autosome reference with different depth of sequencing reads at 1×, 5×, 10×, and 20× reads.

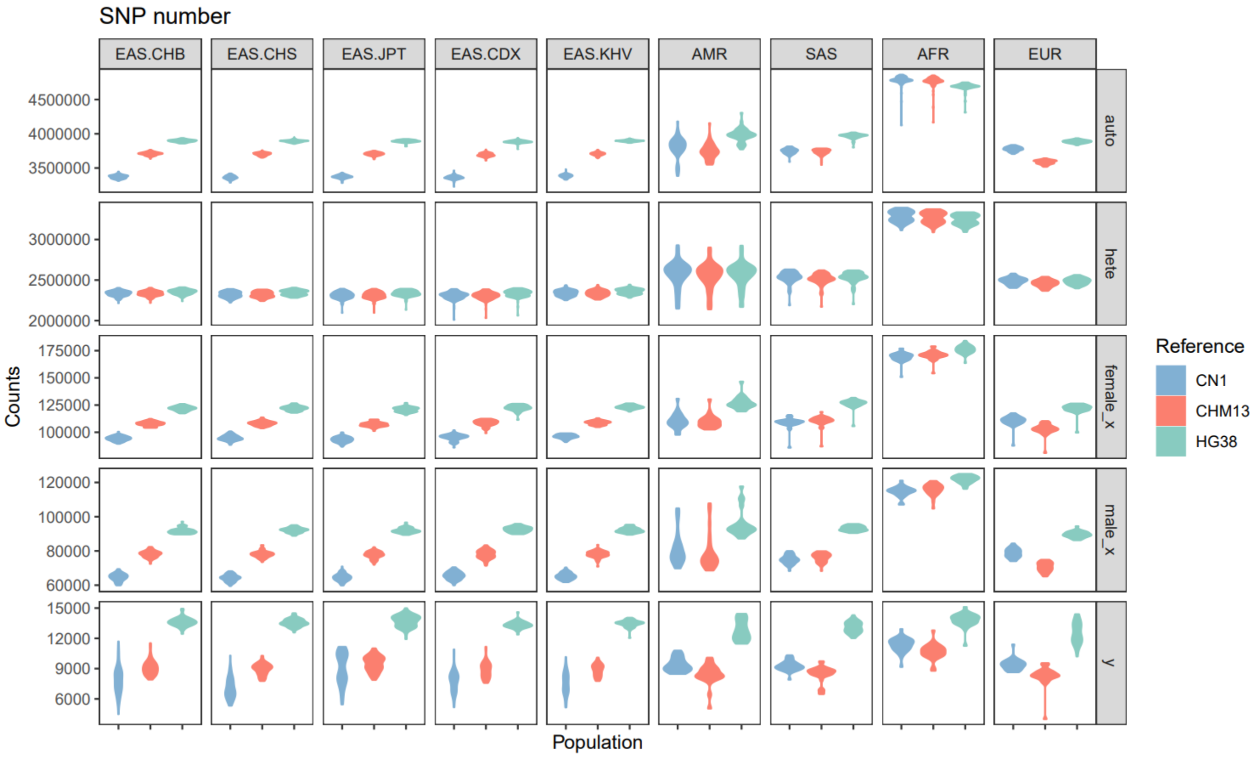

Supplementary information, Fig. S30. The SNP count for different references. The SNV on autosomes, heterozygous SNV, SNV on chrX for females, SNV on chrX for males, and SNV on chrY for males were counted for each population.

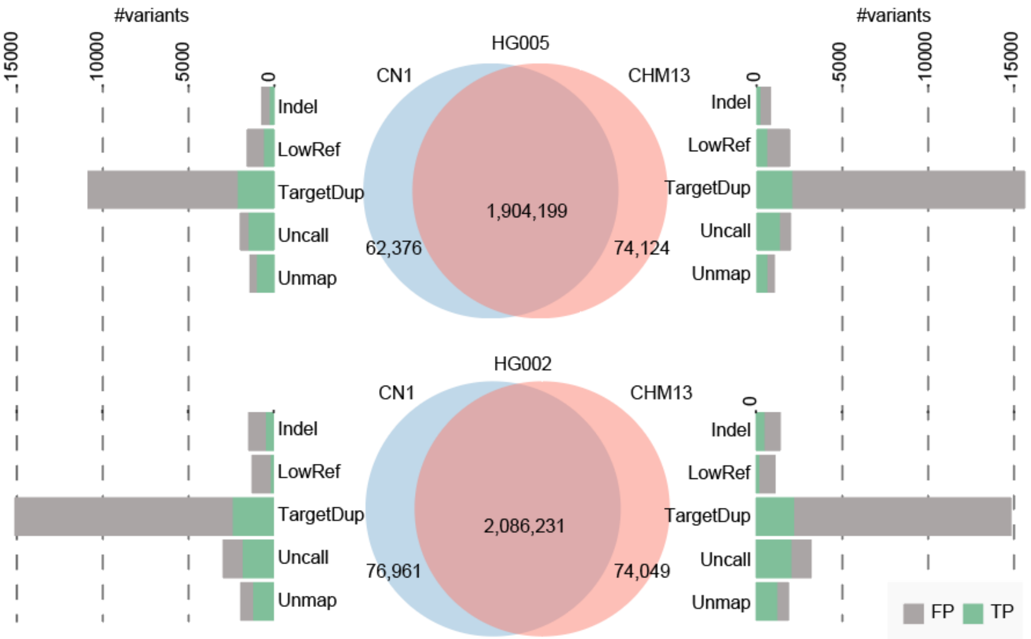

Supplementary information, Fig. S31.The source of unique SNVs for CN1 and CHM13 in HG005 and HG002. The Venn diagram illustrates the number of shared and unique heterozygous SNVs called on CN1 and CHM13. Barplots on the two sides illustrate the count for each unique SNV class, and the number of true positives (TP) and false positives (FP) for each class.

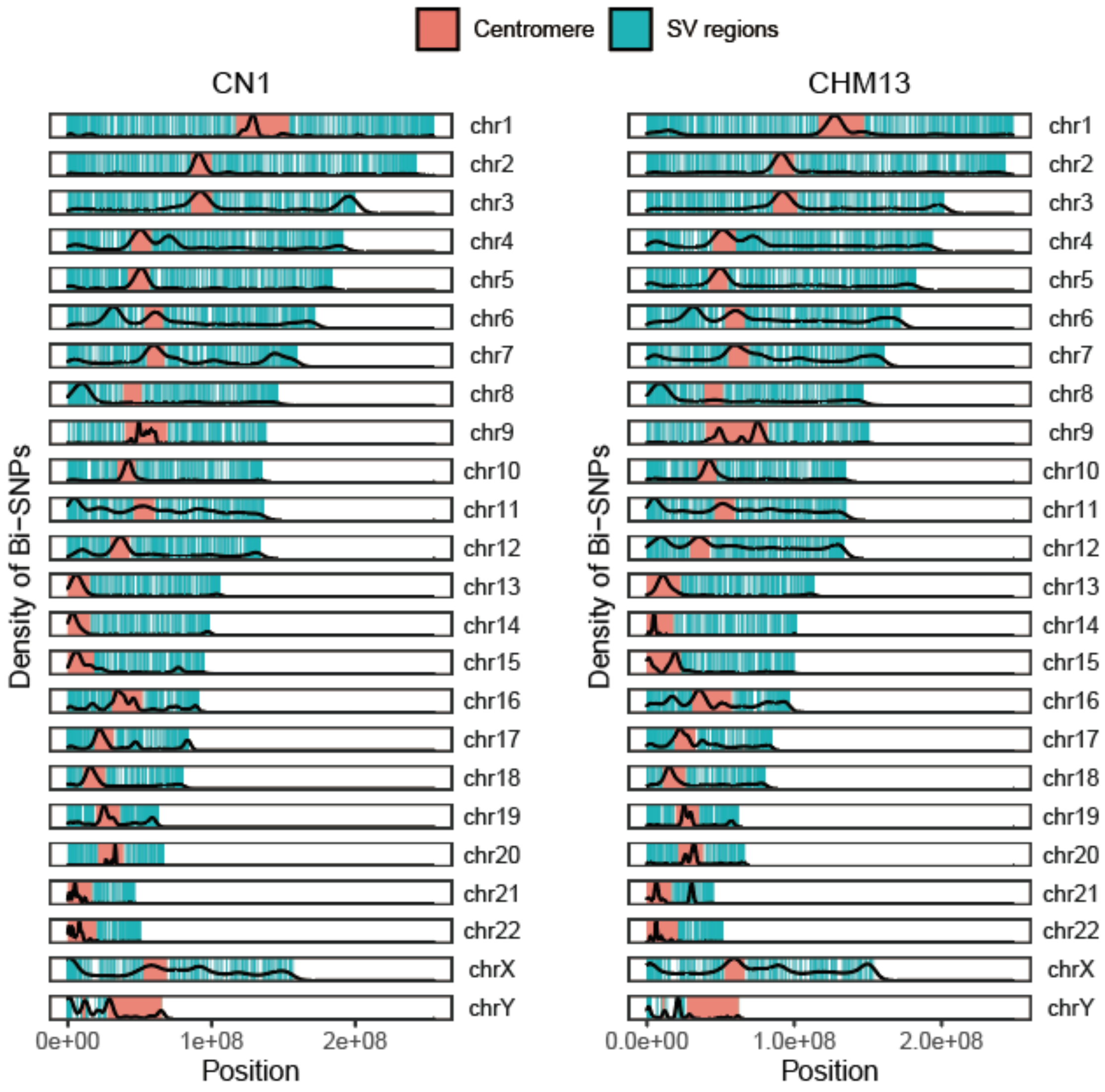

Supplementary information, Fig. S32. Density of CN1 exclusive and CHM13 exclusive bi-SNPs along each chromosome in the corresponding reference. For the CHM13 exclusive SNPs, 45.97% are in centromeres and 10.53% are in SV regions. For the CN1 exclusive SNPs, 52.21% are in centromeres and 10.94% are in SV regions. Red, centromere; blue, SV regions. The analysis is based on the 8,869 genome Chinese cohort.

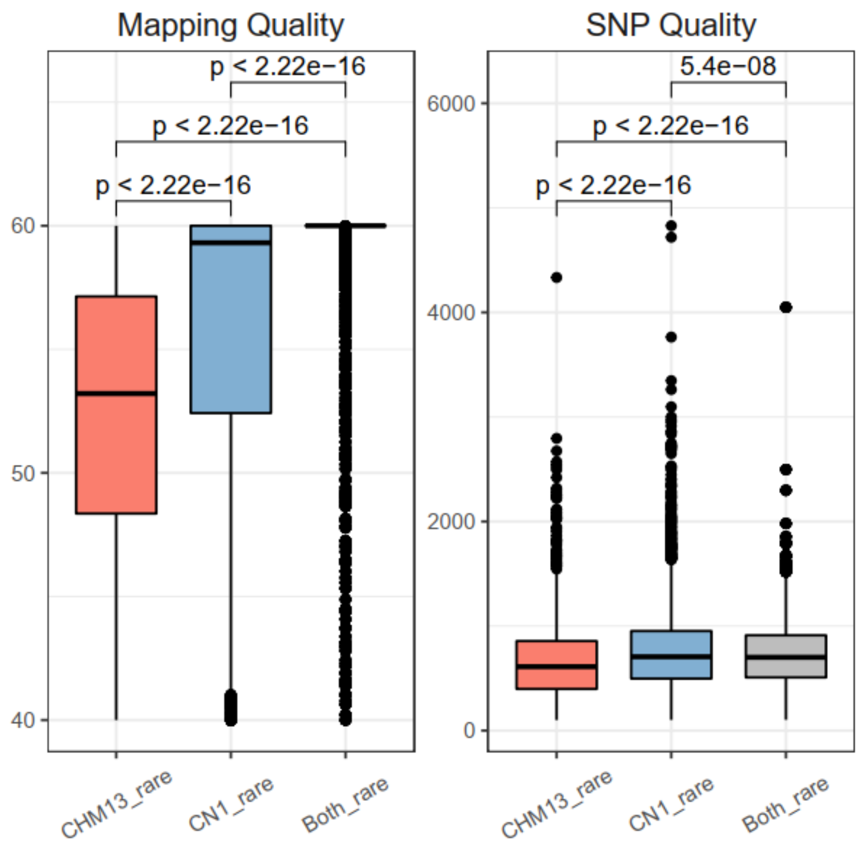

Supplementary information, Fig. S33. Comparison of mapping quality and SNP quality among CHM13_rare, CN1_rare and Both_rare SNPs. Two-sided t-test is performed. The analysis is based on the 8,869 genome Chinese cohort.

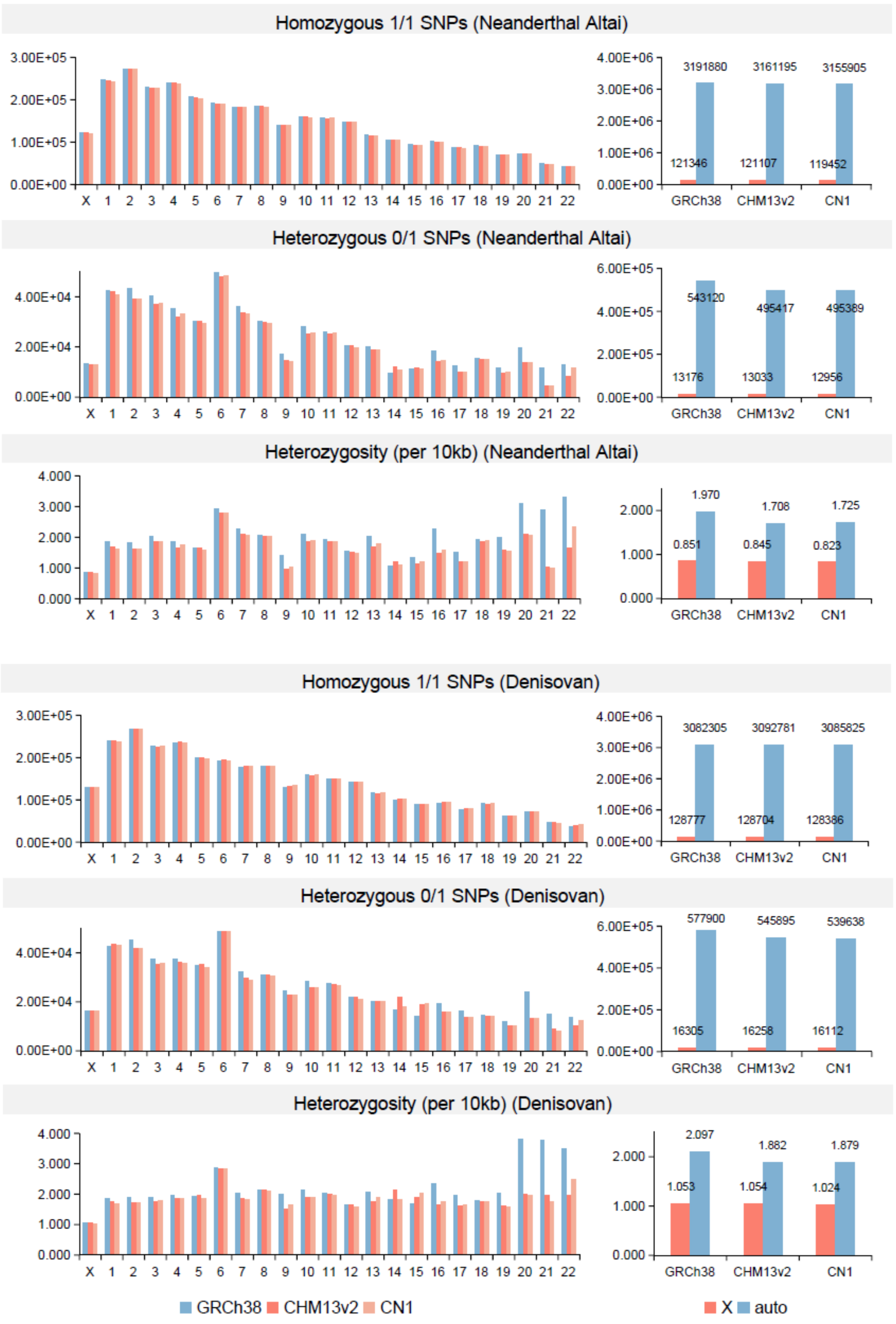

Supplementary information, Fig. S34. Homozygous and heterozygous SNPs in Altai Neanderthal and Denisovan genomes. SNPs were called using CN1, CHM13 and GRCh38 as reference genomes, respectively.

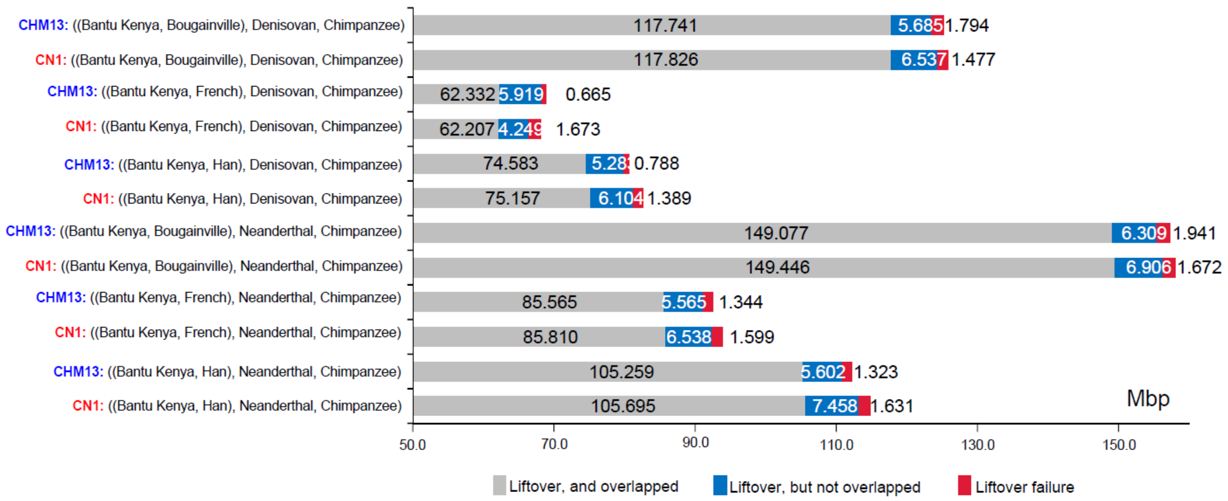

Supplementary information, Fig. S35. Summary of ABBA-BABA tests. In a ABBA-BABA model ((P1, P2), P3, O), P1 was set as Bantu Kenya (*n* = 10), P2 was Han (*n* = 20), French (*n* = 20), or Bougainville (*n* = 10), P3 was Neanderthal or Denisovan genome, and outgroup was chimpanzee (*n* = 5). For a certain quartet, ABBA-BABA tests based on CN1 and CHM13 were performed, respectively. Here $f_d = 0.35$ was set as an empirical cutoff to define putative introgression regions (pIRs). The pIRs using two references were compared by liftover, where gray represented these pIRs were detected using either reference, blue represented that these pIRs could be liftovered to the other reference but were not detected as pIRs using the other reference, and red represented that these pIRs could not liftover to the coordinates in the other reference genome.

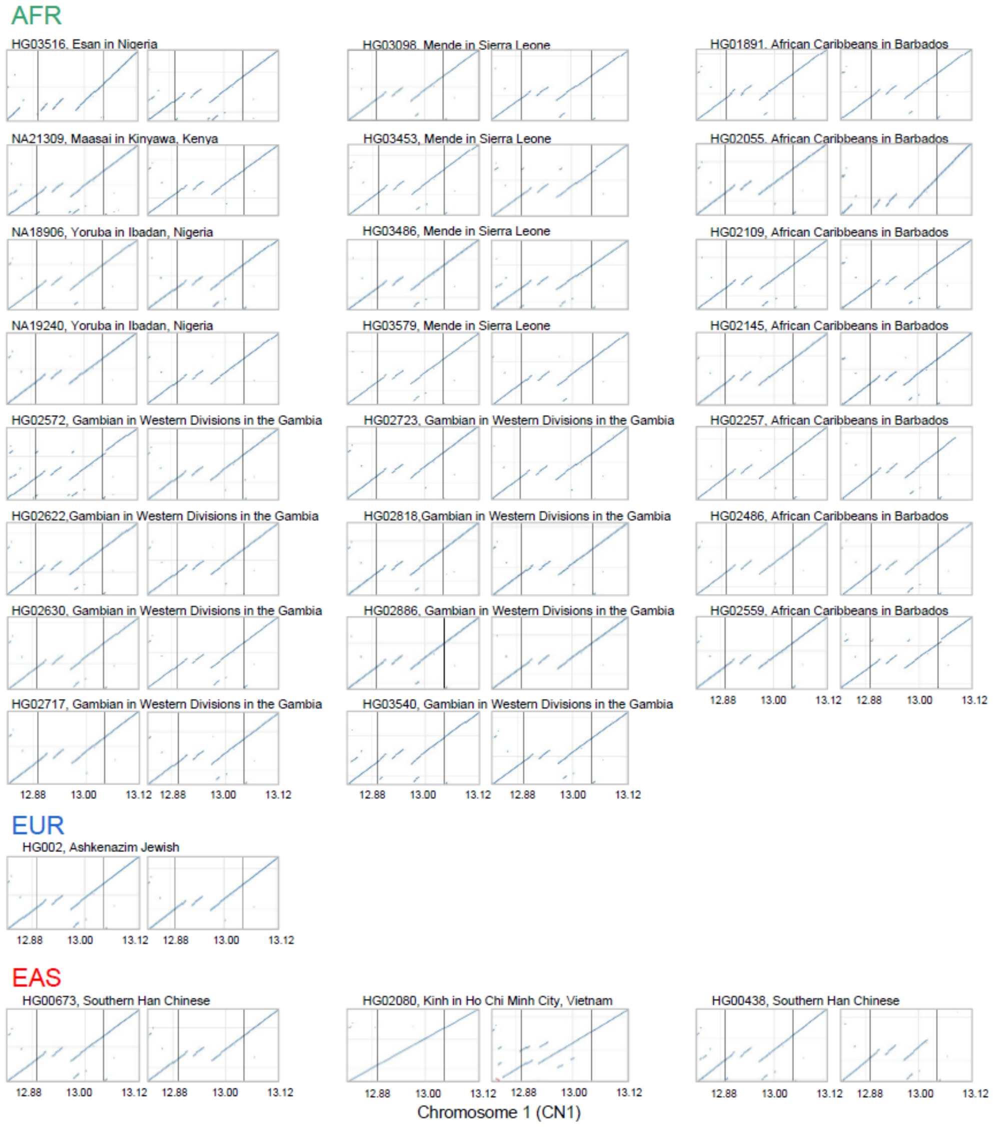

Supplementary information, Fig. S36. Local synteny between CN1 and HPRC assemblies around 13 Mbp on chromosome 1. Interval between two vertical lines indicated a pIR in Han population detected by ABBA-BABA tests using CN1 as reference genome.

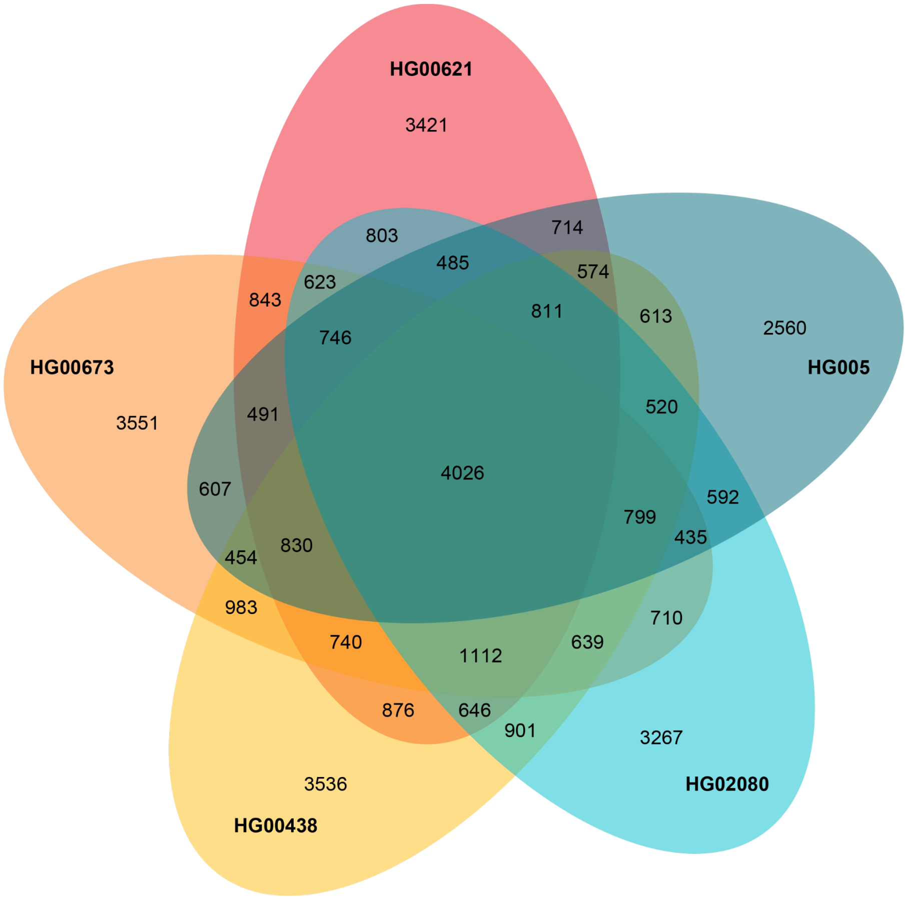

Supplementary information, Fig. S37. SV calling of 5 EAS samples from HPRC using CN1 as reference.
